# CCC-GPU: a graphics processing unit (GPU)-accelerated nonlinear correlation coefficient for large-scale transcriptomic analyses

**DOI:** 10.1093/bioinformatics/btag068

**Published:** 2026-02-13

**Authors:** Haoyu Zhang, Kevin T Fotso, Marc Subirana-Granés, Milton Pividori

**Affiliations:** Department of Biomedical Informatics, University of Colorado Anschutz, Aurora, Colorado, 80045, United States; Information Strategy and Services, University of Colorado Anschutz, Aurora, Colorado, 80045, United States; Department of Biomedical Informatics, University of Colorado Anschutz, Aurora, Colorado, 80045, United States; Department of Biomedical Informatics, University of Colorado Anschutz, Aurora, Colorado, 80045, United States; Colorado Center for Personalized Medicine, University of Colorado Anschutz, Aurora, Colorado, 80045, United States

## Abstract

**Motivation:**

Identifying meaningful patterns in complex biological data necessitates correlation coefficients capable of capturing diverse relationship types beyond simple linearity. Furthermore, efficient computational tools are crucial for handling the ever-increasing scale of biological datasets.

**Results:**

We introduce CCC-GPU, a high-performance, GPU-accelerated implementation of the Clustermatch Correlation Coefficient (CCC). CCC-GPU computes correlation coefficients for mixed data types, effectively detects nonlinear relationships, and offers significant speed improvements over its predecessor.

**Availability and implementation:**

The source code of CCC-GPU is openly available on GitHub (https://github.com/pivlab/ccc-gpu) and archived on Zenodo (https://doi.org/10.5281/zenodo.18310318), distributed under the BSD-2-Clause Plus Patent License.

## 1 Introduction

Correlation coefficients are fundamental tools for uncovering meaningful patterns within data. While traditional measures like Pearson and Spearman are adept at capturing linear and monotonic relationships, newer methodologies, such as the Clustermatch Correlation Coefficient (CCC) (Pividori *et al.* 2024) and the Maximal Information Coefficient (MIC) (Reshef *et al.* 2011) have emerged to detect a broader spectrum of associations. These coefficients represent three distinct statistical assumptions: Pearson captures linear relationships, Spearman measures monotonic patterns using a nonparametric approach, and CCC detects general nonlinear associations. We centered our analysis on these three to effectively cover the most common correlation scenarios without redundancy; notably, we excluded Kendall’s τ due to its high correlation with Spearman’s ρ and ∼540× slower computation.

The CCC is a clustering-based statistic designed to identify both linear and nonlinear patterns. Previously, we demonstrated CCC’s utility in analyzing gene expression data from GTEx, showcasing its robustness to outliers and its ability to detect both strong linear relationships and biologically significant nonlinear patterns often missed by conventional coefficients (Pividori *et al.* 2024). Unlike MIC, CCC accommodates both numerical and categorical data types and is up to two orders of magnitude faster. However, despite leveraging CPU multi-threading for acceleration, the original CCC implementation can still be computationally expensive for large datasets. For instance, in the original study (Pividori *et al.* 2024), we used only the top 5000 most variable genes in a single tissue (whole blood) to ease computation.

Here, we introduce a new implementation, CCC-GPU, that harnesses the power of NVIDIA CUDA for GPU programming. We have computed CCC-GPU values for all ∼50 000 genes in the Genotype-Tissue Expression (GTEx) v8 dataset (The GTEx Consortium 2020) across all 54 tissues in a fraction of the time that would have been needed using the original CCC implementation. This advancement achieves a substantial speedup over the original CPU-based implementation, making comprehensive correlation analysis of large biological datasets more practical and efficient.

## 2 Materials and methods

**Figure 1:**
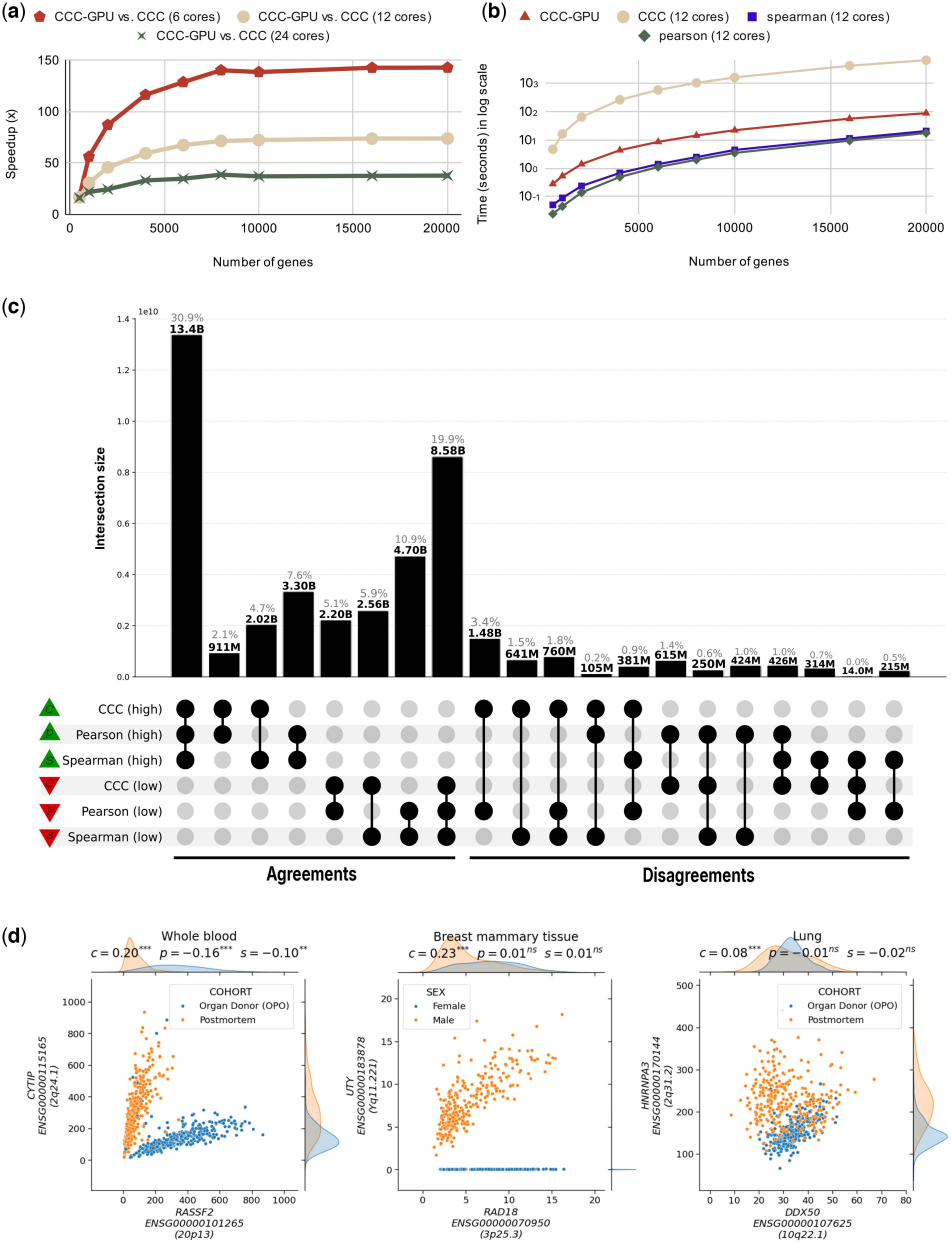
(a) CCC-GPU scalability analysis showing speedup relative to CPU-based CCC across varying gene counts (1000 samples fixed) and CPU core configurations. See Table 1, available as supplementary data at *Bioinformatics* online for detailed metrics. (b) Runtime comparison of correlation methods (Pearson, Spearman, original CCC, CCC-GPU) demonstrating CCC-GPU’s competitive performance despite its complexity to capture nonlinear patterns. Analysis used 12 CPU cores with varying gene counts (1000 samples fixed). See Table 2, available as supplementary data at *Bioinformatics* online. (c) UpSet plot showing correlation agreement (left) and disagreement (right) patterns of all gene pairs in all 54 GTEx v8 tissues among methods. Green triangles indicate high correlations (top 30% gene pairs per tissue), and red triangles show low correlations (bottom 30%). We also created UpSet plots using permutation-based thresholds (see Supplementary Information and Fig. 4, available as supplementary data at *Bioinformatics* online). UpSet plots for individual tissues are provided in Figs 6–59, available as supplementary data at *Bioinformatics* online. (d) Expression levels of selected gene pairs with associations across GTEx tissues: *RASSF2*–*CYTIP*, in whole blood, shows two clear and strong linear patterns across different sample subsets (postmortem versus organ donor). Although all coefficients are statistically significant, Pearson and Spearman detect the wrong pattern (i.e. their slopes are negative); *UTY*–*RAD18* shows a linear relationship only in male samples, whereas female samples exhibit a constant value of zero (since *UTY* is located in chromosome Y). *HNRNPA3*–*DDX50* demonstrates a case where one sample subset (organ donor) shows a linear relationship that is masked by another subset (postmortem) with no or weak relationship.

CCC was originally developed in Python, as detailed in the original publication (Pividori *et al.* 2024). We provide the algorithm equation and pseudocode in Supplementary Information (Equation 1, available as supplementary data at *Bioinformatics* online and Fig. 1, available as supplementary data at *Bioinformatics* online), respectively. Profiling CCC revealed that Adjusted Rand Index (ARI) computation (Hubert and Arabie 1985) was the primary performance bottleneck in our experiments, accounting for 74%–91% of total runtime depending on workload size (Figs 2 and 3, available as supplementary data at *Bioinformatics* online). This is expected as CCC heavily relies on ARI computation for internal data clustering. For example, when computing the pairwise correlations for one GTEx v8 tissue with ∼50 000 genes using default CCC parameters, approximately 1.5 billion ARI calculations are needed. Since individual ARI calculations have no data dependencies and require minimal synchronization, they are ideal candidates for utilizing graphics processing units (GPUs), which contain thousands of compute units designed for massively parallel operations.

We developed a GPU-accelerated implementation by rewriting the core ARI computation module in CUDA C++ (requiring an NVIDIA GPU with CUDA Compute Capability 8.6 or higher, i.e. RTX 30-series or newer). While existing CUDA-based Python libraries were evaluated, native C++ code provides greater flexibility and complete access to CUDA’s feature set (Brodtkorb *et al.* 2020). In this implementation, we replaced the Python-based ARI computation logic with CUDA C++ kernel functions. Each feature (gene) pair’s CCC correlation involves multiple ARI calculations, which we assigned unique global indices. These calculations are carefully mapped to kernel functions by their global indices, then executed concurrently across multiple GPU cores. This required rebuilding internal algorithms that Python libraries such as NumPy conveniently provide. An orchestration layer processes inputs in batches, allocating up to 4.5 GB of GPU memory per batch—well within the capacity of modern GPUs, enabling memory-efficient streaming of large-scale matrices. Within each batch, kernel functions use shared memory for caching and efficient inter-thread communication, device memory buffers are reused across batches to avoid repeated allocation overhead, and results are transferred to host memory upon batch completion. To minimize overhead, device synchronization events occur only at critical points: after each ARI batch computation completes and during *P*-value calculations to ensure kernel completion before data transfer. In summary, our custom solution incorporates GPU architecture optimizations including explicit memory management, tailored caching strategies, and coordinated CPU-GPU communication to maximize performance. Future software versions will expose more parameters for hardware-specific fine-tuning.

We then used pybind11 (https://github.com/pybind/pybind11) to integrate the CUDA C++ backend with the existing Python framework, ensuring full compatibility with the original CCC interface. This hybrid approach preserves the original system’s comprehensive feature set while enabling GPU acceleration, facilitating thorough testing and validation.

## 3 Results

We conducted a comprehensive comparative evaluation of Pearson, Spearman, and CCC-GPU using simulated data and all genes and tissues in the GTEx v8 dataset. The evaluation was performed on an AMD Ryzen Threadripper 7960X CPU with an NVIDIA RTX 4090 GPU, representing a typical high-performance workstation configuration. The original analysis and benchmarking code is available in the GitHub repository (https://github.com/pivlab/ccc-gpu/tree/main/analysis).

We compared the computational complexity of CCC-GPU (using the GPU) and the original CCC (using 24 CPU cores) when analyzing each tissue in the GTEx dataset. For whole blood (56 200 genes, 755 samples), CCC-GPU demonstrated a 37× speedup. On average, the original CCC required approximately 6 h to process one GTEx tissue. This acceleration enabled the complete analysis of all 54 GTEx tissues in just 8 h on a single machine, compared to ∼312 h (∼13 days) that would be needed with the original implementation. Consistent performance gains were observed across all tissue types, confirming CCC-GPU’s broad applicability.

To further understand CCC-GPU’s scalability with increasing data size, we performed benchmarks using synthesized input, comparing its speedup against the original CPU implementation. The results, illustrated in Fig. 1a, demonstrate that CCC-GPU maintains a stable speedup trend as input size increases, confirming its robust scaling capabilities when handling large datasets. The different curves in Fig. 1a simulate CCC-GPU’s prospective speedup over CPU hardware with varying numbers of cores. We also compared the runtime of the original CCC, CCC-GPU, Pearson, and Spearman methods, as shown in Fig. 1b. While Pearson and Spearman are inherently faster due to their reliance on simple statistics, CCC-GPU’s runtime is remarkably close to these methods, showcasing its efficiency while maintaining its advanced capabilities for capturing complex relationships.

The significant performance enhancement provided by CCC-GPU expands the scope of transcriptomic analyses and allowed us to uncover more nonlinear patterns. In the UpSet analysis (Lex *et al.* 2014) shown in Fig. 1c, we compared how Pearson, Spearman, and CCC-GPU agreed or disagreed in prioritizing gene pairs across all genes in the 54 GTEx tissues. We found that ∼2.9 billion gene pairs found only by CCC-GPU (Disagreements in Fig. 1c, where CCC-GPU is “high” and any of the others “low”) likely have biologically meaningful nonlinear patterns, as it was found in the original CCC study (Pividori *et al.* 2024). Likewise, gene pairs where Pearson is “high” and the rest are “low” are likely driven by outliers (Pividori *et al.* 2024).

Leveraging CCC-GPU’s mixed data type capability, we correlated gene expression with GTEx metadata to interpret nonlinear patterns. For this, we included categorical variables such as sex, mortality status, and numerical variables such as BMI, age, among others. From the five intersection groups where CCC values were high but Pearson or Spearman remained low, we selected the top 100 gene pairs per tissue with the largest CCC value. Figure 1d highlights gene pairs with biologically interpretable nonlinear patterns explained by a metadata variable, such as *UTY*–*RAD18* (sex) and *RASSF2*–*CYTIP* [pre/post-mortem status; these two genes were previously found to be differentially expressed in these conditions (Ferreira *et al.* 2018)]. We provide the CCC values computed for all gene pairs, and the top gene-metadata correlation results across all GTEx v8 tissues (see Note 1, available as supplementary data at *Bioinformatics* online).

## 4 Conclusion and discussion

We present CCC-GPU, a GPU-accelerated implementation of the original Clustermatch Correlation Coefficient (CCC). Our work demonstrates that CCC-GPU delivers a remarkable acceleration over its CPU-based predecessor, enabling the rapid and efficient computation of correlation coefficients in large transcriptomic datasets. This performance leap transforms analyses that previously required weeks into tasks achievable within hours on standard research hardware.

Challenges often reside not only in detecting but also in interpreting complex, nonlinear patterns between genes. CCC-GPU’s ability to correlate different data types allows researchers to easily incorporate available metadata into their analyses. For example, a previously highlighted nonlinear relationship for the gene pair *RASSF2*–*CYTIP*, detected by CCC, was explained by GTEx metadata field “COHORT” (pre- versus post-mortem status). These two genes were previously reported to be differentially expressed under these conditions (Ferreira *et al.* 2018). CCC also detected a strong linear pattern between these genes regardless of the organism’s mortality status.

Our new CCC-GPU delivers a next-generation correlation coefficient at a fraction of the computational cost without sacrificing its accessibility, accuracy, or reliability. This significant performance enhancement makes comprehensive correlation analysis of large genomic data practical on standard research hardware. Beyond standard correlation analyses, CCC-GPU empowers biologists to perform sophisticated tasks such as advanced feature selection prior to machine learning model training with large datasets. Moreover, by accelerating the discovery of novel nonlinear relationships in expression data, CCC-GPU helps researchers quickly identify patterns beyond conventional, linear-only relationships. This can potentially uncover new biological insights that would otherwise remain hidden and drive forward our understanding of complex biological systems.

## Supplementary Material

btag068_Supplementary_Data

## Data Availability

The data underlying this article are available in Zenodo at (https://doi.org/10.5281/zenodo.17707872). The software implementation and analysis code are available on GitHub at (https://github.com/pivlab/ccc-gpu) and have been archived in Zenodo at (https://doi.org/10.5281/zenodo.18310318).

## References

[btag068-B1] Brodtkorb AR , Hagen TR, Saetra ML. Graphics processing unit (GPU) programming strategies and trends in GPU computing. In: *Proceedings of the 2020 28th Euromicro International Conference on Parallel, Distributed and Network-Based Processing (PDP).* 2020 Mar 11-13; Västerås, Sweden. Piscataway, NJ: IEEE, 2020, 11–18. 10.1109/PDP50117.2020.00041

[btag068-B2] Ferreira PG , Muñoz-Aguirre M, Reverter F et al The effects of death and post-mortem cold ischemia on human tissue transcriptomes. Nat Commun 2018;9:490. 10.1038/s41467-017-02772-x 29440659 PMC5811508

[btag068-B3] Hubert L , Arabie P. Comparing partitions. J Classif 1985;2:193–218. 10.1007/bf01908075

[btag068-B5] Lex A , Gehlenborg N, Strobelt H et al Upset: visualization of intersecting sets. IEEE Trans Vis Comput Graph 2014;20:1983–92. 10.1109/tvcg.2014.2346248 26356912 PMC4720993

[btag068-B6] Pividori M , Ritchie MD, Milone DH et al An efficient not-only-linear correlation coefficient based on clustering. Cell Syst 2024;15:854–708.e10. 10.1016/j.cels.2024.08.005 39243756 PMC11951854

[btag068-B7] Reshef DN , Reshef YA, Finucane HK et al Detecting novel associations in large data sets. Science 2011;334:1518–24. 10.1126/science.1205438 22174245 PMC3325791

[btag068-B8] The GTEx Consortium. The gtex consortium atlas of genetic regulatory effects across human tissues. Science 2020;369:1318–30. 10.1126/science.aaz1776 32913098 PMC7737656

