# Supplementary material for: CCC-GPU: a graphics processing unit (GPU)-accelerated nonlinear correlation coefficient for large-scale transcriptomic analyses
Source: Bioinformatics. 2026 Feb 13;42(3):btag068. doi: 10.1093/bioinformatics/btag068 (PMC12980328; doi:10.1093/bioinformatics/btag068)
Supplement: btag068_Supplementary_Data [file btag068_supplementary_data.pdf]

Supplementary Information

Supplementary Note 1: Datasets

In our Zenodo archive (https://doi.org/10.5281/zenodo.17156519), we provide:

1. the CCC values for all gene pairs of GTEx v8 whole blood (other tissues are omitted since the total file size is too large, but they can be easily computed using the code in the GitHub repository),
2. the threshold tables for top and bottom genes using both the 30% threshold and the permutation-based thresholds (see section below for an explanation of how these thresholds were computed), and
3. the top gene-metadata correlation results for all tissues.

Supplementary Note 2: High and Low Correlation Thresholds

We used two approaches to define correlation values that are "high" or "low" for each coefficient per tissue. The first approach captures, for each tissue and correlation coefficient, the top 30% of gene pairs by using the 70th percentile of correlation values, and the bottom 30% of gene pairs by using the 30th percentile (Figure 1c, in the main text, for all tissues combined; and panel c for individual tissues in the supplementary figures section below). The second approach computes the null distribution of coefficient values per tissue by taking a random subset of 10,000 genes and shuffling samples. Then, we define genes with "high" correlation as those with coefficient values larger than the 95th percentile ($P < 0.05$), and "low" correlation as those with coefficient values smaller than the 80th percentile ($P >$ 0.20) (Figure S5 for all tissues, and panel d for individual tissues, in the supplementary figures section below).

Definition

The *Clustermatch Correlation Coefficient (CCC)* between a feature pair (i.e., gene pair in the context of transcriptomics), represented by data vectors $\mathbf{x}$ and $\mathbf{y}$, is defined as the maximum ARI between all possible object partitions (i.e., grouping of objects, which are groups of RNA-seq samples in the context of transcriptomics) derived from the data vectors:

$$\mathrm{CCC}(\mathbf{x}, \mathbf{y}) = \max\{0, \max_{\substack{\pi_j \in \Pi^{\mathbf{x}} \\ \pi_l \in \Pi^{\mathbf{y}}}} \{\mathrm{ARI}(\pi_j, \pi_l)\}\}, \forall\, |\pi| \in [2, k_{\mathrm{max}}] \quad \textbf{(E1)}$$

where $\Pi^{\mathbf{x}}$ is a set of partitions derived from $\mathbf{x}$, $\Pi^{\mathbf{y}}$ is a set of partitions derived from $\mathbf{y}$, and k_{max} specifies the maximum number of clusters allowed for partitions. The ARI has an upper bound of 1 (achieved when both partitions are identical), and although it does not have a well-defined lower bound, values equal or less than zero are achieved when partitions are independent. Therefore, $\mathrm{CCC}(\mathbf{x}, \mathbf{y}) \in [0, 1]$. In the special case where all n objects in either $\mathbf{x}$ or $\mathbf{y}$ have the same value, the CCC is undefined. Refer to the original CCC article [1] for extended definitions, explanation of k_{max}, properties, statistical significance, among other details.

Pseudocode

Algorithm 1: CCC algorithm

1 **Function** `get_partitions`($\mathbf{v}$, $k_{\max}$):
  Input:
    $\mathbf{v}$: feature values on n objects
    $k_{\max}$: maximum number of clusters
  Output:
    Π: a set of partitions over n objects

2   **if** $\mathbf{v} \in \mathbb{R}^n$ **then**
3   **for** $k \leftarrow 2$ **to** $\min\{k_{\max}, n-1\}$ **do**
4       $\boldsymbol{\rho} \leftarrow (\rho_\ell \mid \Pr(v_i < \rho_\ell) \leq (\ell-1)/k), \forall \ell \in [1, k+1]$
5       $\pi_\ell \leftarrow \{i \mid \rho_\ell < v_i \leq \rho_{\ell+1}\}, \forall \ell \in [1, k]$
6       $\Pi_k \leftarrow \pi$
7   **else**
8     $\mathcal{C} \leftarrow \{c_1, c_2, \ldots, c_m\}$ (set of m unique categorical values in $\mathbf{v}$)
9     $\pi_\ell \leftarrow \{i \mid v_i = \mathcal{C}_\ell\}, \forall \ell \in [1, m]$
10     $\Pi_m \leftarrow \pi$
11   $\Pi \leftarrow \{\Pi_k \mid |\Pi_k| > 1\}, \forall k$
12   **return** Π
13
14 **Function** `ccc`($\mathbf{x}$, $\mathbf{y}$, $k_{\max}$):
  Input:
    $\mathbf{x}$: feature values on n objects
    $\mathbf{y}$: feature values on n objects
    $k_{\max}$: maximum number of clusters
  Output:
    c: correlation value for $\mathbf{x}$ and $\mathbf{y}$ ($c \in [0, 1]$)
15   $\Pi^{\mathbf{x}} = \text{get\_partitions}(\mathbf{x}, k_{\max})$
16   $\Pi^{\mathbf{y}} = \text{get\_partitions}(\mathbf{y}, k_{\max})$
17   $c \leftarrow \max\{\text{ARI}(\pi_j, \pi_l)\}, \forall \pi_j \in \Pi^{\mathbf{x}}, \pi_l \in \Pi^{\mathbf{y}}$
18   **return** $\max(c, 0)$

Figure S1: The CCC algorithm.

The main function of the algorithm, `ccc`, generates a set of partitions $\Pi^{\mathbf{x}}$ for variable $\mathbf{x}$ (line 15), and another set of partitions $\Pi^{\mathbf{y}}$ for variable $\mathbf{y}$ (line 16). Then, it computes the ARI between each partition $\pi_j \in \Pi^{\mathbf{x}}$ and $\pi_l \in \Pi^{\mathbf{y}}$ and gets the maximum (line 17), returning either this value or zero if this is negative (line 18). Refer to the original CCC article [1] for more details.

Supplementary Note 3: CCC Runtime Profiling

To quantify the computational bottleneck in the CCC algorithm, we performed comprehensive profiling using Python's cProfile on representative workloads. We categorized all function calls into: ARI (Adjusted Rand Index computation), Partitioning (quantile-based clustering), Coordination (algorithm orchestration), and NumPy/Numba (numerical operations).

Figure S2 shows the runtime breakdown by individual function for a medium workload (2,500 features × 500 samples). The three ARI-related functions (`adjusted_rand_index`, `get_pair_confusion_matrix`, and `get_contingency_matrix`) collectively consume approximately 80% of total runtime, confirming that ARI computation is the dominant bottleneck.

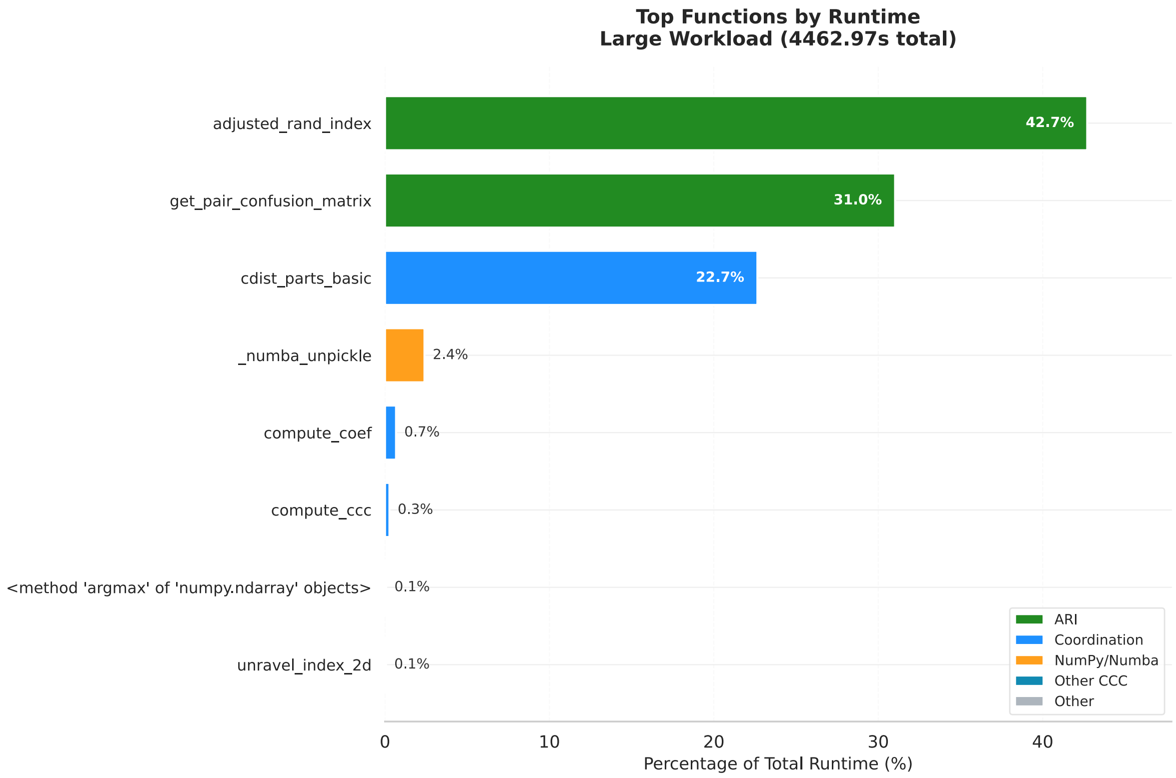

Figure S2: Runtime breakdown by function for CCC computation. Top functions sorted by percentage of total runtime for a 2,500 features × 500 samples workload.

Figure S3 compares runtime distribution across three feature sizes: Small (500 features), Medium (2,500 features), and Large (5,000 features), all with 500 samples. The ARI category consistently dominates across all workload sizes, accounting for ~74% of runtime.

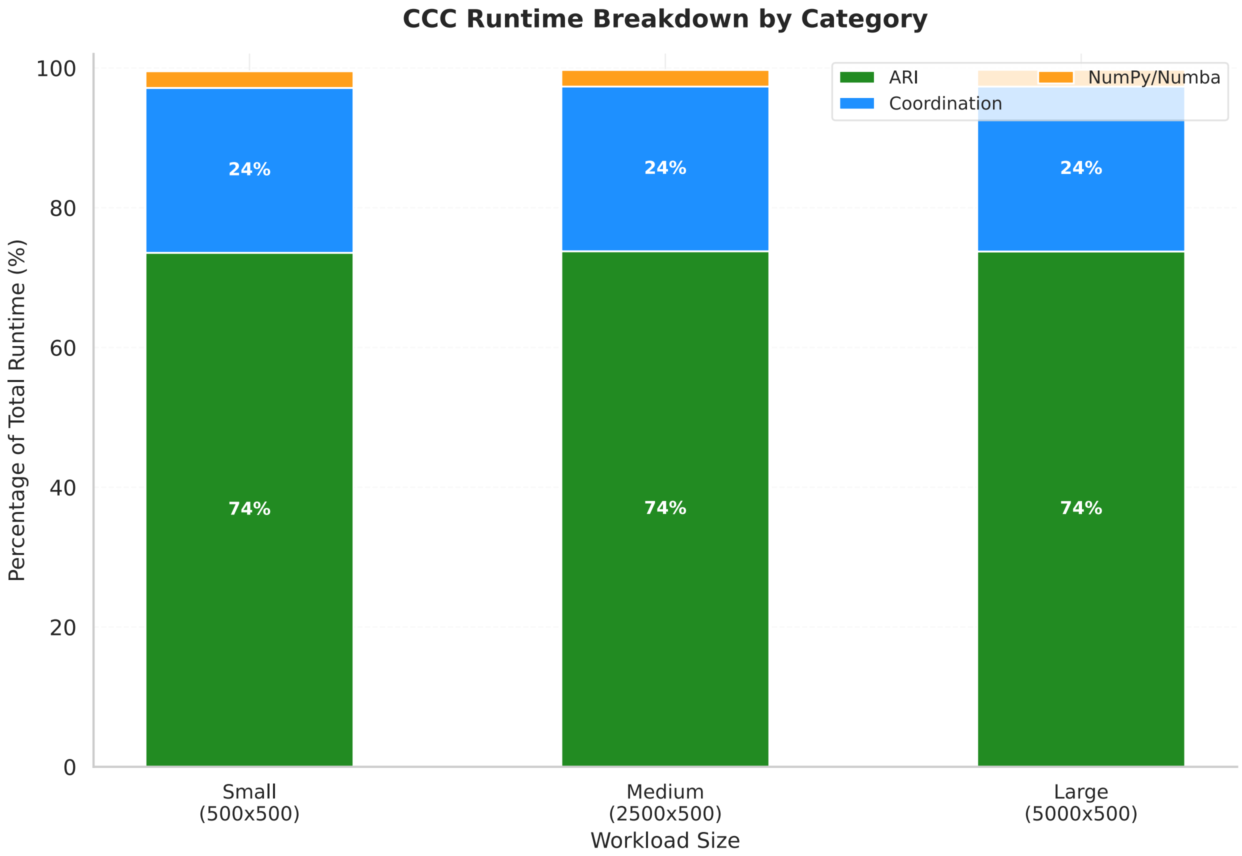

Figure S3: Category breakdown comparison across different feature sizes. Runtime distribution for Small (500 features), Medium (2,500 features), and Large (5,000 features) workloads, each with 500 samples.

Figure S4 shows how ARI's share of total runtime changes as sample count increases (500 to 4,000 samples, fixed 500 features). The ARI percentage increases from approximately 74% at 500 samples to over 91% at 4,000 samples, demonstrating that for larger datasets typical in transcriptomics, ARI computation becomes even more dominant. This analysis confirms that GPU acceleration of ARI computation addresses the correct computational bottleneck.

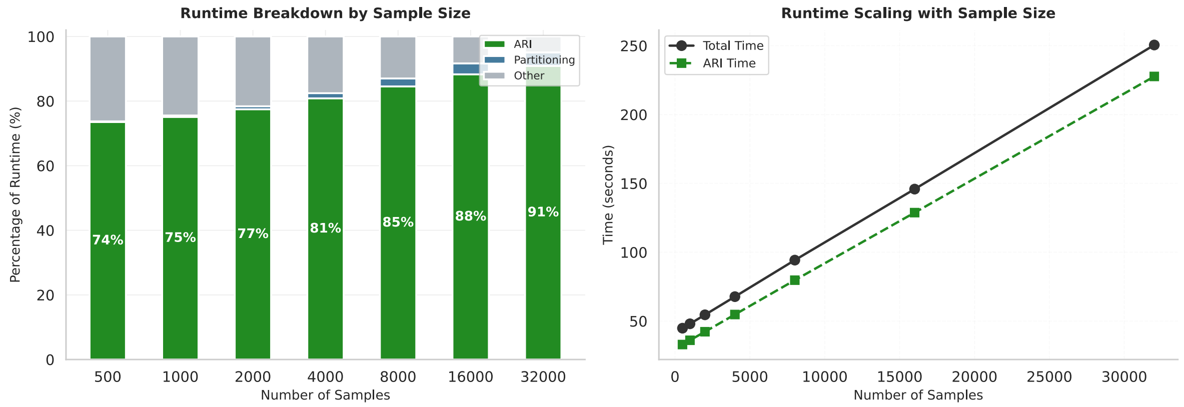

Figure S4: ARI runtime percentage scaling with sample size. Percentage of total CCC runtime spent on ARI computation as sample count increases from 500 to 4,000 (fixed 500 features).

Tables

Table S1: Speedup using different CPU configurations (1,000 fixed samples).

Number of genes	CCC-GPU vs. CCC (6 cores)	CCC-GPU vs. CCC (12 cores)	CCC-GPU vs. CCC (24 cores)
500	17.6x	16.52x	16.1x
1,000	56.17x	30.65x	21.82x
2,000	87.06x	45.72x	24.45x
4,000	116.39x	59.46x	33.03x
6,000	128.74x	67.46x	34.77x
8,000	140.24x	71.48x	38.67x
10,000	138.51x	72.38x	37.02x
16,000	142.7x	73.83x	37.53x
20,000	142.83x	73.88x	37.73x

Table S2: Execution time of different methods (1,000 fixed samples).

Number of genes	CCC-GPU	CCC (12 cores)	Spearman (12 cores)	Pearson (12 cores)
500	0.279s	4.603s	0.051s	0.024s
1,000	0.529s	16.198s	0.089s	0.045s
2,000	1.384s	63.263s	0.235s	0.138s
4,000	4.290s	255.071s	0.679s	0.489s
6,000	8.441s	569.409s	1.379s	1.093s
8,000	14.116s	1009.069s	2.443s	1.973s
10,000	21.694s	1570.269s	4.379s	3.448s

Number of genes	CCC-GPU	CCC (12 cores)	Spearman (12 cores)	Pearson (12 cores)
16,000	55.469s	4051.114s	11.169s	9.310s
20,000	86.286s	6374.781s	20.282s	17.238s

Figures

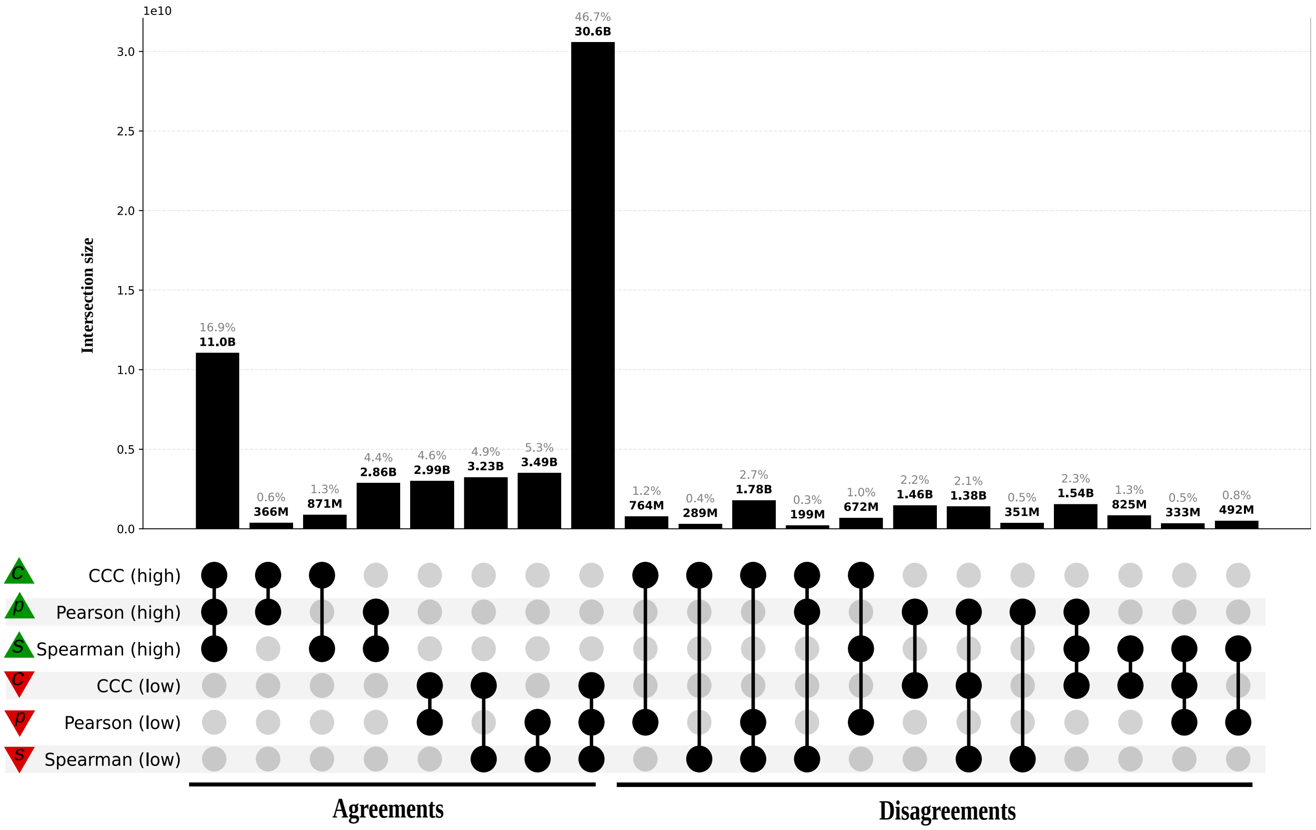

Figure S5: UpSet plot for gene pairs in all 54 tissues in GTEx using permutation-based thresholds for each coefficient for grouping.

Adipose Subcutaneous

a) Correlation coefficient distributions between gene pairs within GTEx v8 Adipose Subcutaneous

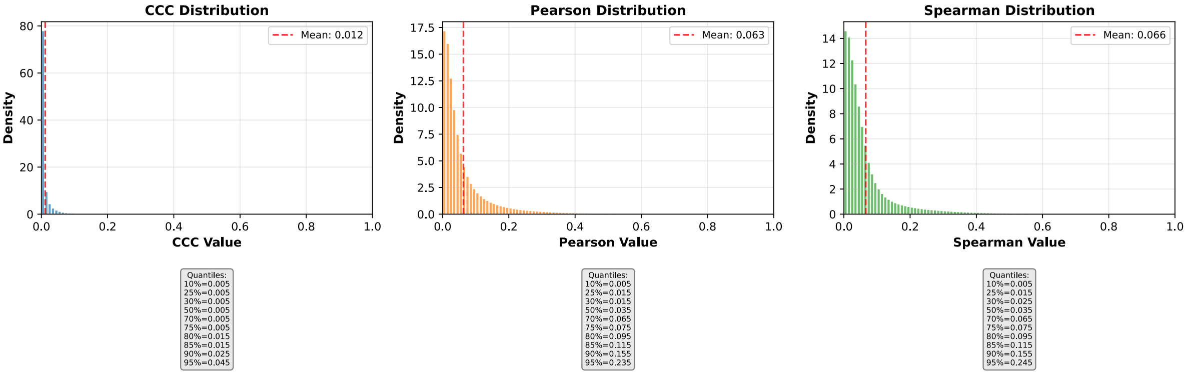

b) Corresponding cumulative histogram

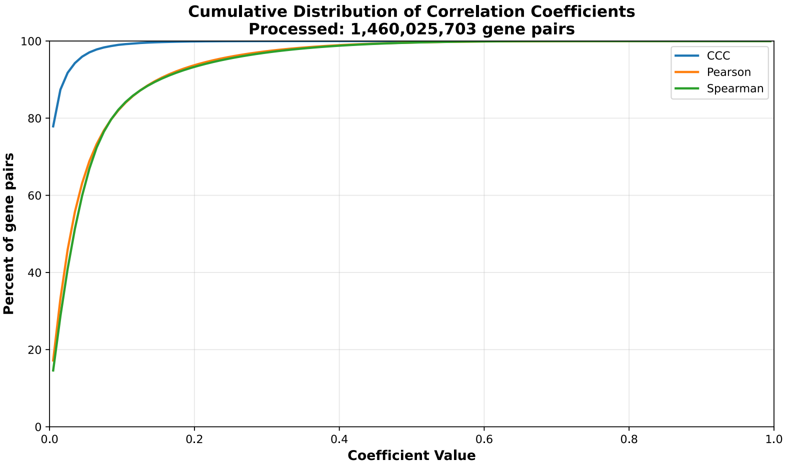

c) UpSet plot using top and bottom 30% correlations

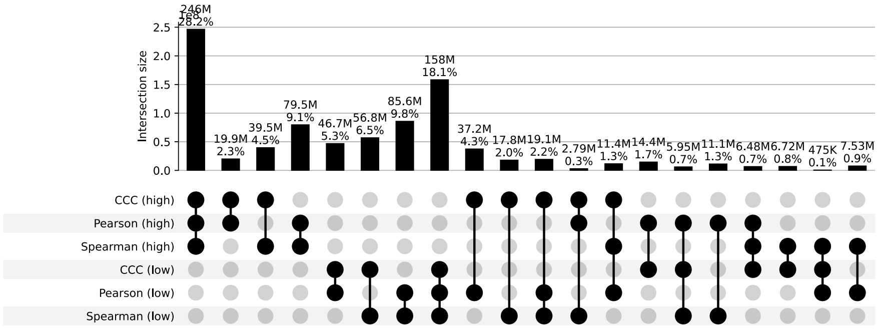

d) UpSet plot using permutation-based statistical thresholds

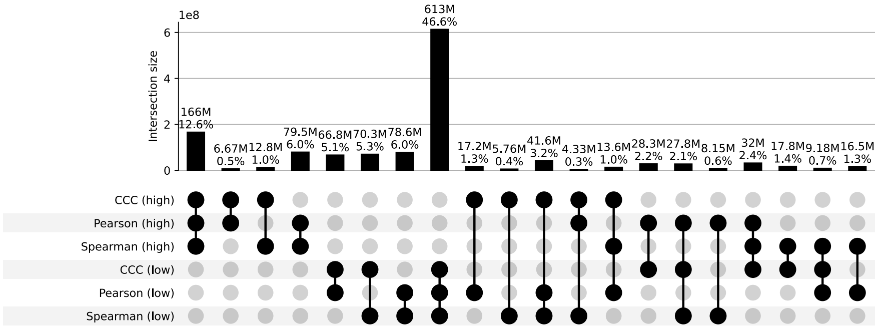

Figure S6: Distribution and UpSet plots for GTEx v8 adipose subcutaneous.

Adipose Visceral Omentum

a) Correlation coefficient distributions between gene pairs within GTEx v8 Adipose Visceral Omentum

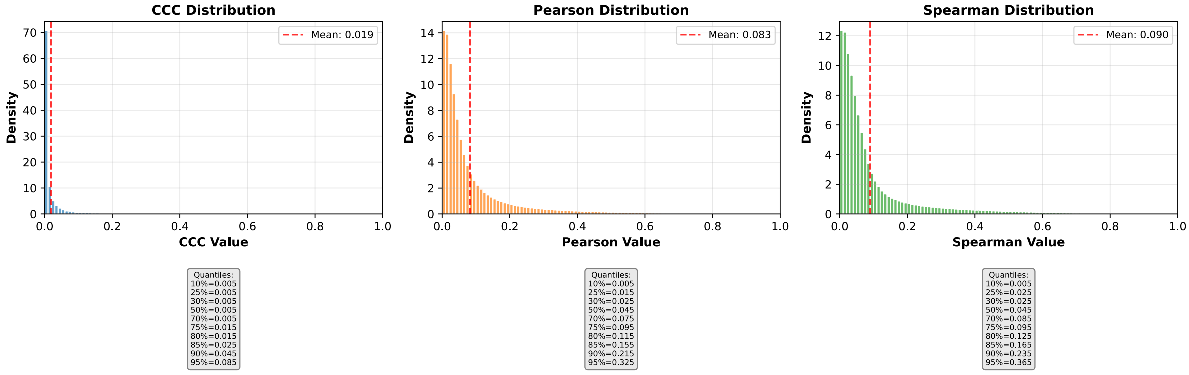

b) Corresponding cumulative histogram

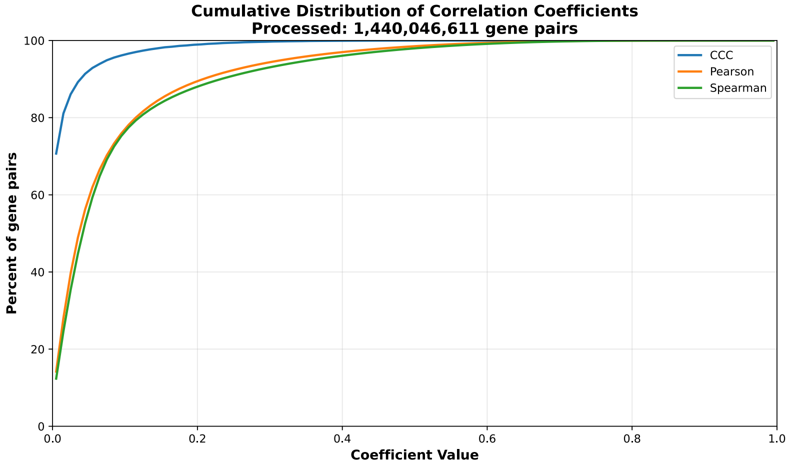

c) UpSet plot using top and bottom 30% correlations

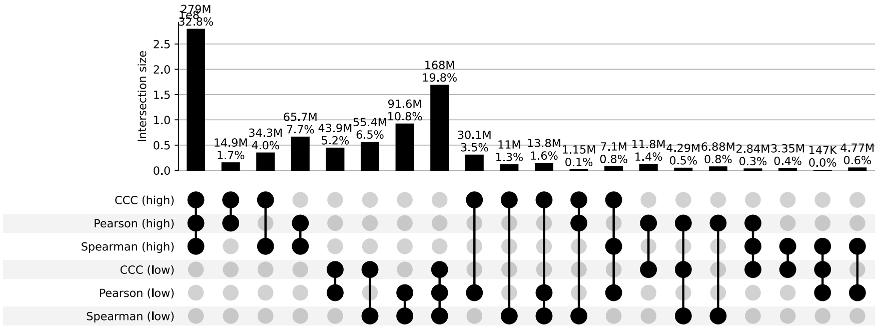

d) UpSet plot using permutation-based statistical thresholds

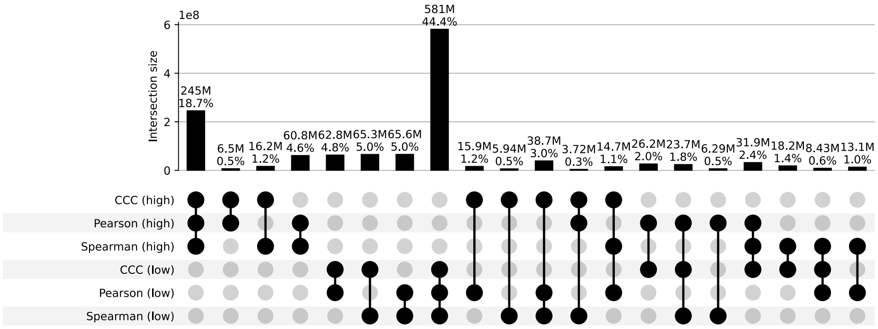

Figure S7: Distribution and UpSet plots for GTEx v8 adipose visceral omentum.

Adrenal Gland

a) Correlation coefficient distributions between gene pairs within GTEx v8 Adrenal Gland

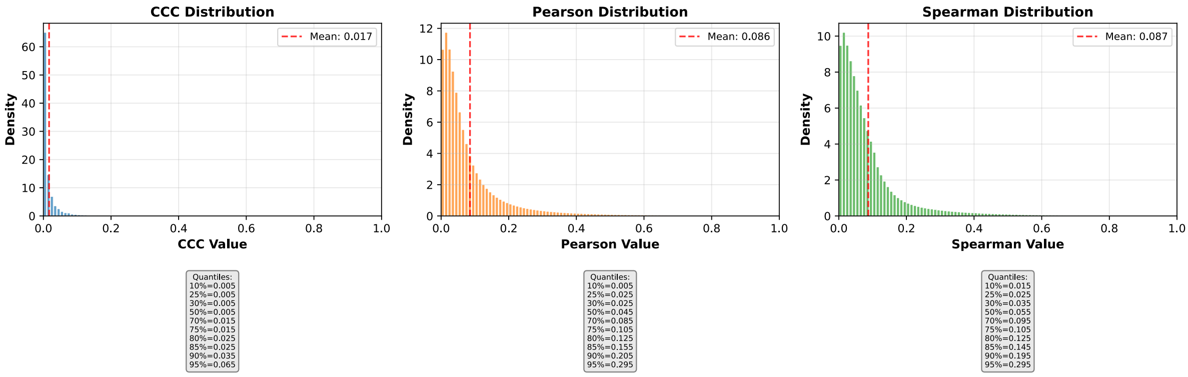

b) Corresponding cumulative histogram

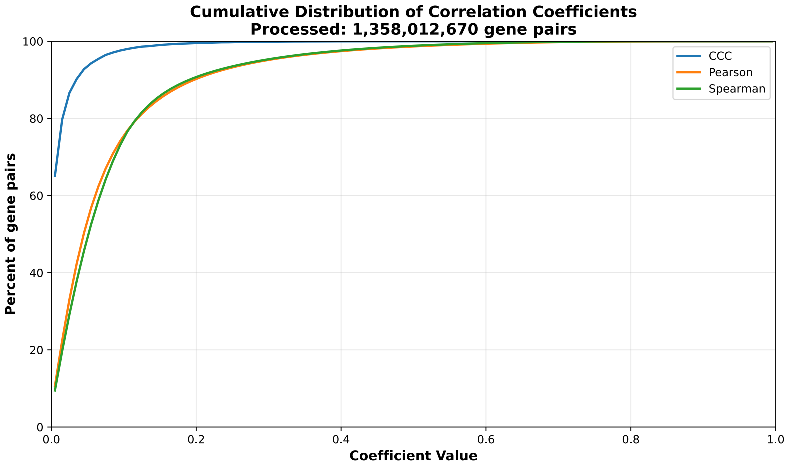

c) UpSet plot using top and bottom 30% correlations

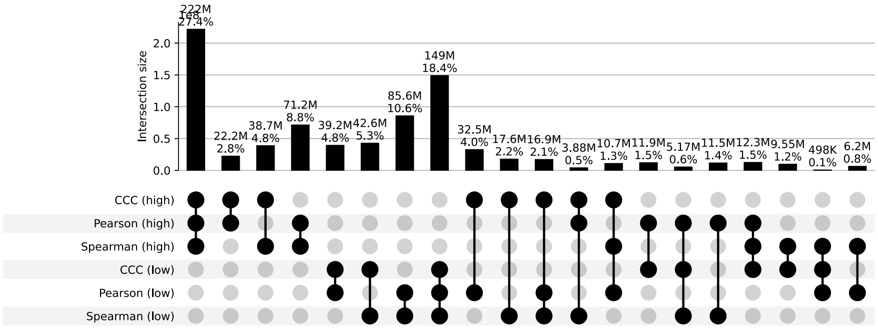

d) UpSet plot using permutation-based statistical thresholds

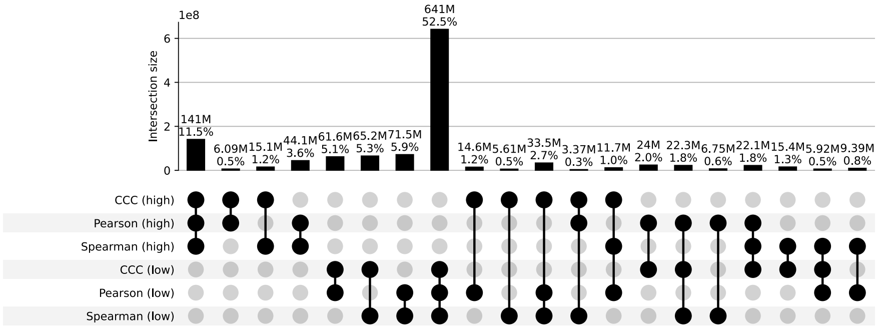

Figure S8: Distribution and UpSet plots for GTEx v8 adrenal gland.

Artery Aorta

a) Correlation coefficient distributions between gene pairs within GTEx v8 Artery Aorta

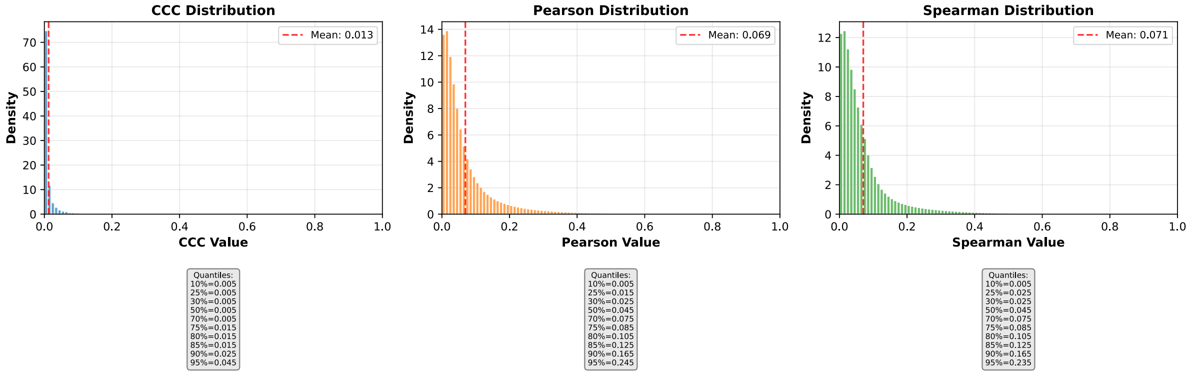

b) Corresponding cumulative histogram

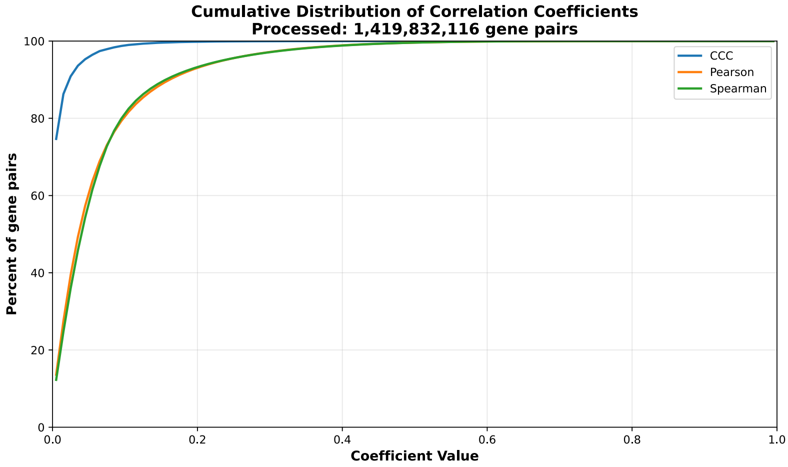

c) UpSet plot using top and bottom 30% correlations

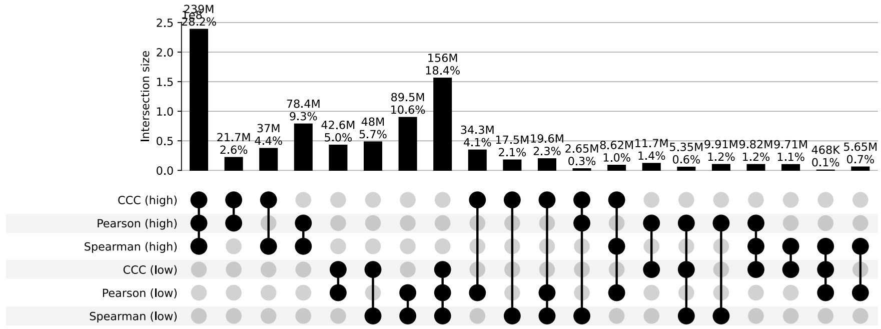

d) UpSet plot using permutation-based statistical thresholds

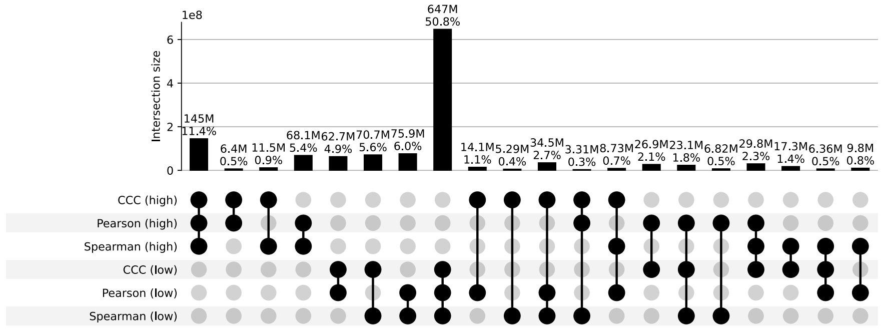

Figure S9: Distribution and UpSet plots for GTEx v8 artery aorta.

Artery Coronary

a) Correlation coefficient distributions between gene pairs within GTEx v8 Artery Coronary

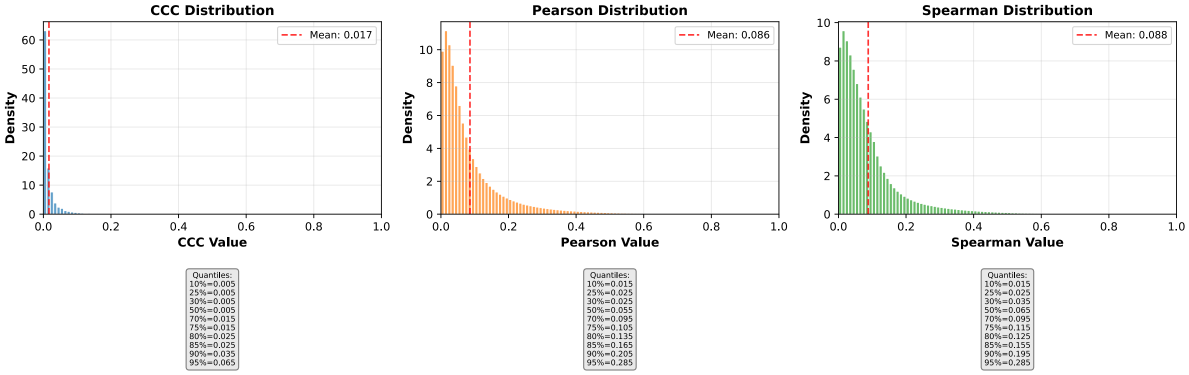

b) Corresponding cumulative histogram

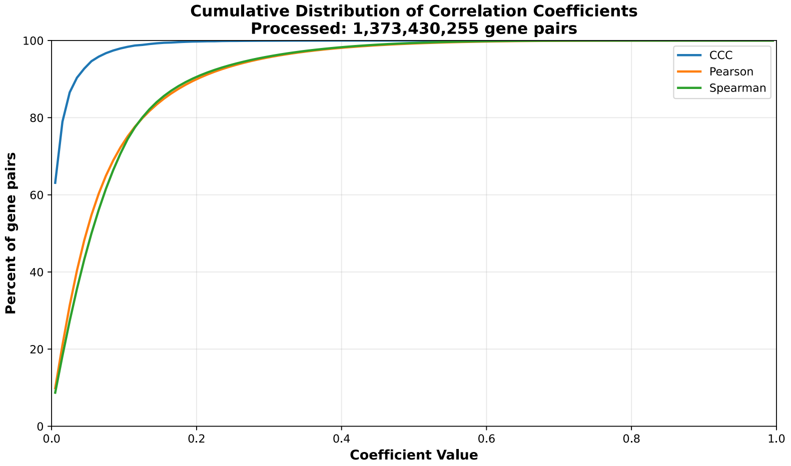

c) UpSet plot using top and bottom 30% correlations

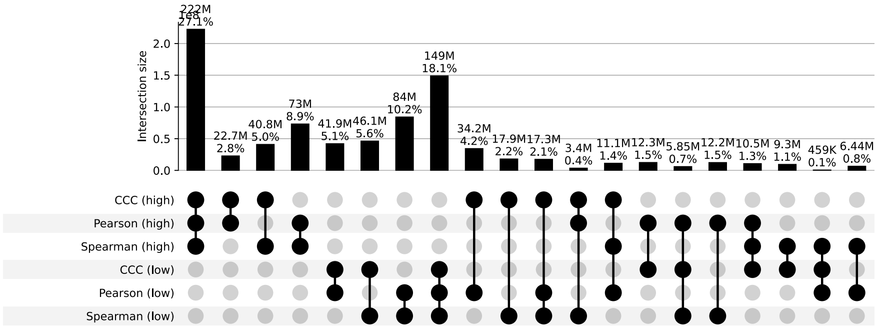

d) UpSet plot using permutation-based statistical thresholds

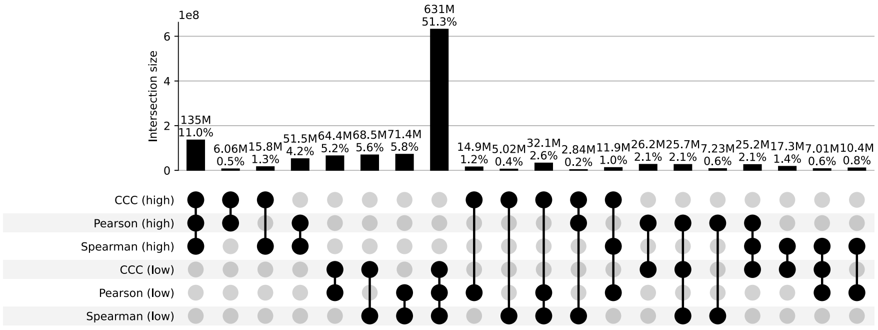

Figure S10: Distribution and UpSet plots for GTEx v8 artery coronary.

Artery Tibial

a) Correlation coefficient distributions between gene pairs within GTEx v8 Artery Tibial

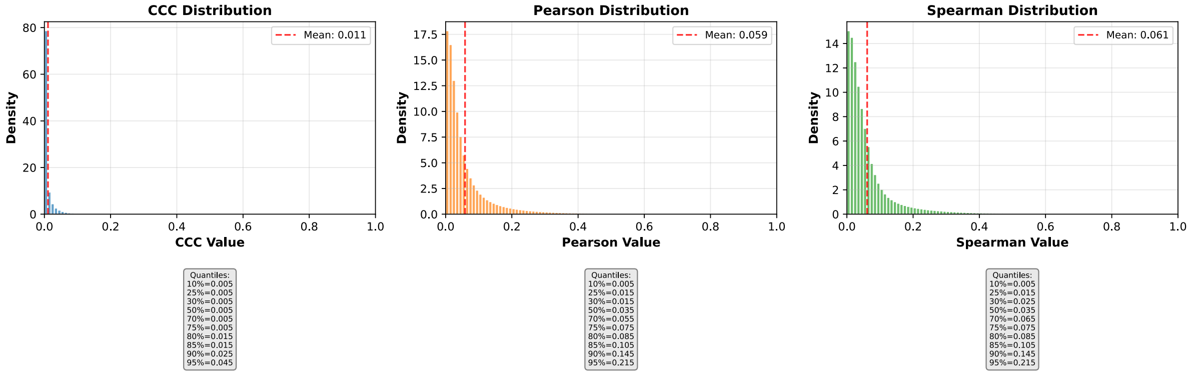

b) Corresponding cumulative histogram

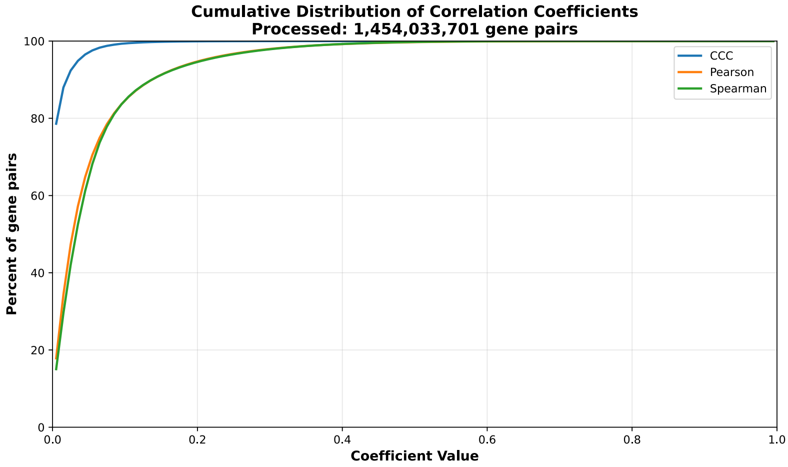

c) UpSet plot using top and bottom 30% correlations

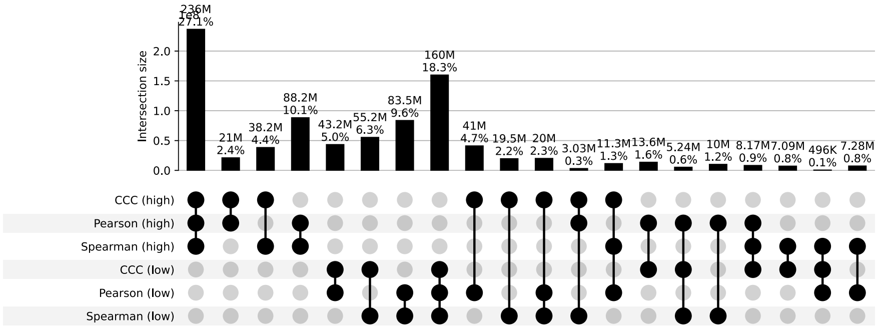

d) UpSet plot using permutation-based statistical thresholds

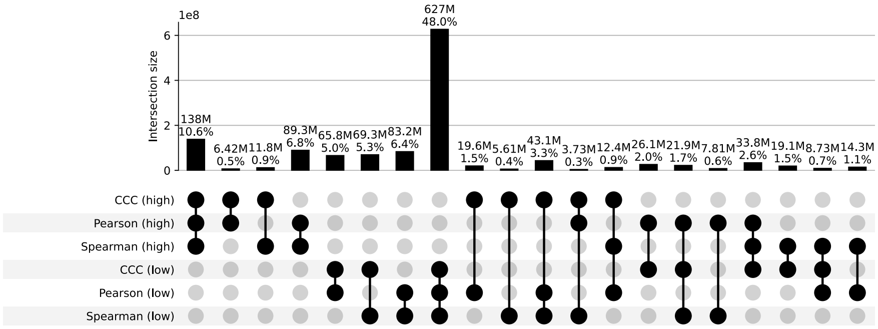

Figure S11: Distribution and UpSet plots for GTEx v8 artery tibial.

Bladder

a) Correlation coefficient distributions between gene pairs within GTEx v8 Bladder

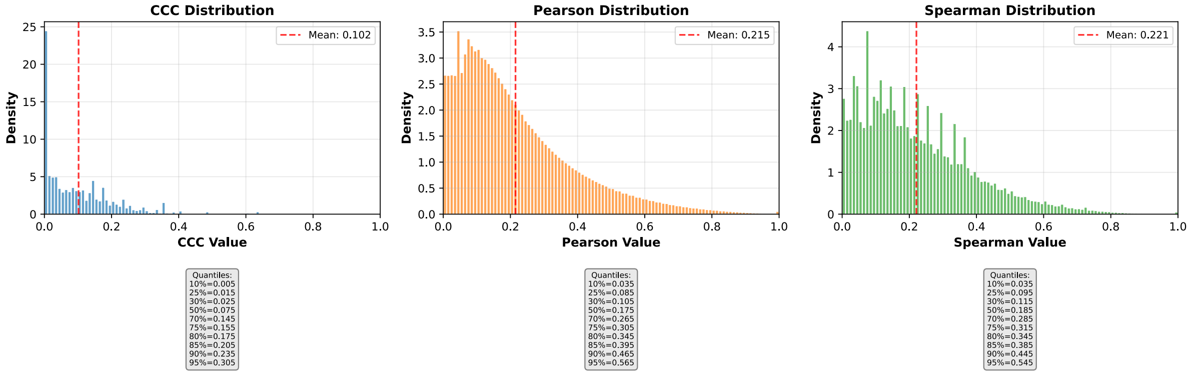

b) Corresponding cumulative histogram

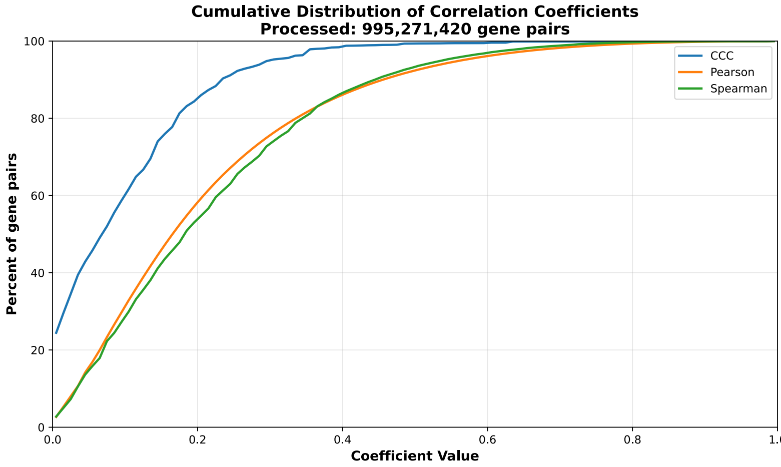

c) UpSet plot using top and bottom 30% correlations

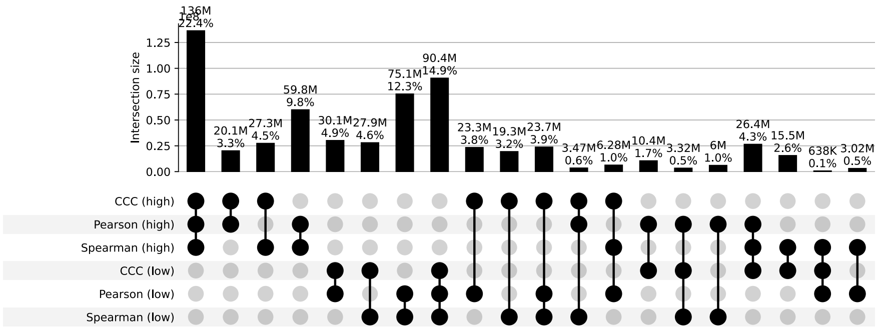

d) UpSet plot using permutation-based statistical thresholds

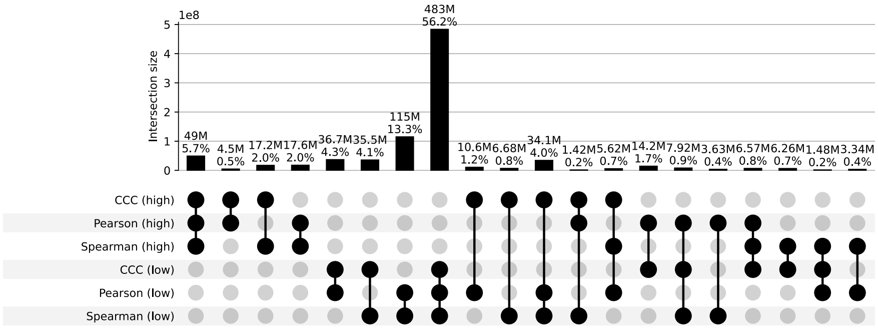

Figure S12: Distribution and UpSet plots for GTEx v8 bladder.

Brain Amygdala

a) Correlation coefficient distributions between gene pairs within GTEx v8 Brain Amygdala

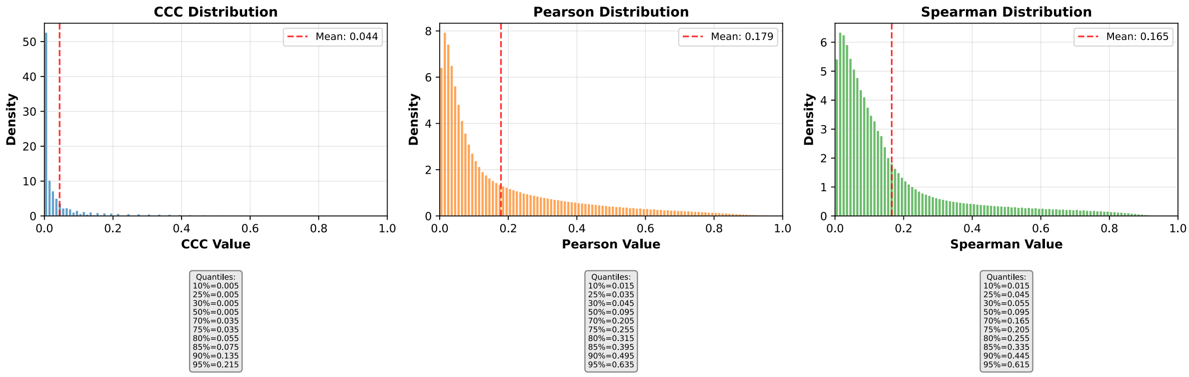

b) Corresponding cumulative histogram

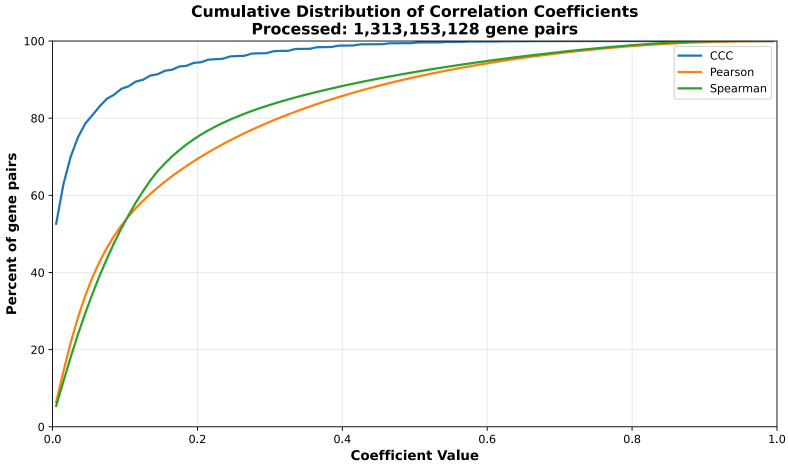

c) UpSet plot using top and bottom 30% correlations

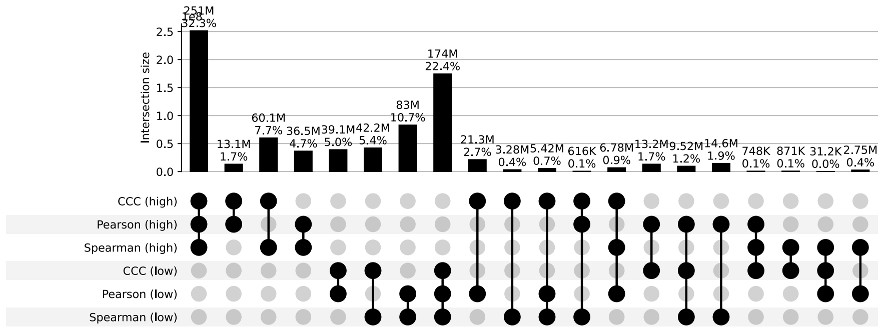

d) UpSet plot using permutation-based statistical thresholds

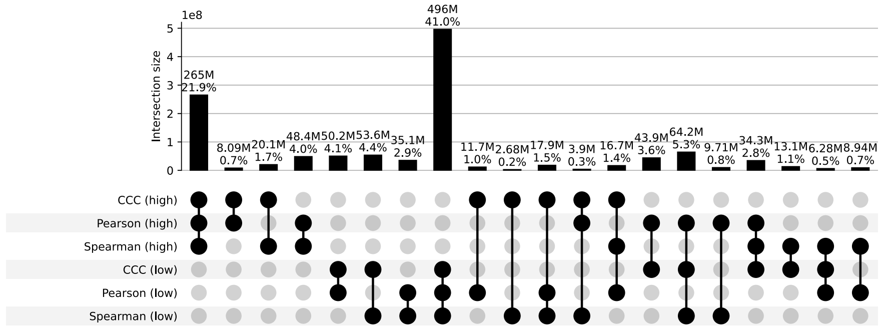

Figure S13: Distribution and UpSet plots for GTEx v8 brain amygdala.

Brain Anterior Cingulate Cortex Ba24

a) Correlation coefficient distributions between gene pairs within GTEx v8 Brain Anterior Cingulate Cortex Ba24

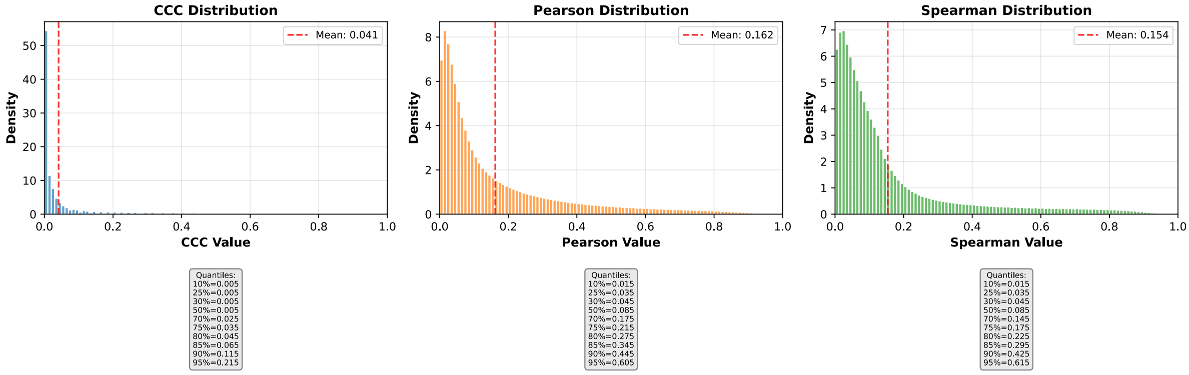

b) Corresponding cumulative histogram

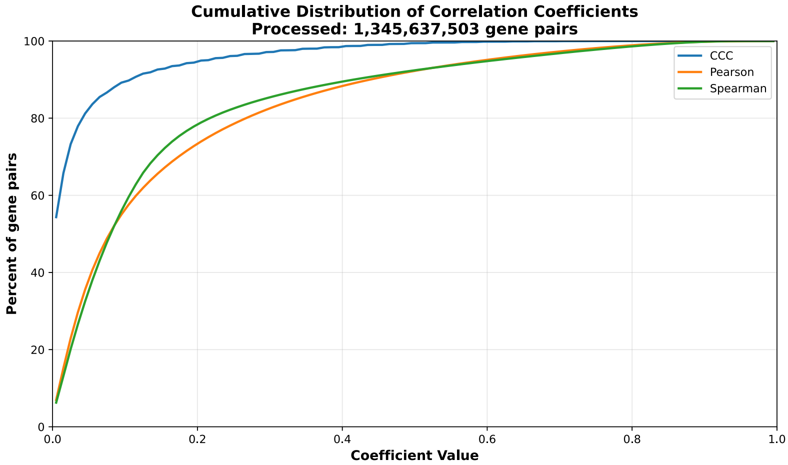

c) UpSet plot using top and bottom 30% correlations

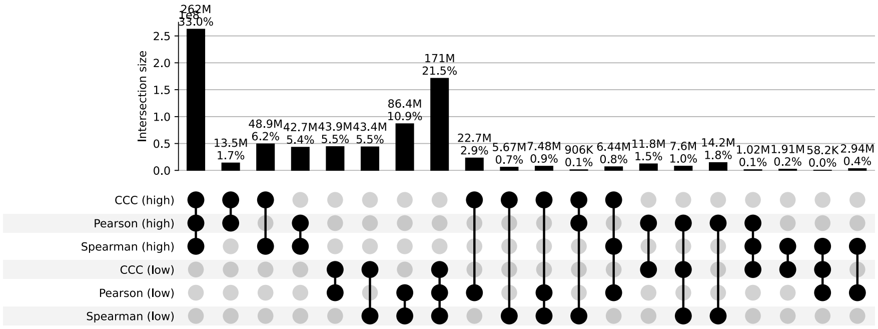

d) UpSet plot using permutation-based statistical thresholds

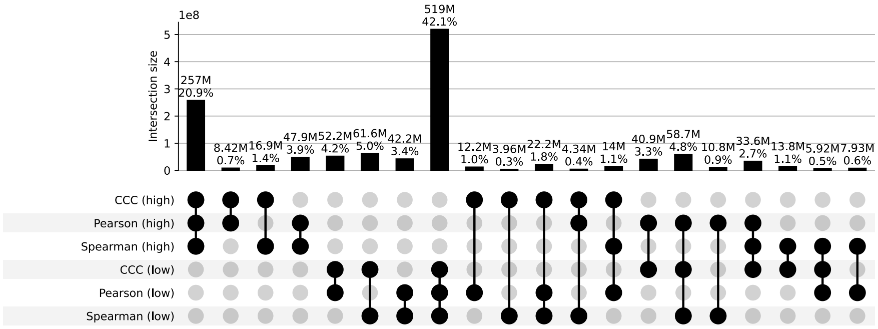

Figure S14: Distribution and UpSet plots for GTEx v8 brain anterior cingulate cortex BA24.

Brain Caudate Basal Ganglia

a) Correlation coefficient distributions between gene pairs within GTEx v8 Brain Caudate Basal Ganglia

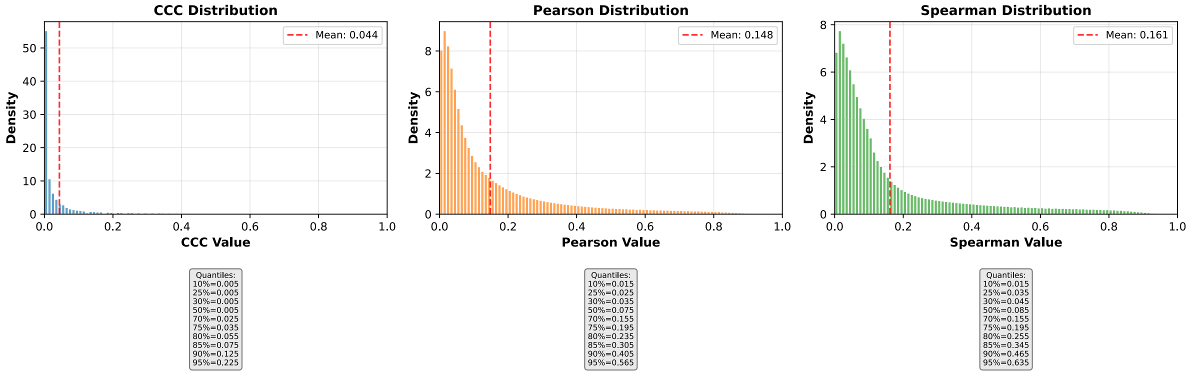

b) Corresponding cumulative histogram

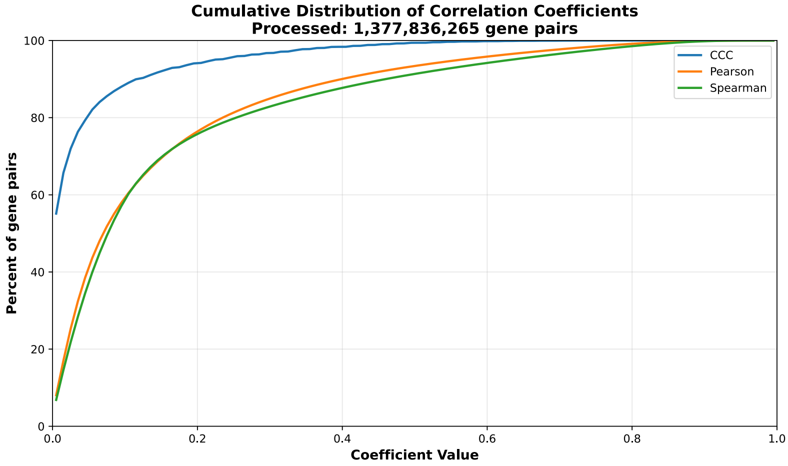

c) UpSet plot using top and bottom 30% correlations

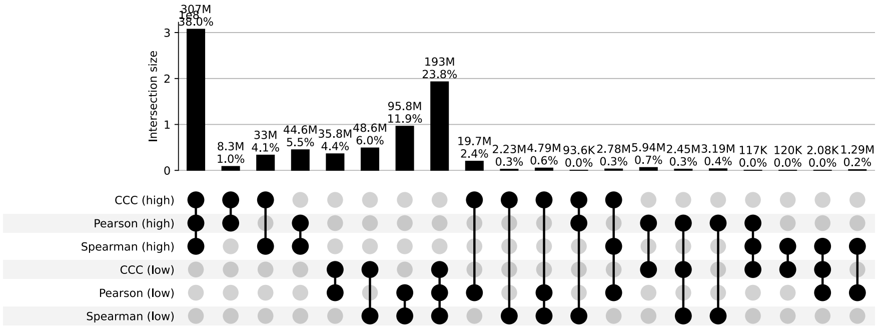

d) UpSet plot using permutation-based statistical thresholds

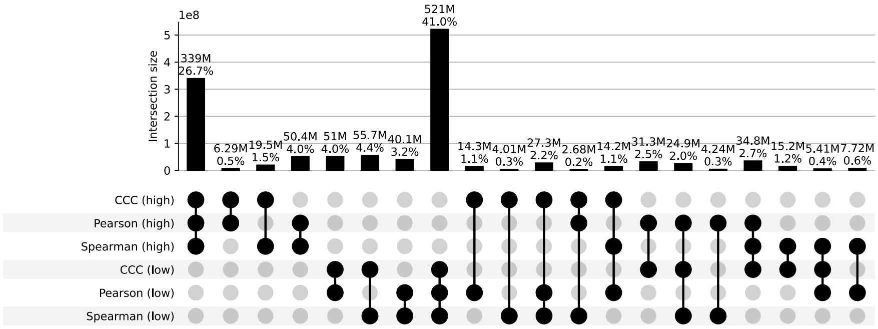

Figure S15: Distribution and UpSet plots for GTEx v8 brain caudate basal ganglia.

Brain Cerebellar Hemisphere

a) Correlation coefficient distributions between gene pairs within GTEx v8 Brain Cerebellar Hemisphere

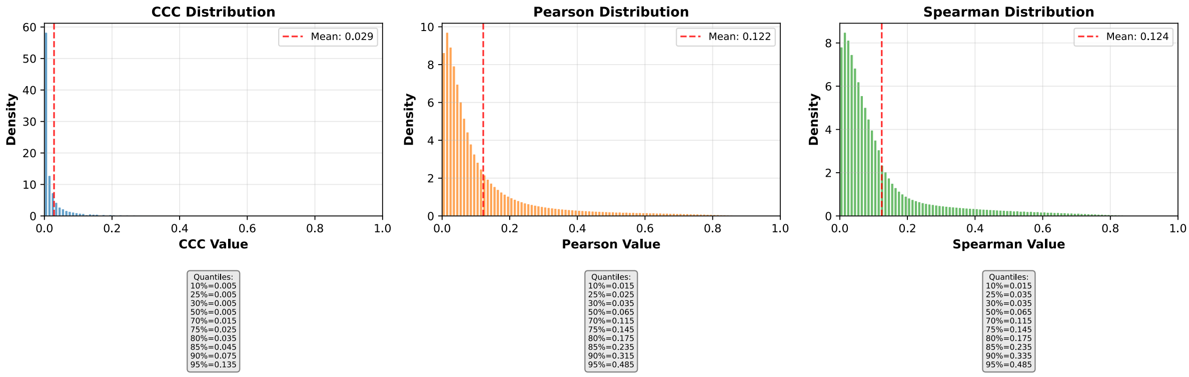

b) Corresponding cumulative histogram

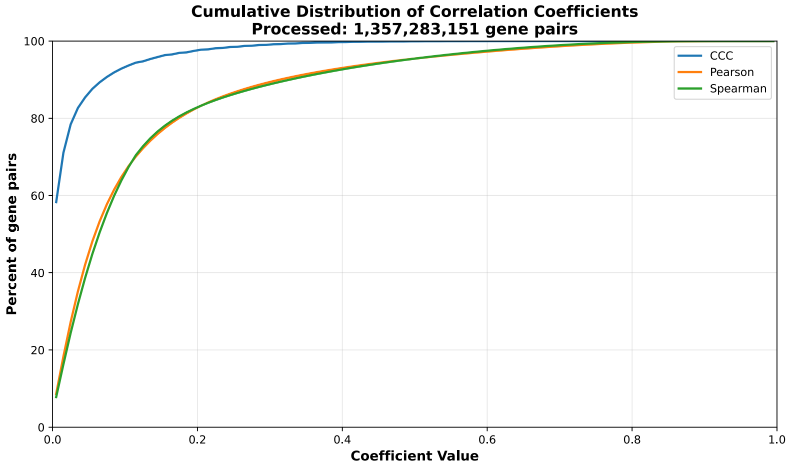

c) UpSet plot using top and bottom 30% correlations

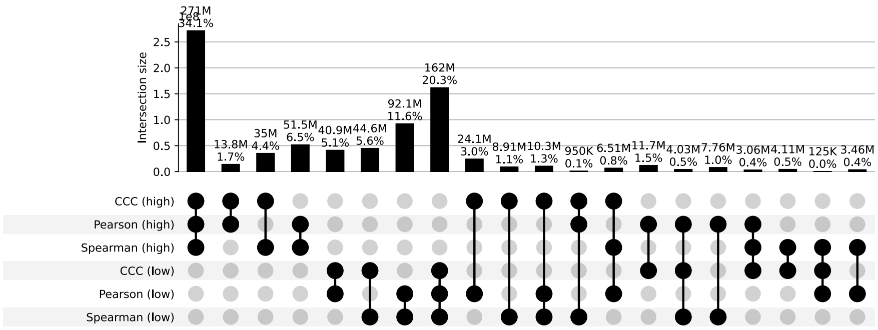

d) UpSet plot using permutation-based statistical thresholds

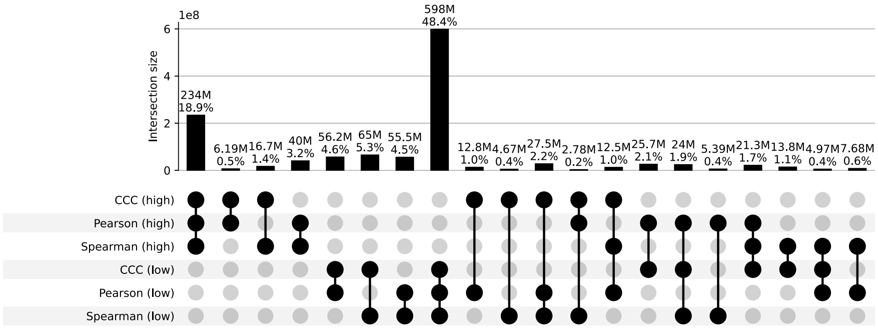

Figure S16: Distribution and UpSet plots for GTEx v8 brain cerebellar hemisphere.

Brain Cerebellum

a) Correlation coefficient distributions between gene pairs within GTEx v8 Brain Cerebellum

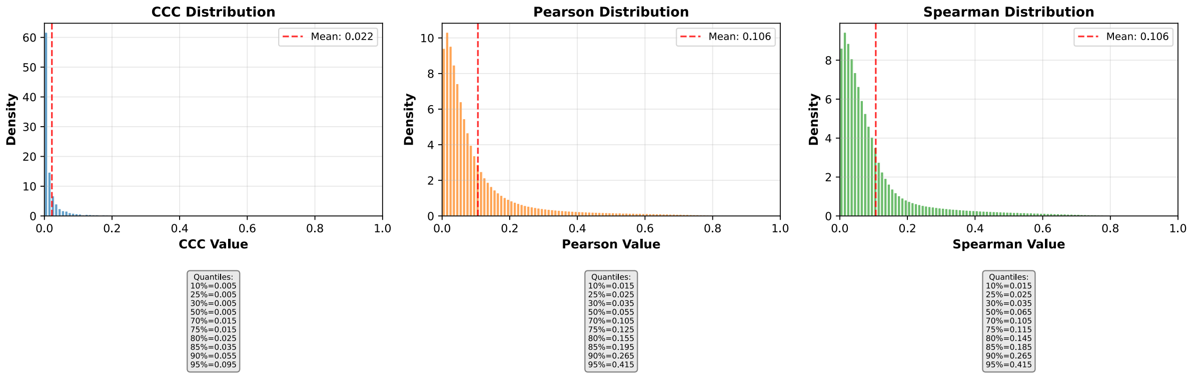

b) Corresponding cumulative histogram

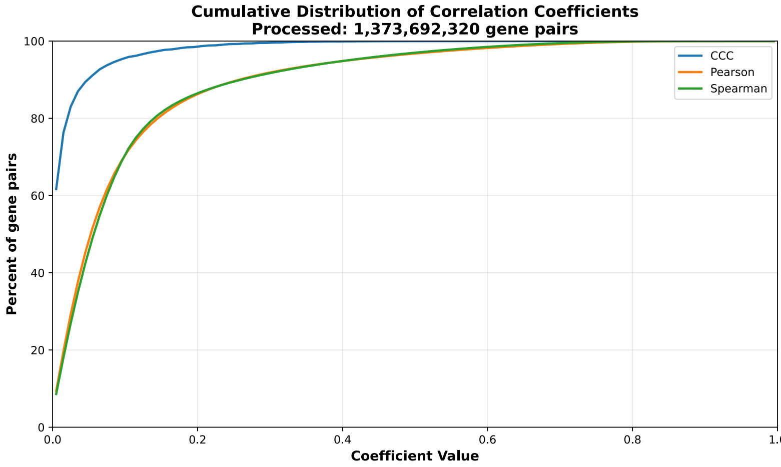

c) UpSet plot using top and bottom 30% correlations

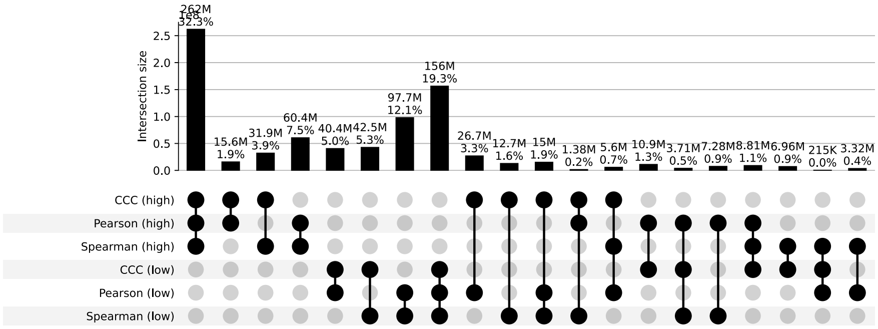

d) UpSet plot using permutation-based statistical thresholds

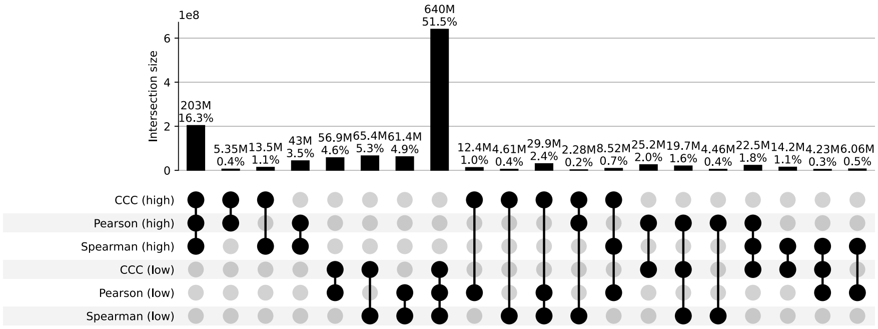

Figure S17: Distribution and UpSet plots for GTEx v8 brain cerebellum.

Brain Cortex

a) Correlation coefficient distributions between gene pairs within GTEx v8 Brain Cortex

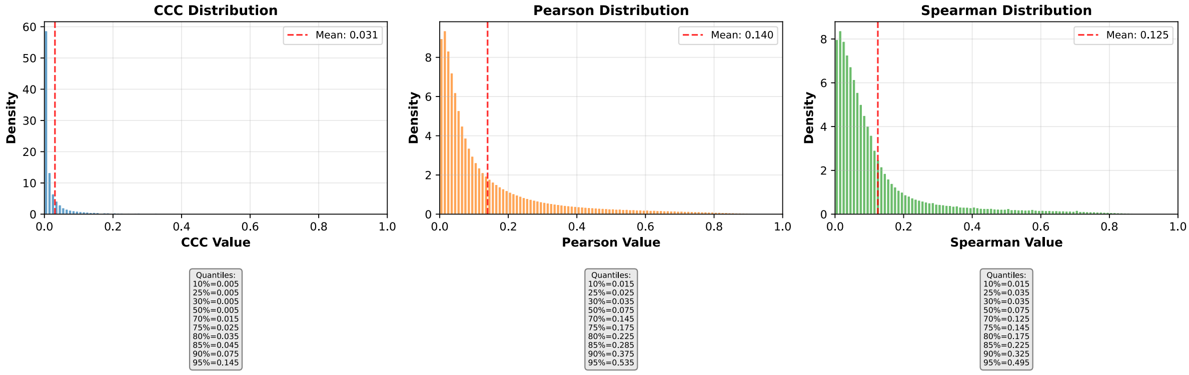

b) Corresponding cumulative histogram

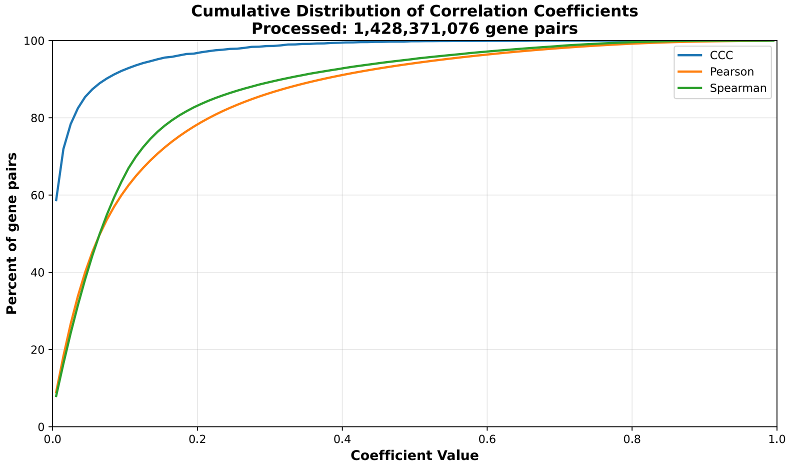

c) UpSet plot using top and bottom 30% correlations

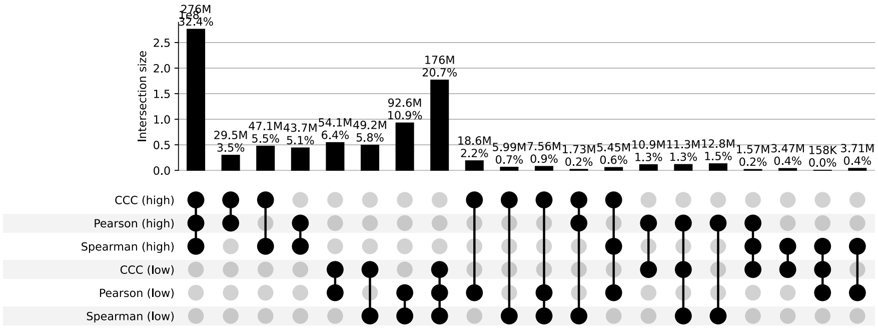

d) UpSet plot using permutation-based statistical thresholds

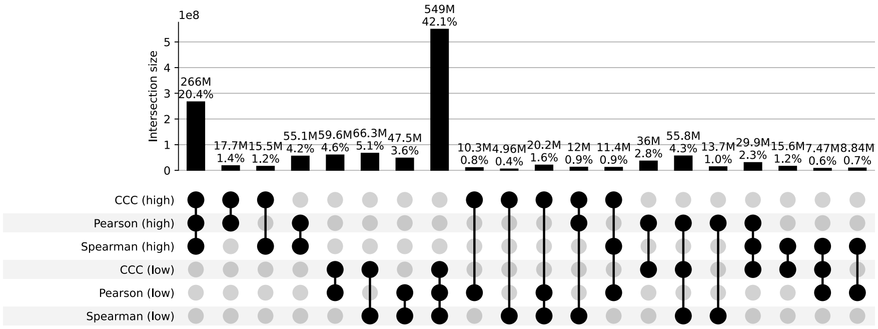

Figure S18: Distribution and UpSet plots for GTEx v8 brain cortex.

Brain Frontal Cortex Ba9

a) Correlation coefficient distributions between gene pairs within GTEx v8 Brain Frontal Cortex Ba9

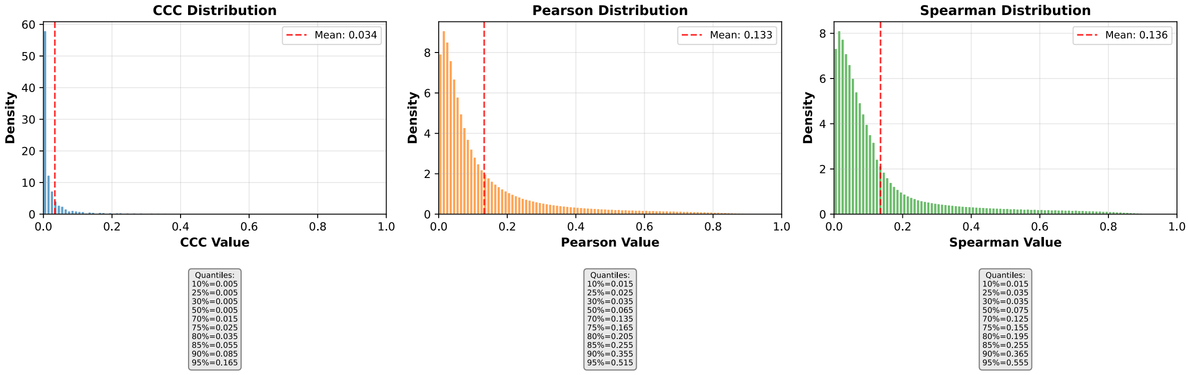

b) Corresponding cumulative histogram

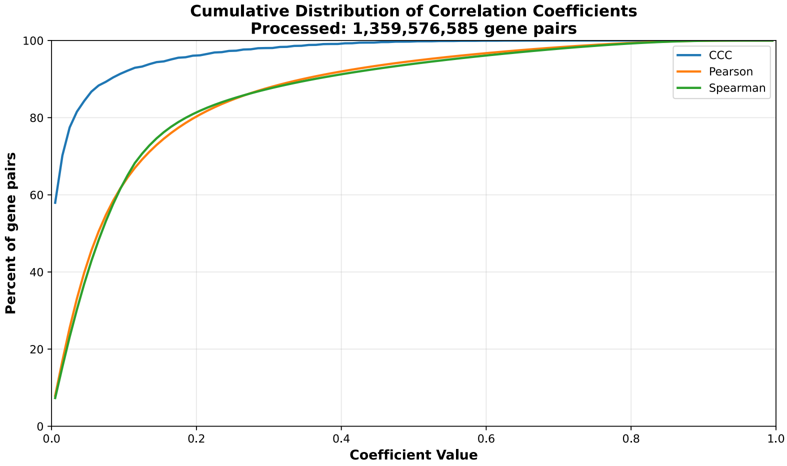

c) UpSet plot using top and bottom 30% correlations

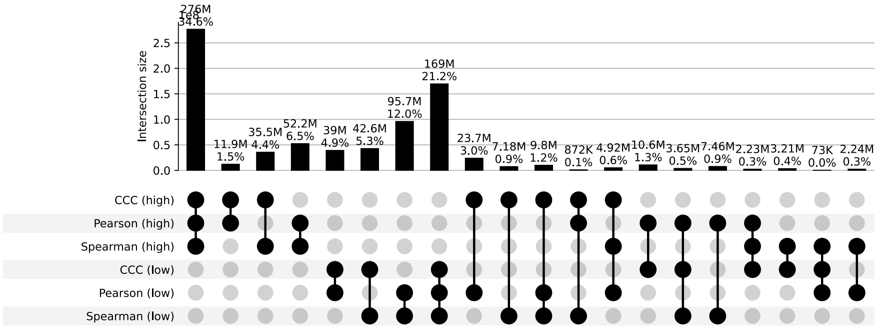

d) UpSet plot using permutation-based statistical thresholds

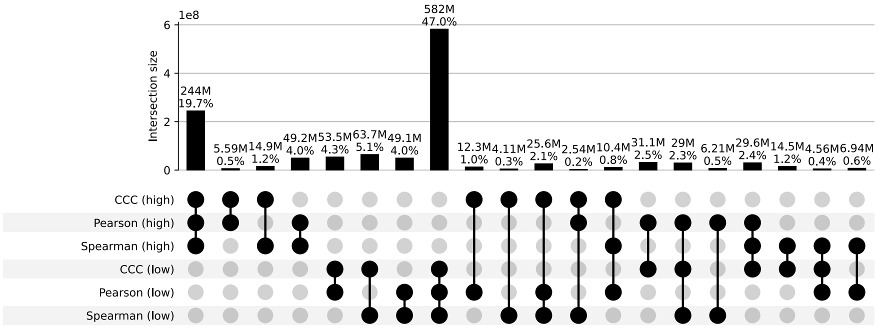

Figure S19: Distribution and UpSet plots for GTEx v8 brain frontal cortex BA9.

Brain Hippocampus

a) Correlation coefficient distributions between gene pairs within GTEx v8 Brain Hippocampus

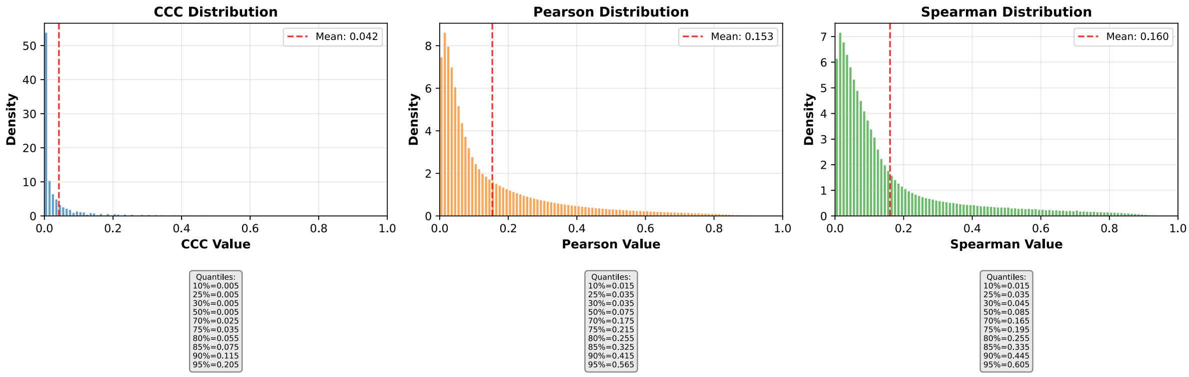

b) Corresponding cumulative histogram

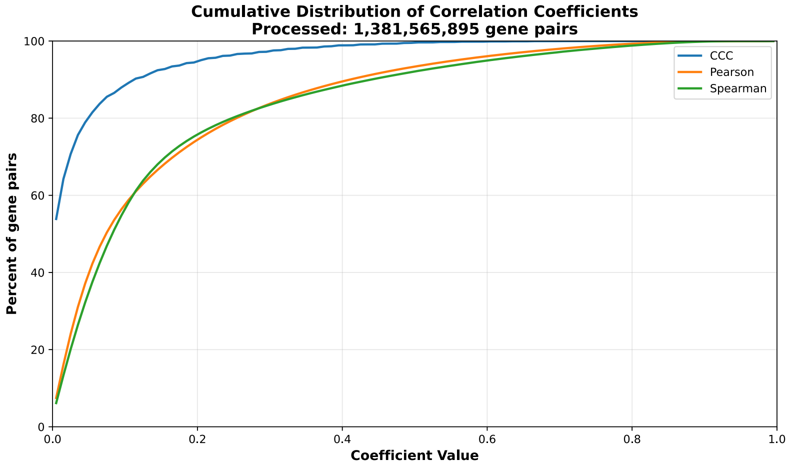

c) UpSet plot using top and bottom 30% correlations

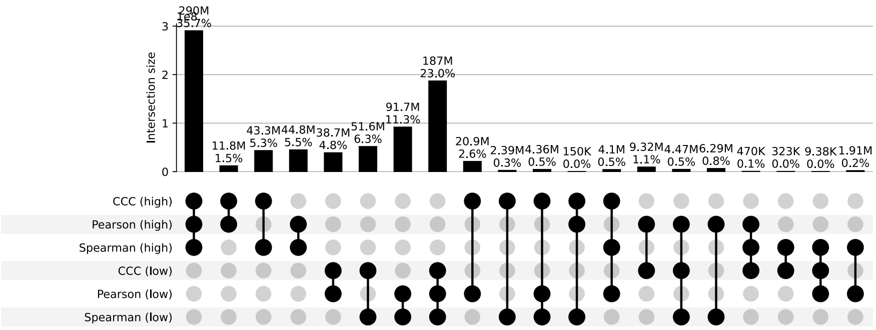

d) UpSet plot using permutation-based statistical thresholds

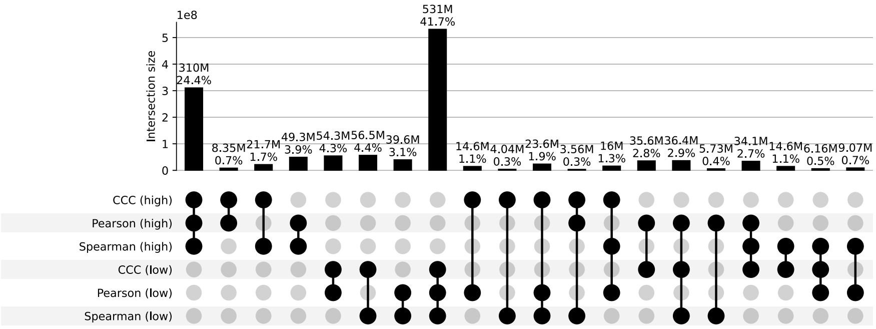

Figure S20: Distribution and UpSet plots for GTEx v8 brain hippocampus.

Brain Hypothalamus

a) Correlation coefficient distributions between gene pairs within GTEx v8 Brain Hypothalamus

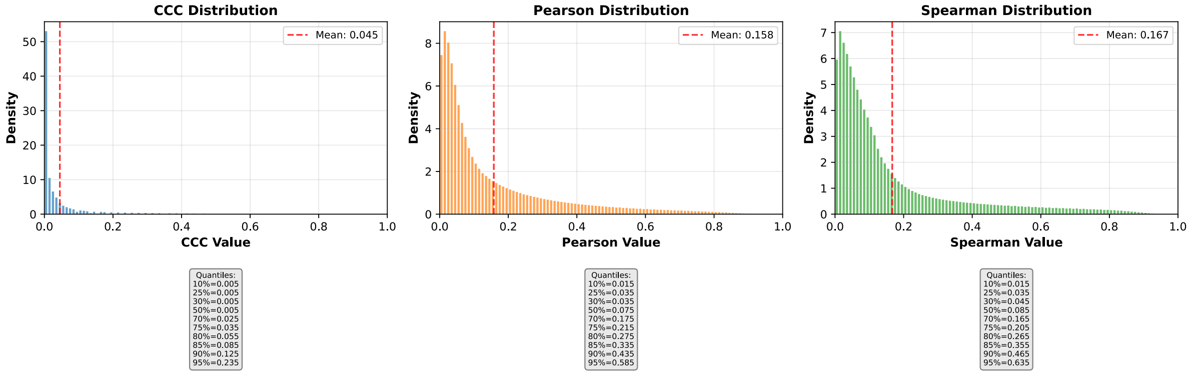

b) Corresponding cumulative histogram

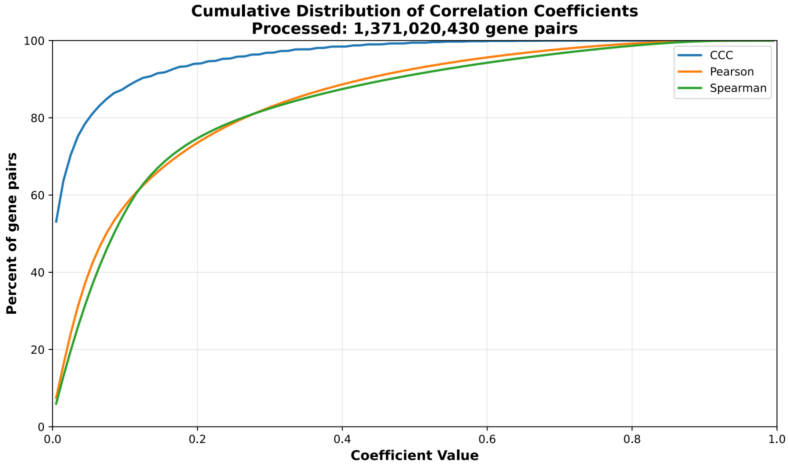

c) UpSet plot using top and bottom 30% correlations

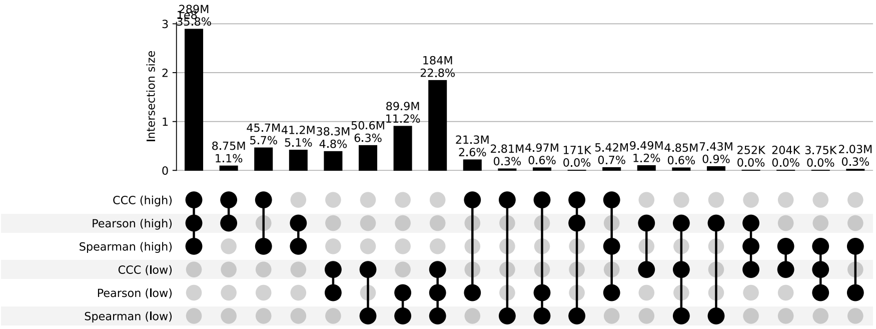

d) UpSet plot using permutation-based statistical thresholds

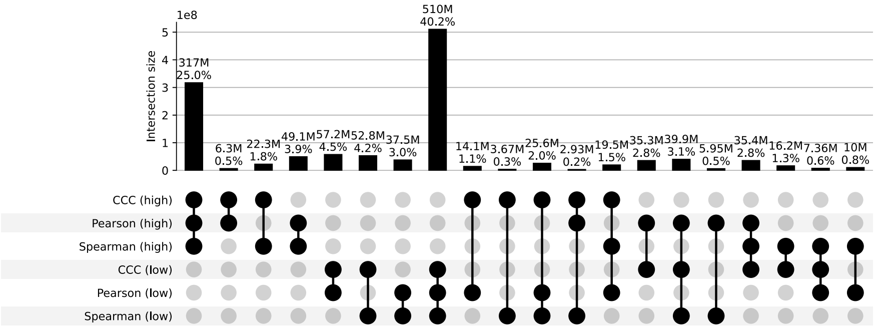

Figure S21: Distribution and UpSet plots for GTEx v8 brain hypothalamus.

Brain Nucleus Accumbens Basal Ganglia

a) Correlation coefficient distributions between gene pairs within GTEx v8 Brain Nucleus Accumbens Basal Ganglia

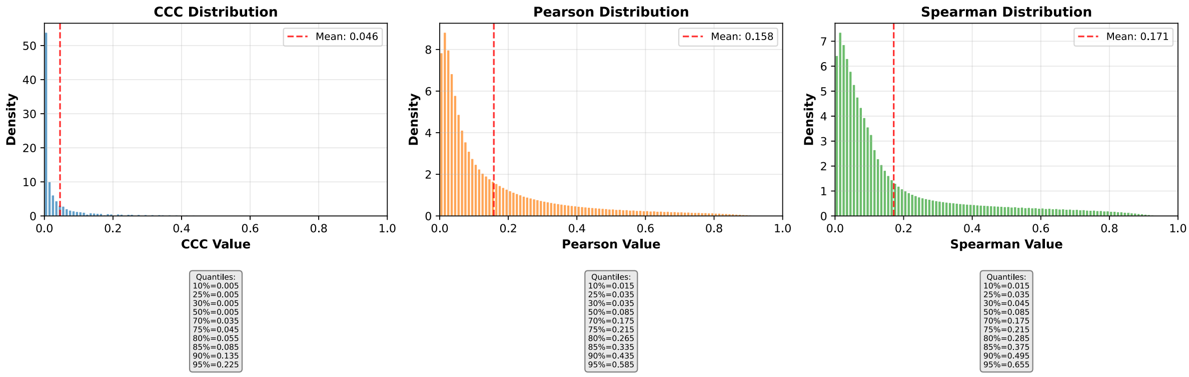

b) Corresponding cumulative histogram

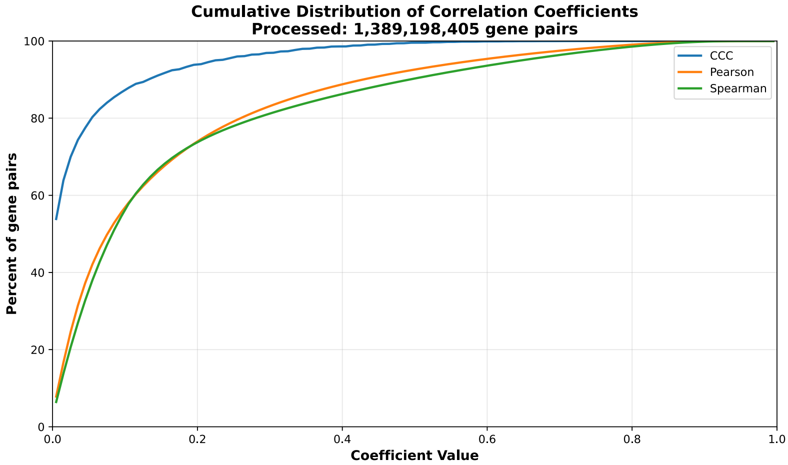

c) UpSet plot using top and bottom 30% correlations

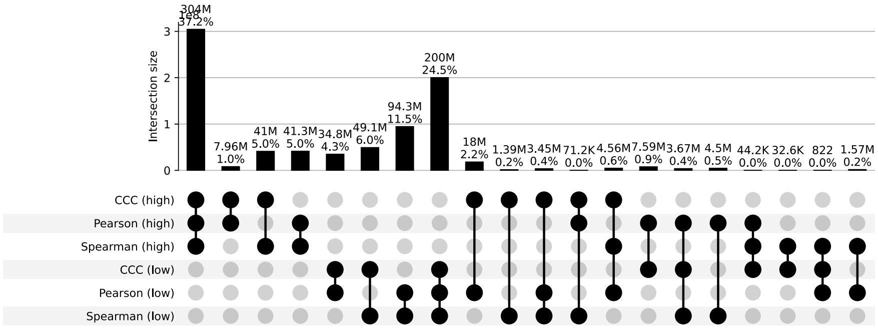

d) UpSet plot using permutation-based statistical thresholds

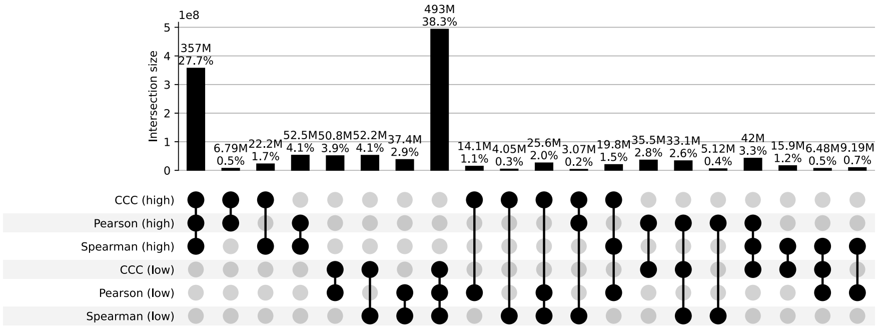

Figure S22: Distribution and UpSet plots for GTEx v8 brain nucleus accumbens basal ganglia.

Brain Putamen Basal Ganglia

a) Correlation coefficient distributions between gene pairs within GTEx v8 Brain Putamen Basal Ganglia

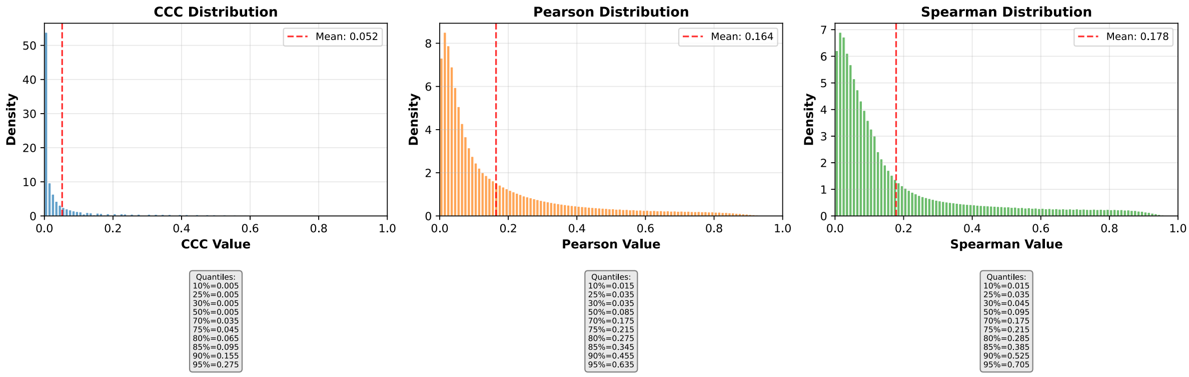

b) Corresponding cumulative histogram

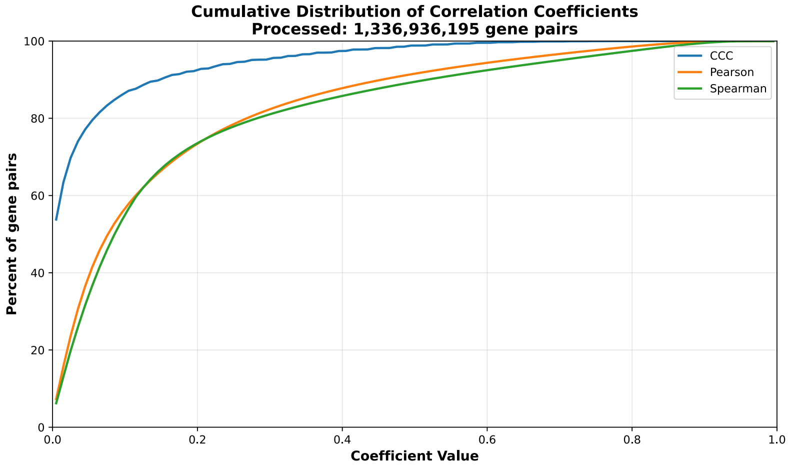

c) UpSet plot using top and bottom 30% correlations

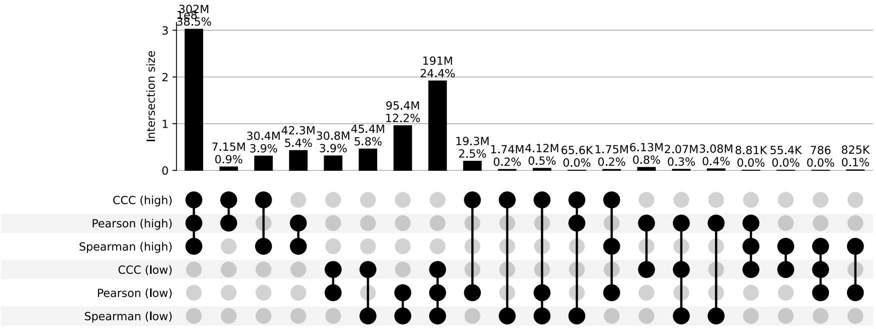

d) UpSet plot using permutation-based statistical thresholds

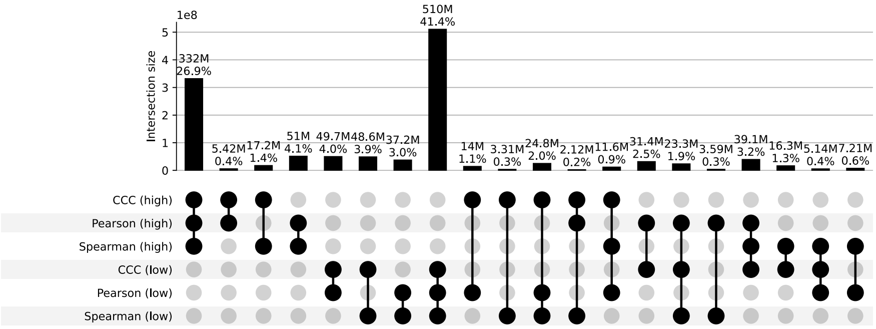

Figure S23: Distribution and UpSet plots for GTEx v8 brain putamen basal ganglia.

Brain Spinal Cord Cervical C1

a) Correlation coefficient distributions between gene pairs within GTEx v8 Brain Spinal Cord Cervical C1

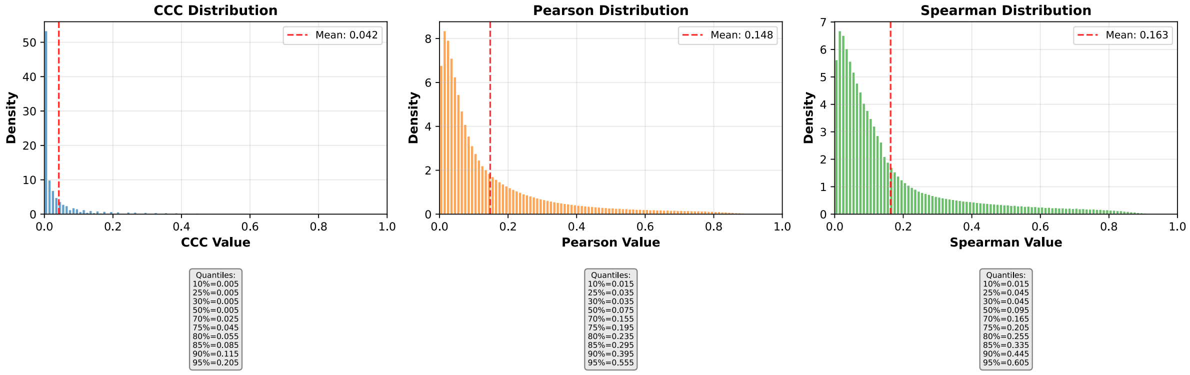

b) Corresponding cumulative histogram

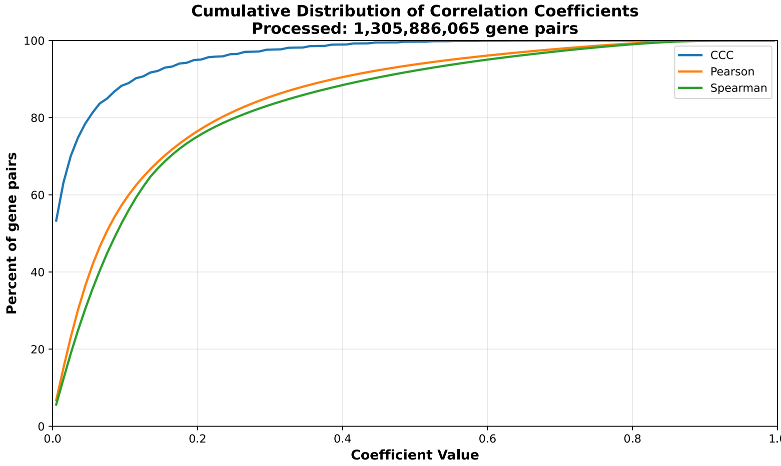

c) UpSet plot using top and bottom 30% correlations

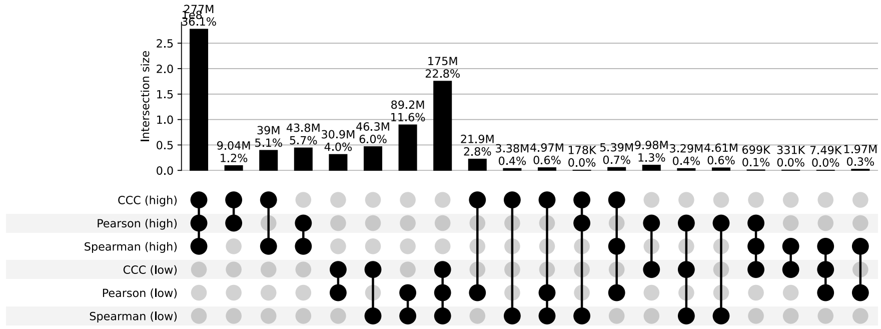

d) UpSet plot using permutation-based statistical thresholds

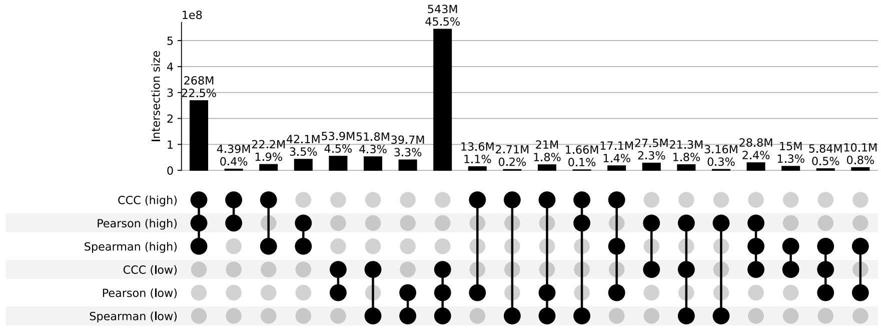

Figure S24: Distribution and UpSet plots for GTEx v8 brain spinal cord cervical C1.

Brain Substantia Nigra

a) Correlation coefficient distributions between gene pairs within GTEx v8 Brain Substantia Nigra

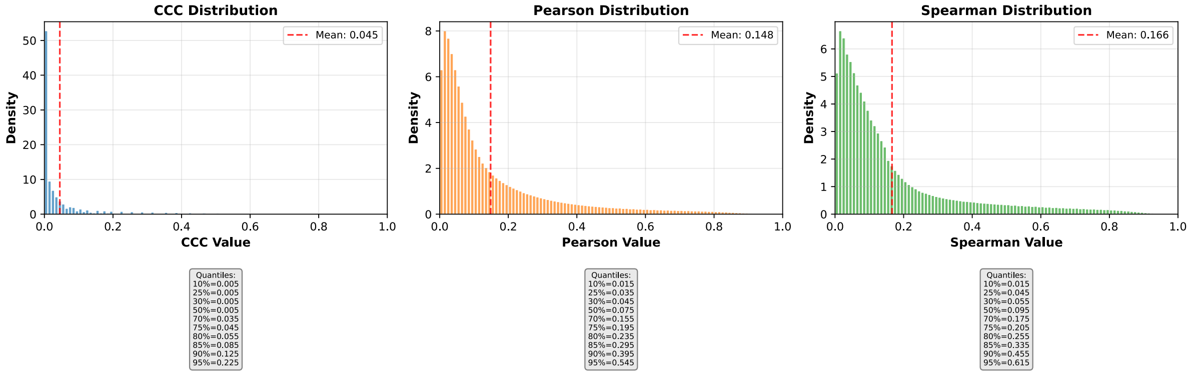

b) Corresponding cumulative histogram

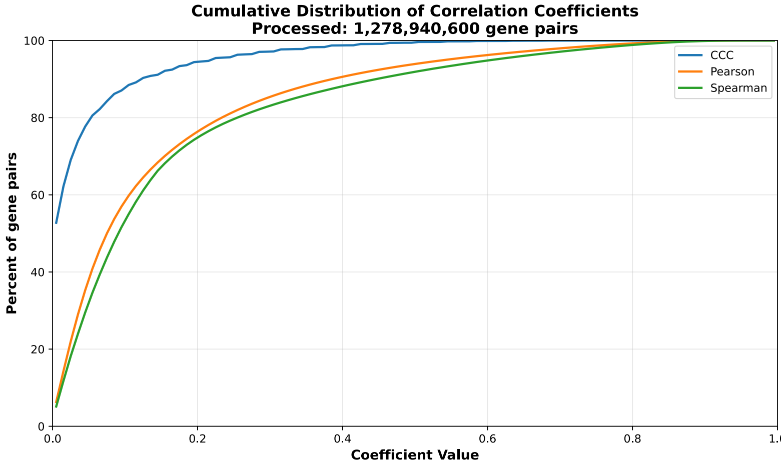

c) UpSet plot using top and bottom 30% correlations

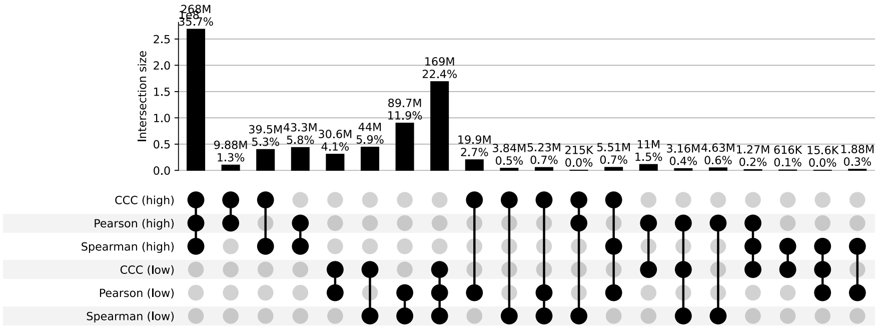

d) UpSet plot using permutation-based statistical thresholds

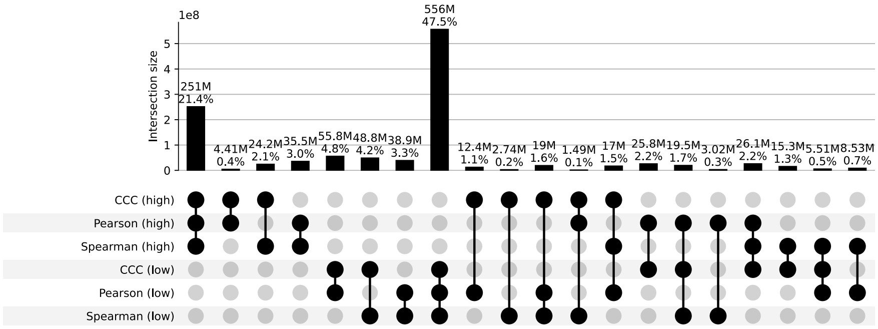

Figure S25: Distribution and UpSet plots for GTEx v8 brain substantia nigra.

Breast Mammary Tissue

a) Correlation coefficient distributions between gene pairs within GTEx v8 Breast Mammary Tissue

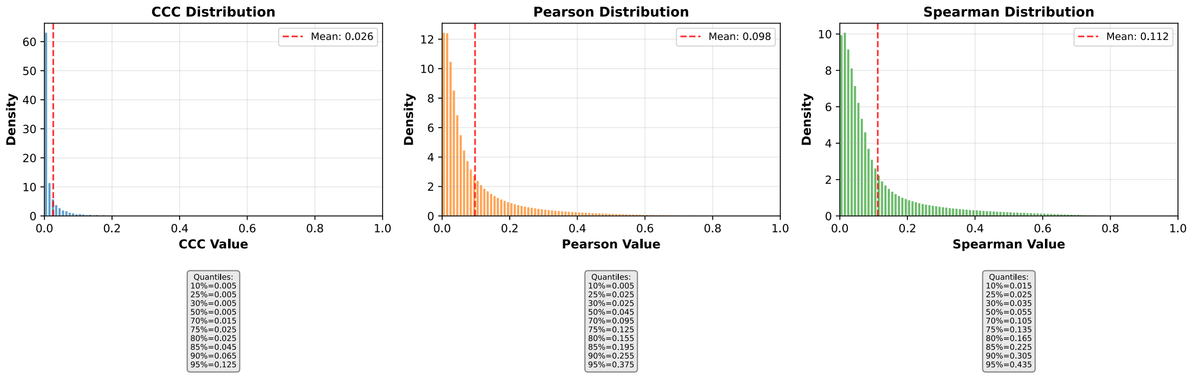

b) Corresponding cumulative histogram

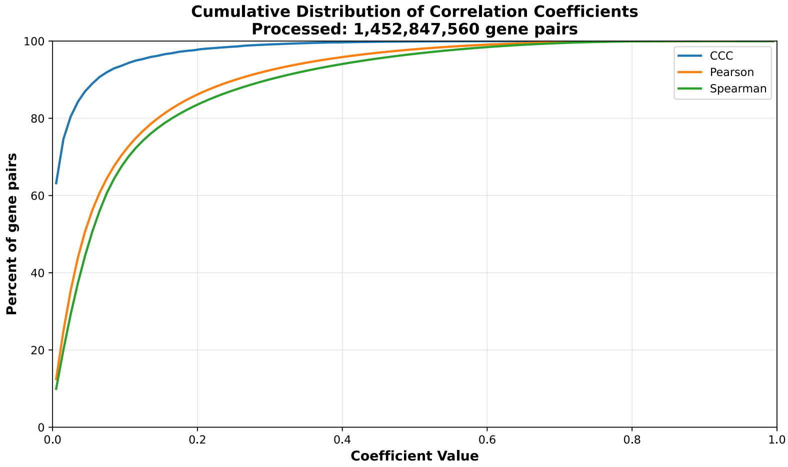

c) UpSet plot using top and bottom 30% correlations

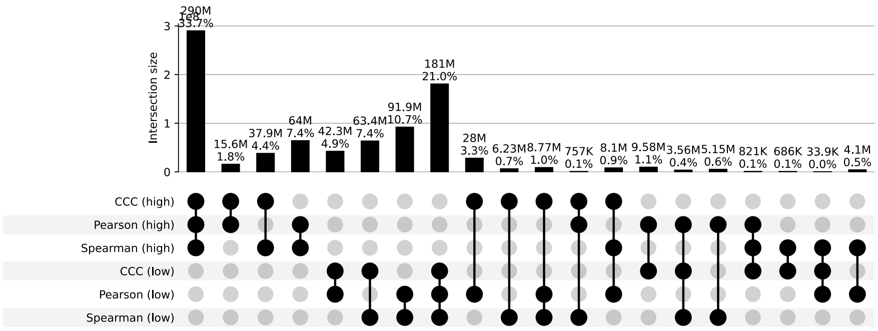

d) UpSet plot using permutation-based statistical thresholds

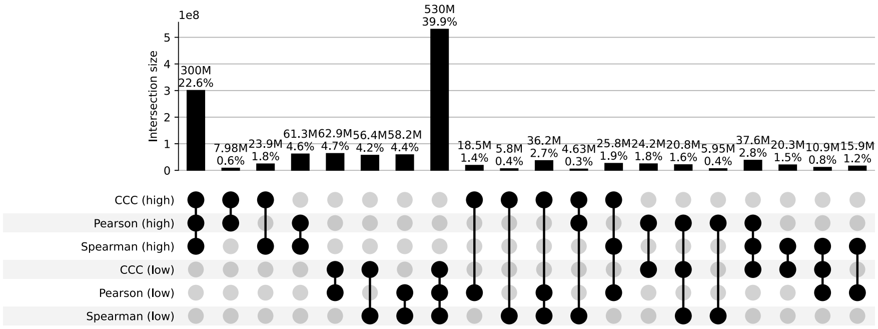

Figure S26: Distribution and UpSet plots for GTEx v8 breast mammary tissue.

Cells Cultured Fibroblasts

a) Correlation coefficient distributions between gene pairs within GTEx v8 Cells Cultured Fibroblasts

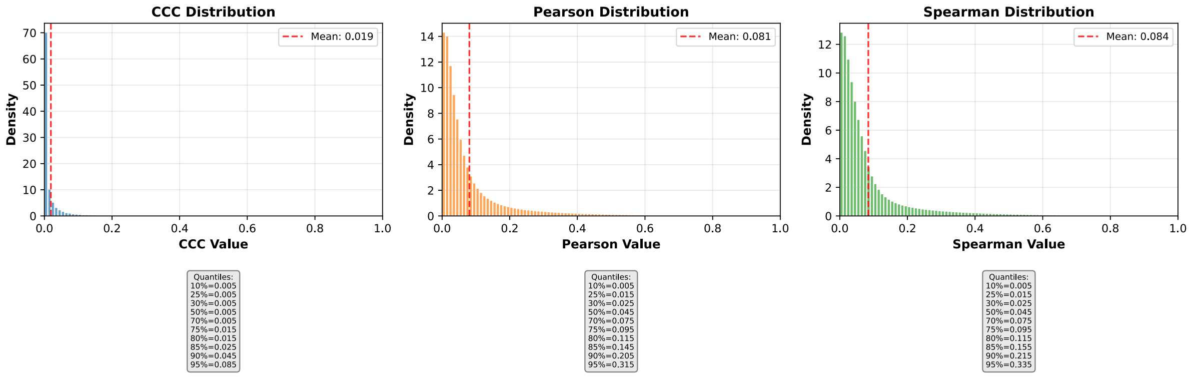

b) Corresponding cumulative histogram

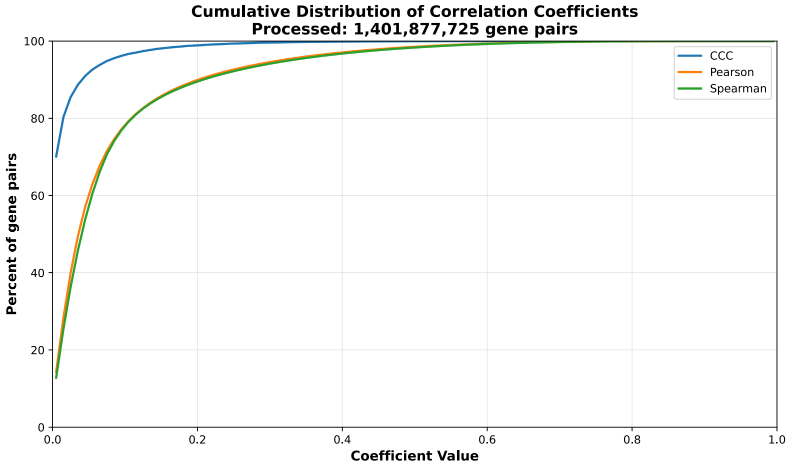

c) UpSet plot using top and bottom 30% correlations

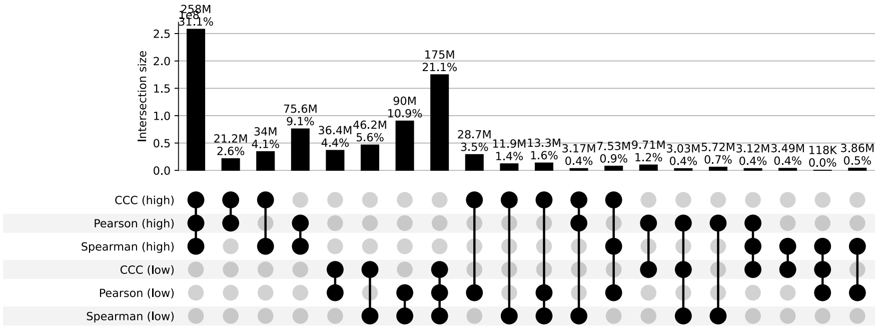

d) UpSet plot using permutation-based statistical thresholds

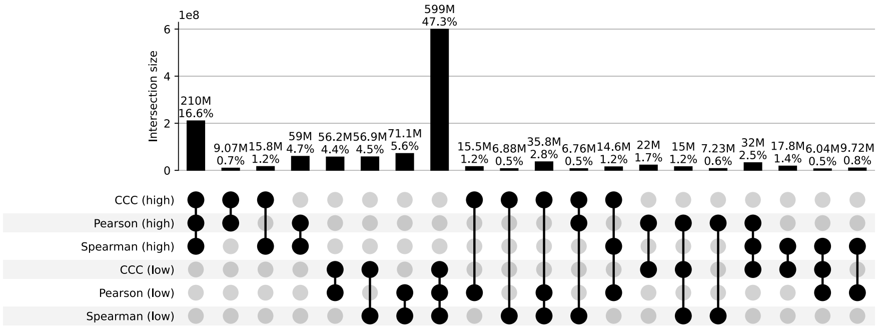

Figure S27: Distribution and UpSet plots for GTEx v8 cells cultured fibroblasts.

Cells Ebvtransformed Lymphocytes

a) Correlation coefficient distributions between gene pairs within GTEx v8 Cells Ebvtransformed Lymphocytes

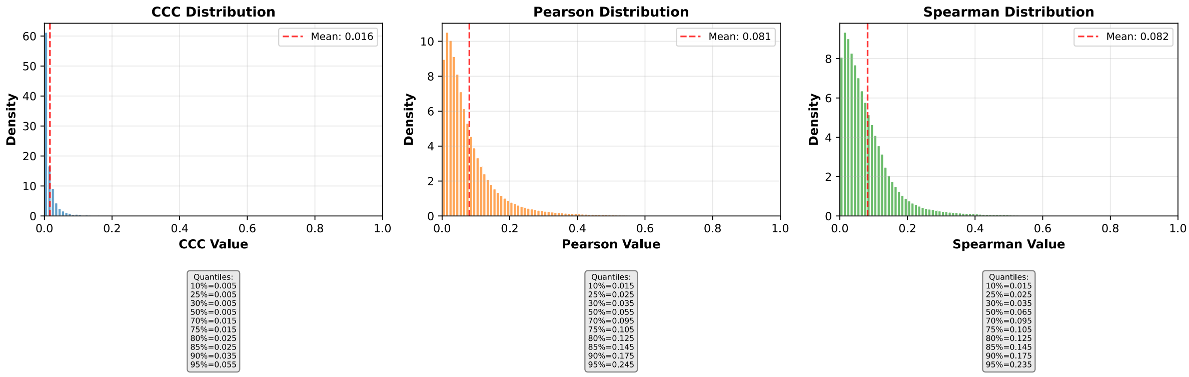

b) Corresponding cumulative histogram

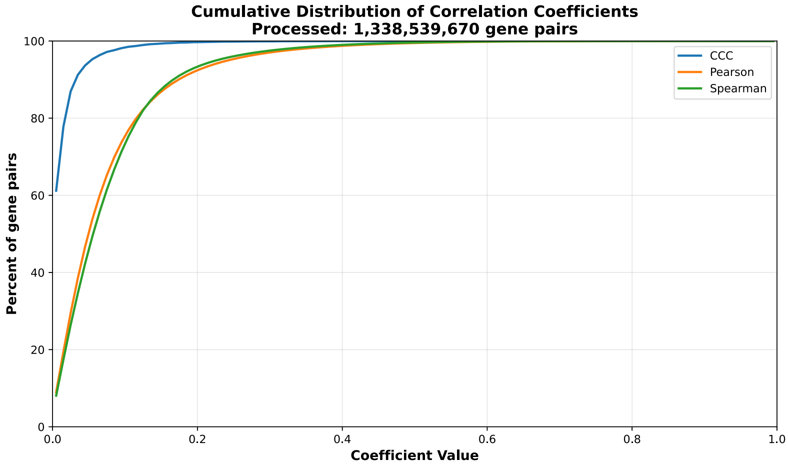

c) UpSet plot using top and bottom 30% correlations

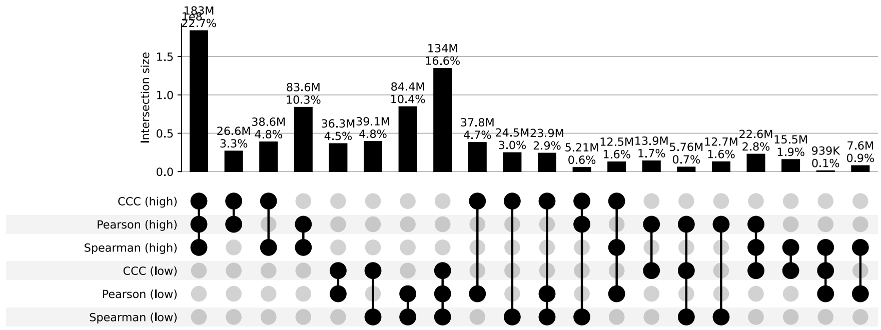

d) UpSet plot using permutation-based statistical thresholds

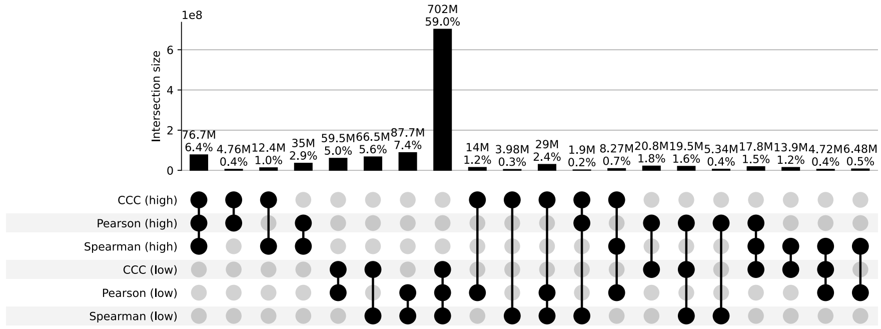

Figure S28: Distribution and UpSet plots for GTEx v8 cells EBV-transformed lymphocytes.

Cervix Ectocervix

a) Correlation coefficient distributions between gene pairs within GTEx v8 Cervix Ectocervix

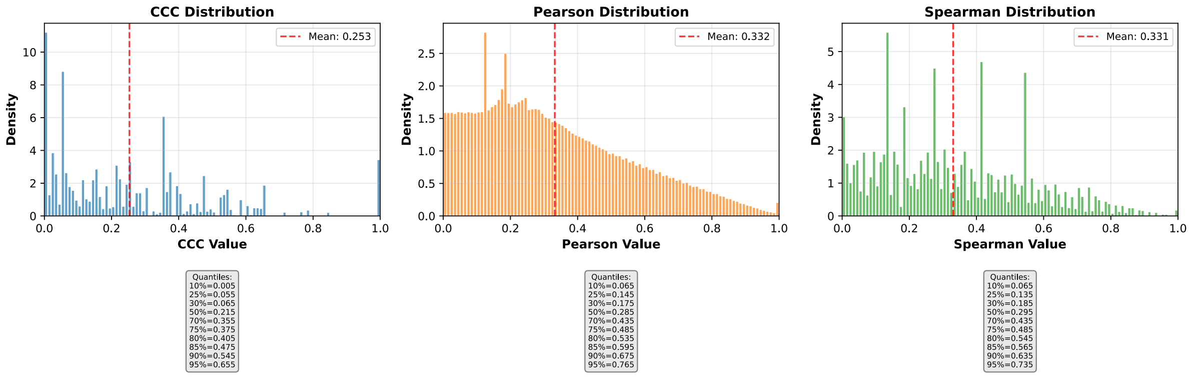

b) Corresponding cumulative histogram

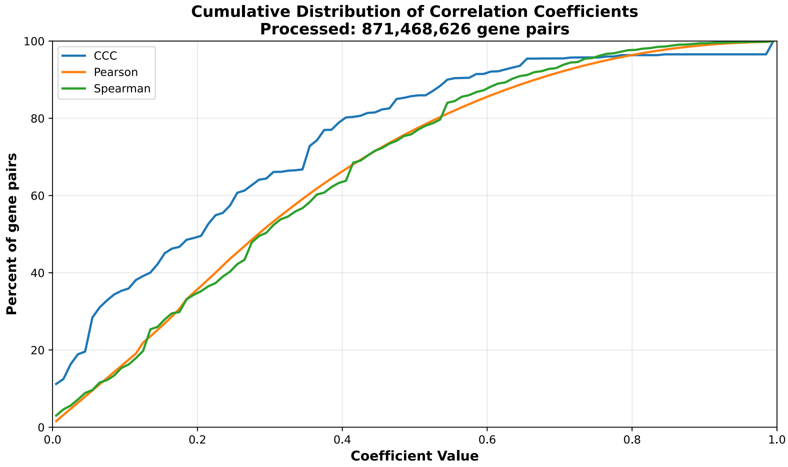

c) UpSet plot using top and bottom 30% correlations

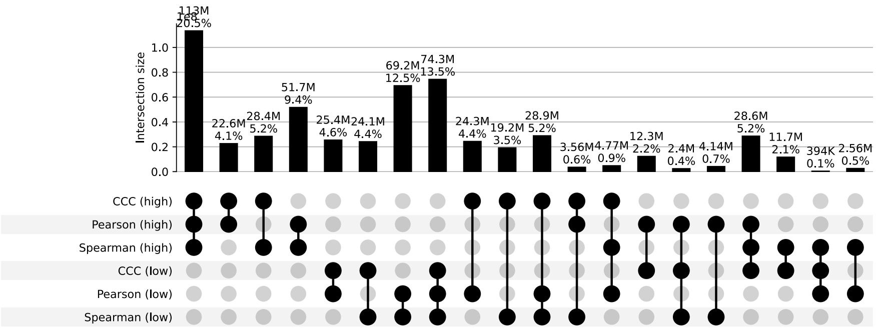

d) UpSet plot using permutation-based statistical thresholds

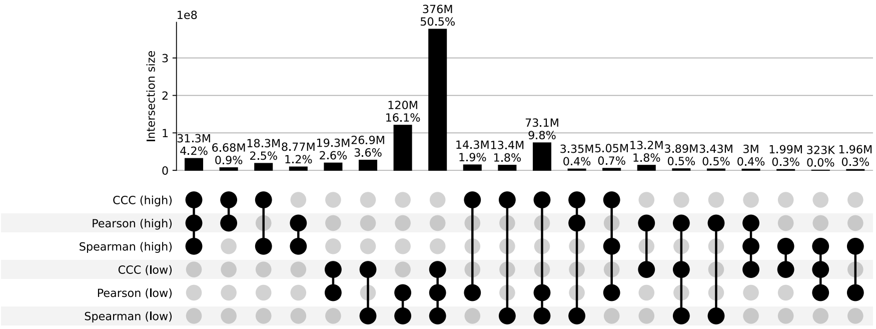

Figure S29: Distribution and UpSet plots for GTEx v8 cervix ectocervix.

Cervix Endocervix

a) Correlation coefficient distributions between gene pairs within GTEx v8 Cervix Endocervix

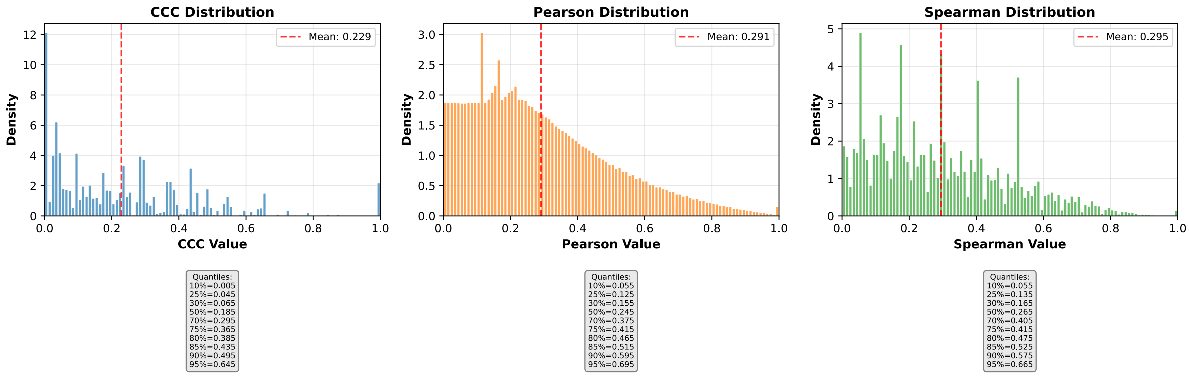

b) Corresponding cumulative histogram

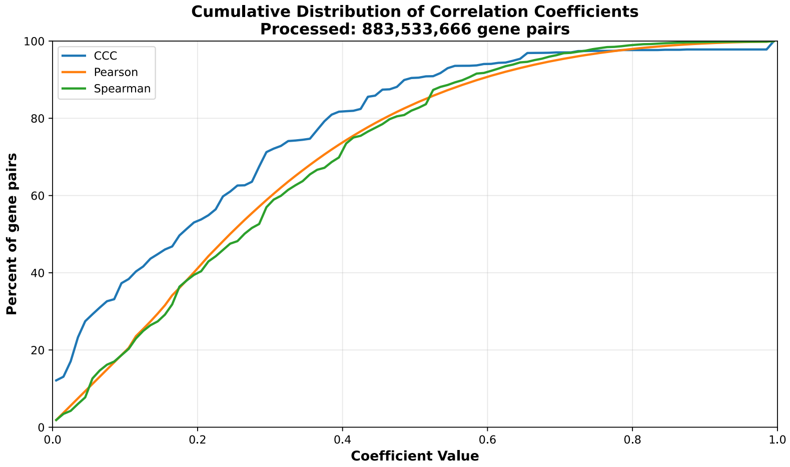

c) UpSet plot using top and bottom 30% correlations

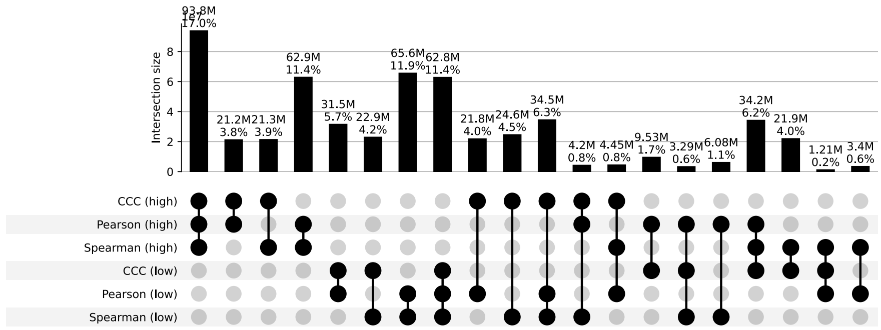

d) UpSet plot using permutation-based statistical thresholds

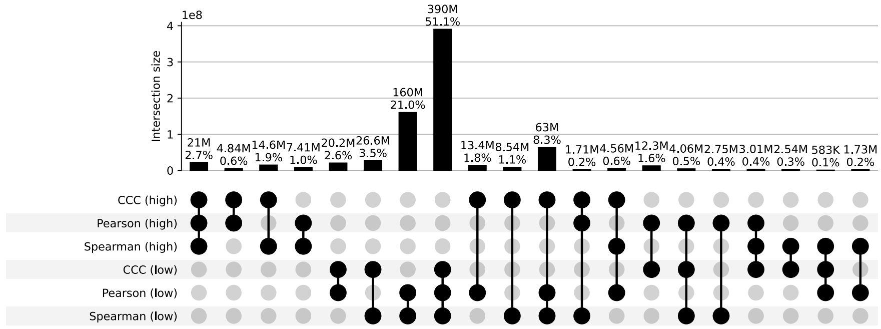

Figure S30: Distribution and UpSet plots for GTEx v8 cervix endocervix.

Colon Sigmoid

a) Correlation coefficient distributions between gene pairs within GTEx v8 Colon Sigmoid

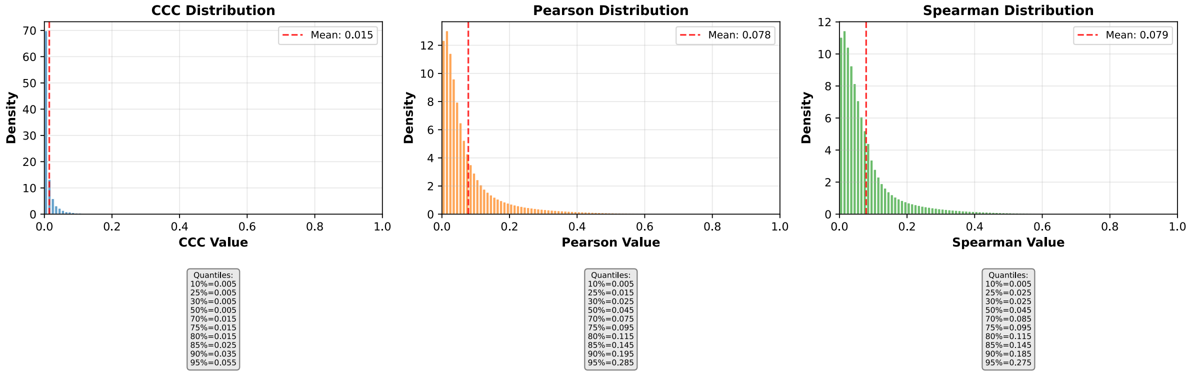

b) Corresponding cumulative histogram

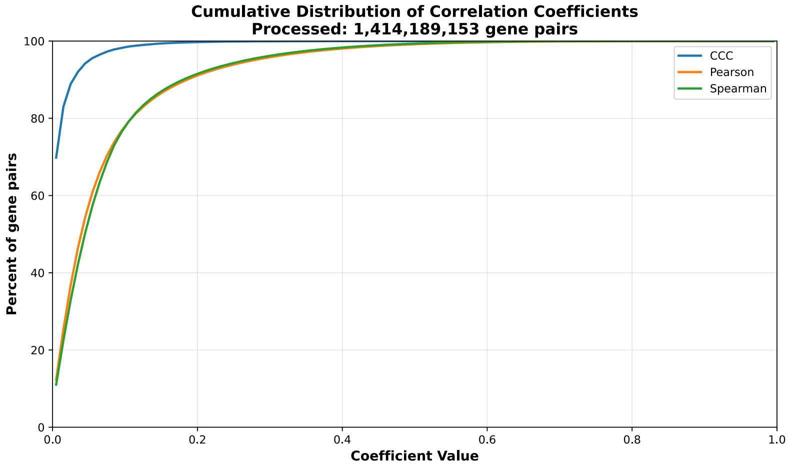

c) UpSet plot using top and bottom 30% correlations

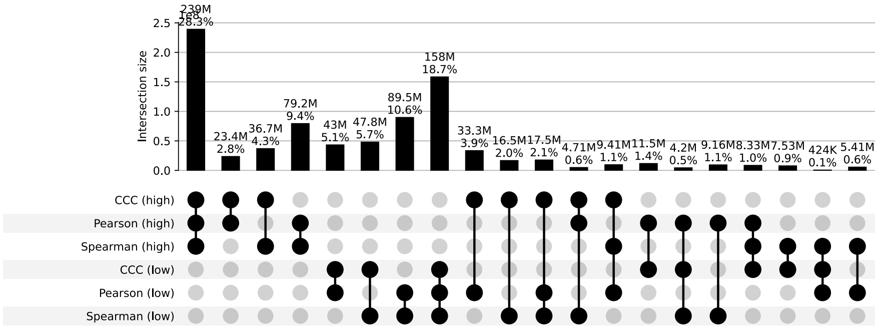

d) UpSet plot using permutation-based statistical thresholds

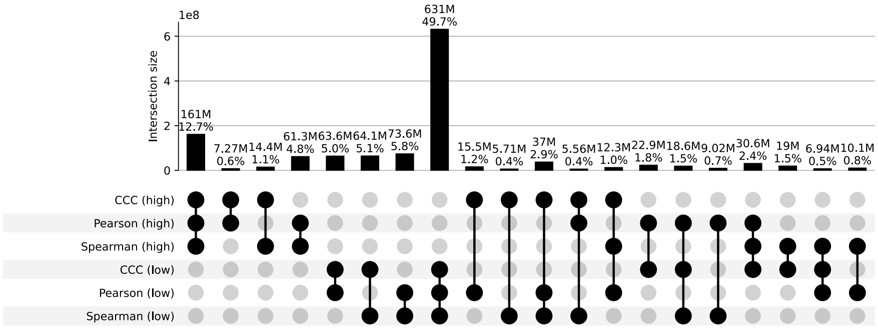

Figure S31: Distribution and UpSet plots for GTEx v8 colon sigmoid.

Colon Transverse

a) Correlation coefficient distributions between gene pairs within GTEx v8 Colon Transverse

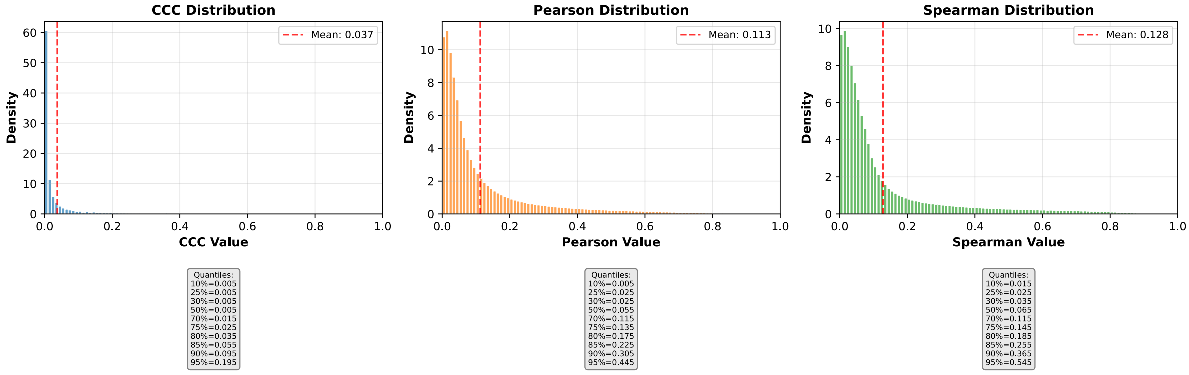

b) Corresponding cumulative histogram

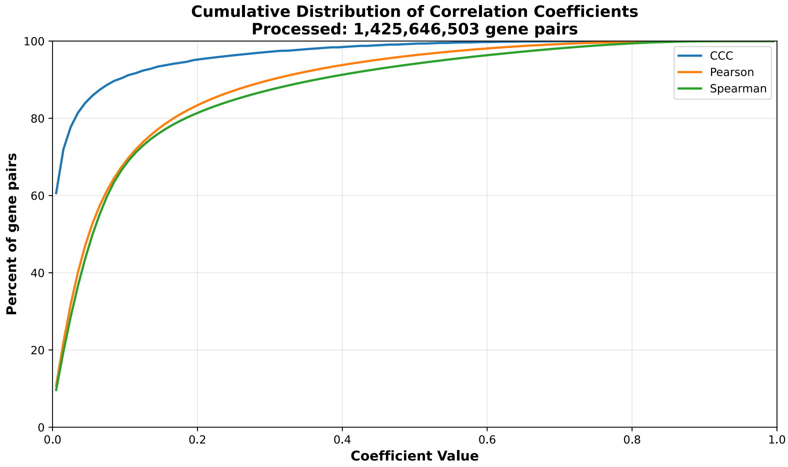

c) UpSet plot using top and bottom 30% correlations

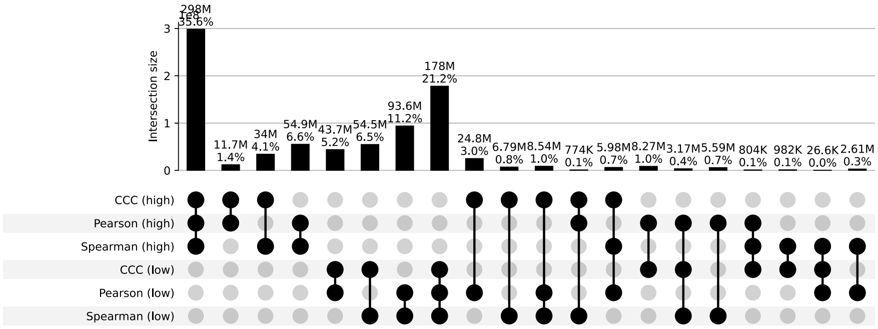

d) UpSet plot using permutation-based statistical thresholds

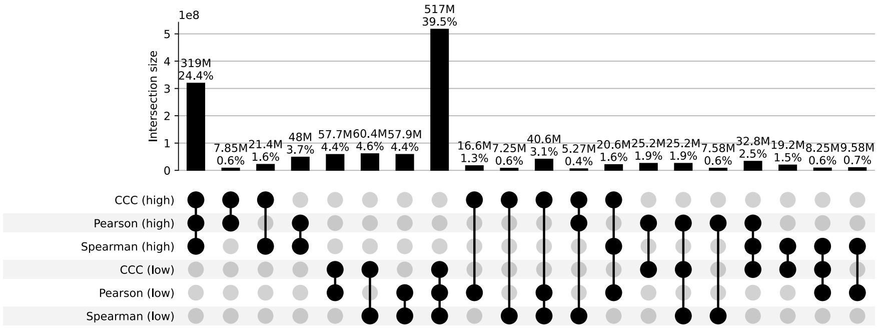

Figure S32: Distribution and UpSet plots for GTEx v8 colon transverse.

Esophagus Gastroesophageal Junction

a) Correlation coefficient distributions between gene pairs within GTEx v8 Esophagus Gastroesophageal Junction

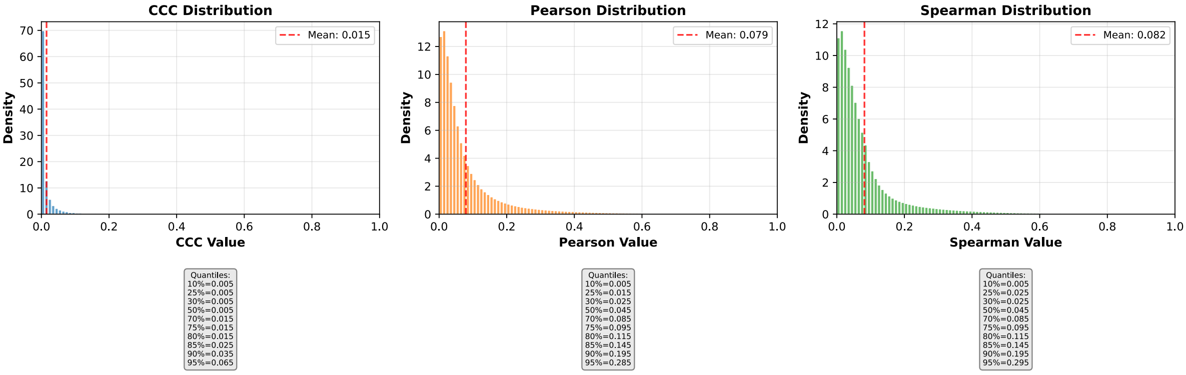

b) Corresponding cumulative histogram

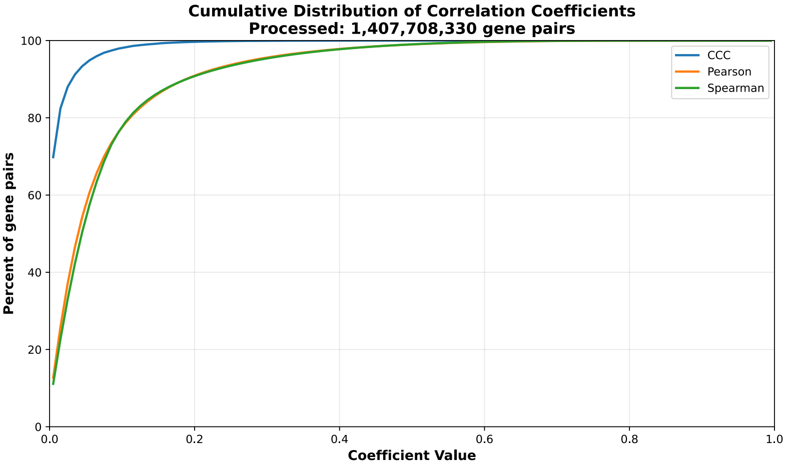

c) UpSet plot using top and bottom 30% correlations

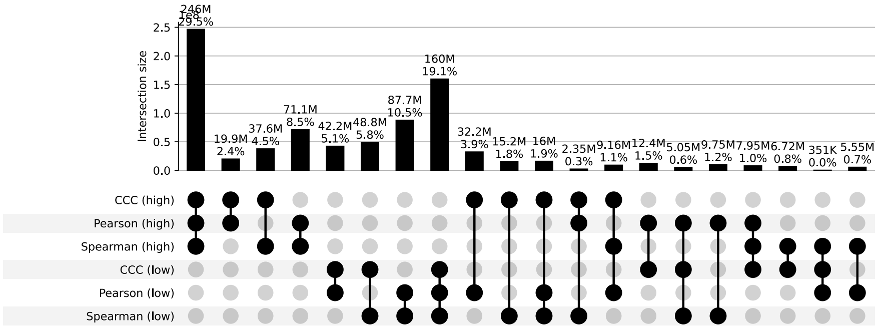

d) UpSet plot using permutation-based statistical thresholds

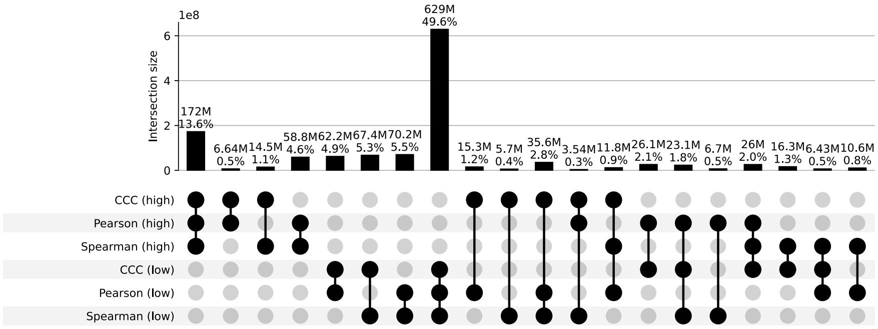

Figure S33: Distribution and UpSet plots for GTEx v8 esophagus gastroesophageal junction.

Esophagus Mucosa

a) Correlation coefficient distributions between gene pairs within GTEx v8 Esophagus Mucosa

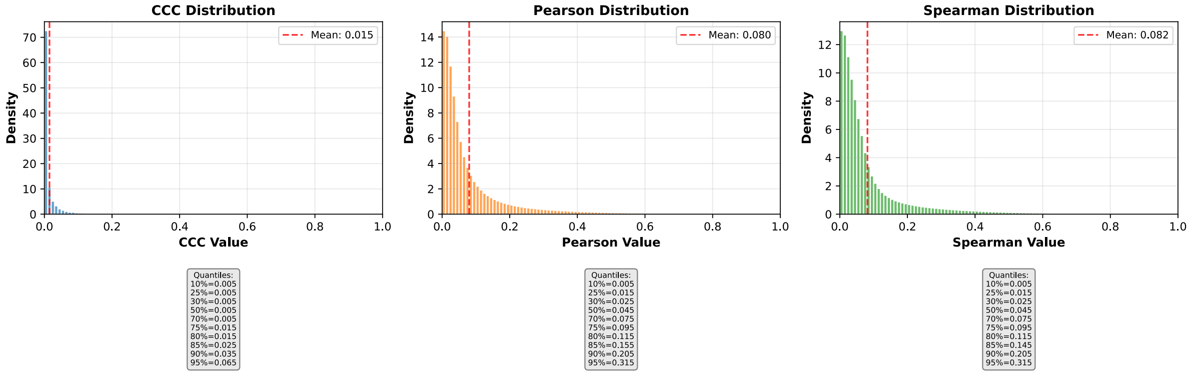

b) Corresponding cumulative histogram

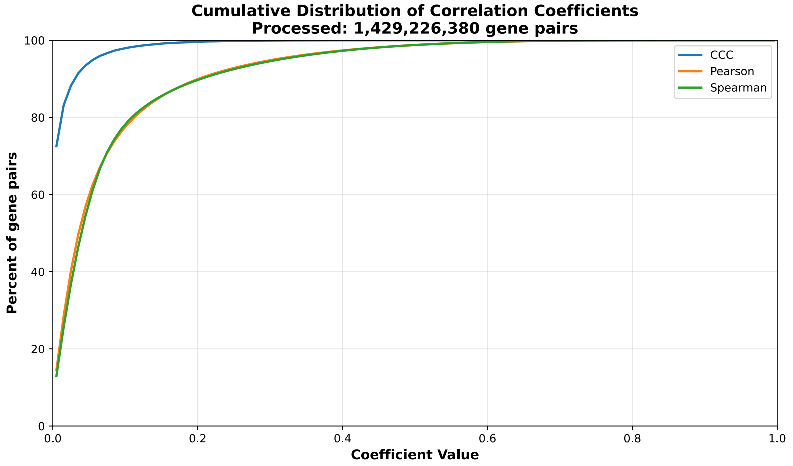

c) UpSet plot using top and bottom 30% correlations

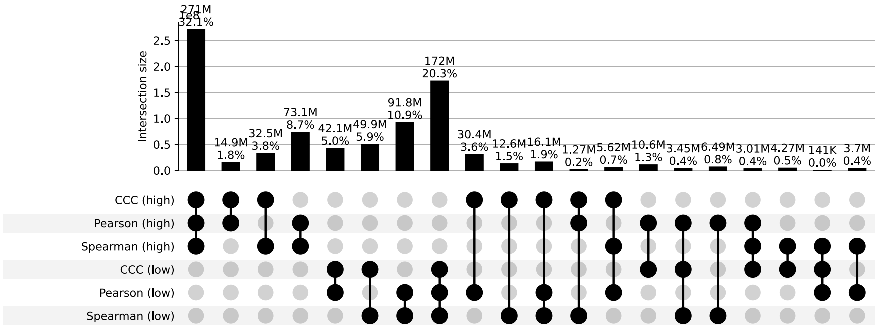

d) UpSet plot using permutation-based statistical thresholds

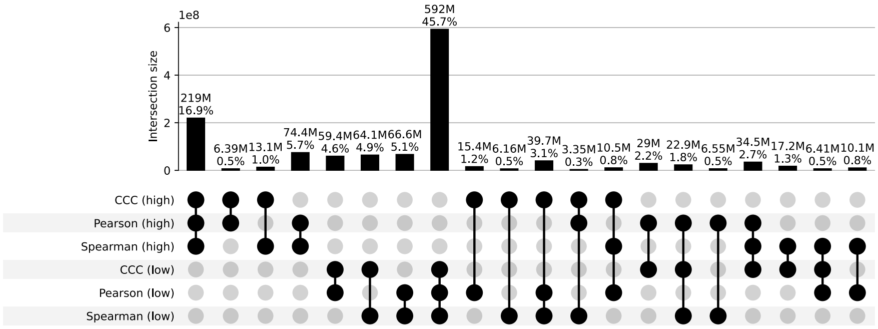

Figure S34: Distribution and UpSet plots for GTEx v8 esophagus mucosa.

Esophagus Muscularis

a) Correlation coefficient distributions between gene pairs within GTEx v8 Esophagus Muscularis

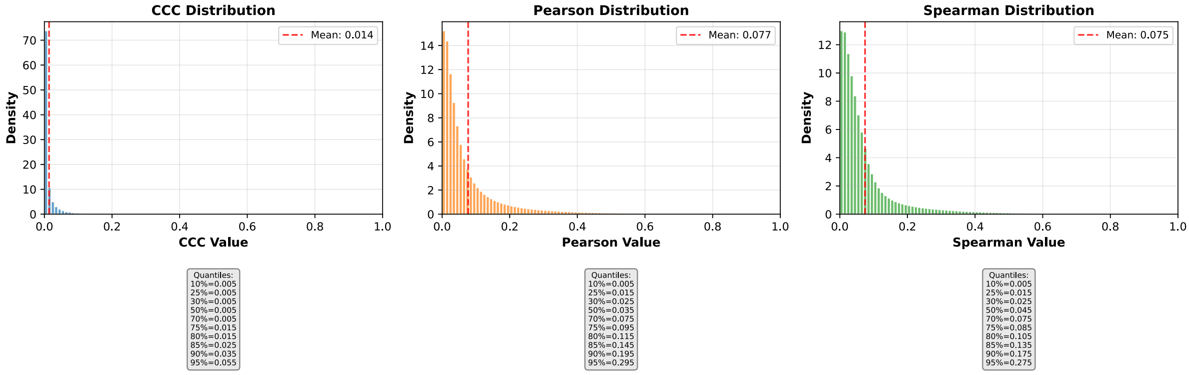

b) Corresponding cumulative histogram

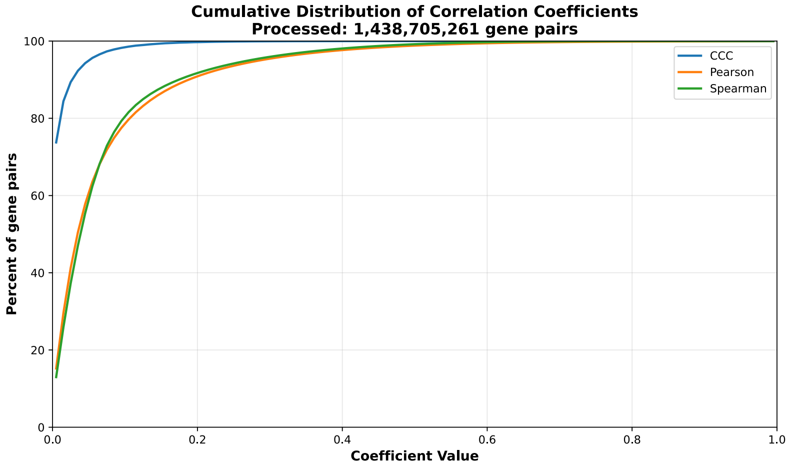

c) UpSet plot using top and bottom 30% correlations

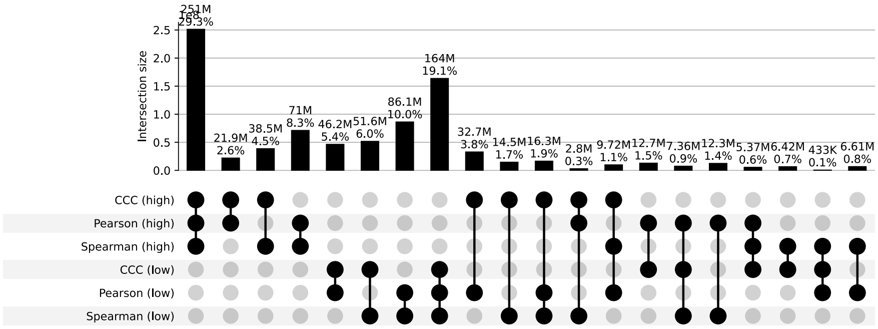

d) UpSet plot using permutation-based statistical thresholds

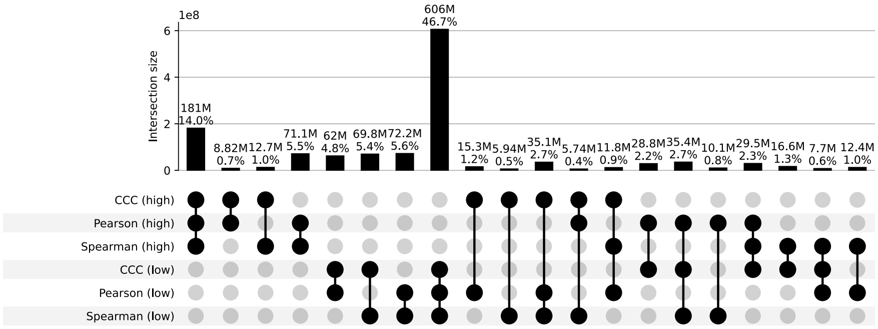

Figure S35: Distribution and UpSet plots for GTEx v8 esophagus muscularis.

Fallopian Tube

a) Correlation coefficient distributions between gene pairs within GTEx v8 Fallopian Tube

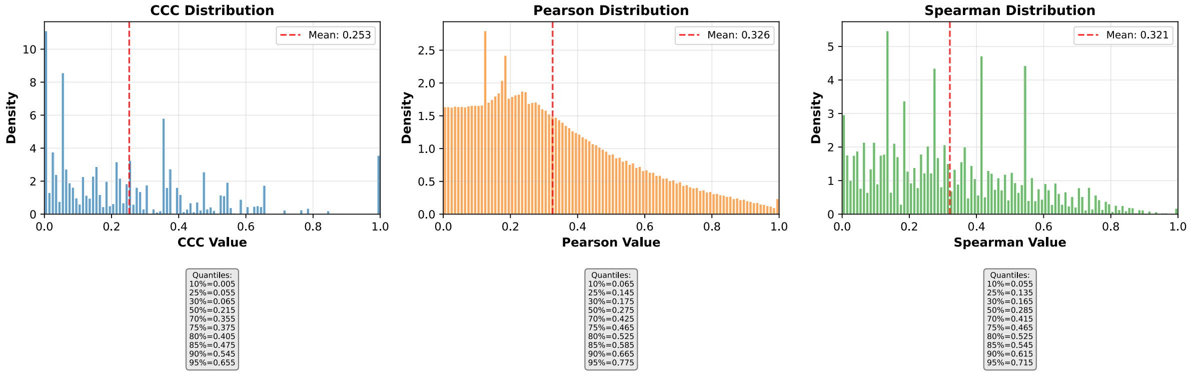

b) Corresponding cumulative histogram

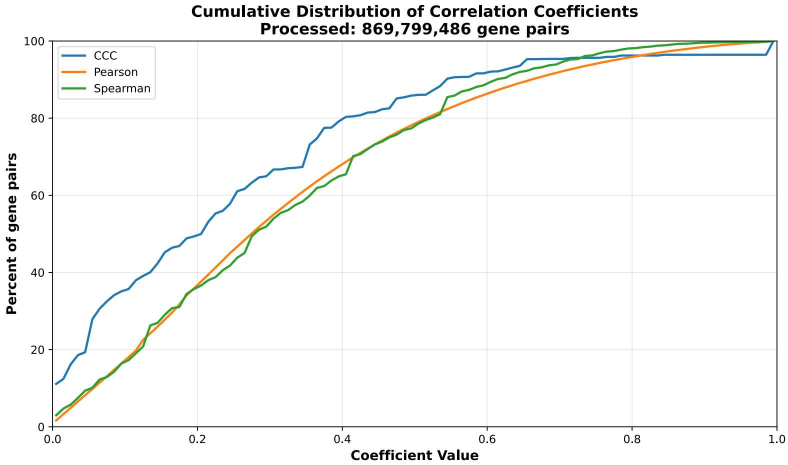

c) UpSet plot using top and bottom 30% correlations

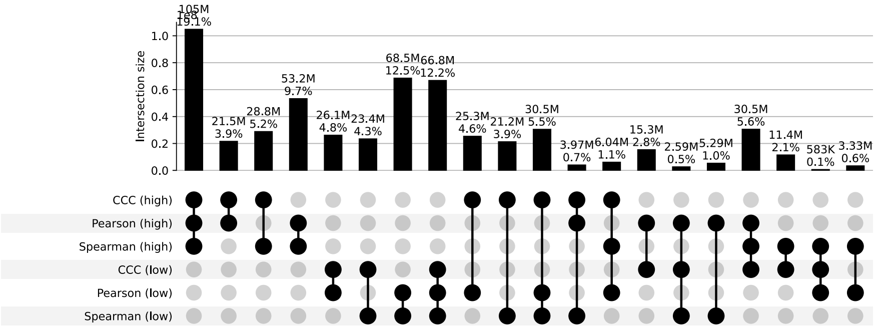

d) UpSet plot using permutation-based statistical thresholds

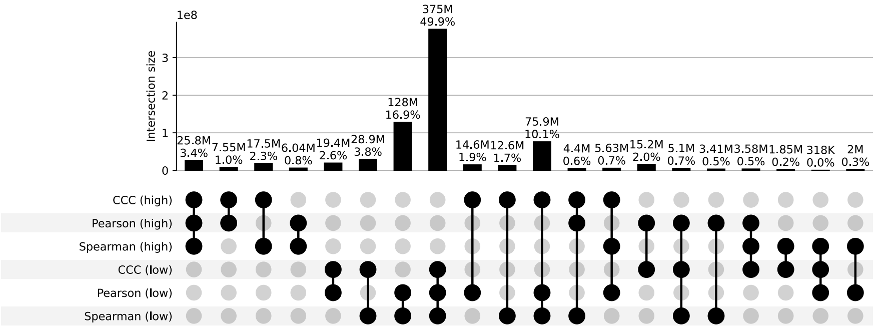

Figure S36: Distribution and UpSet plots for GTEx v8 fallopian tube.

Heart Atrial Appendage

a) Correlation coefficient distributions between gene pairs within GTEx v8 Heart Atrial Appendage

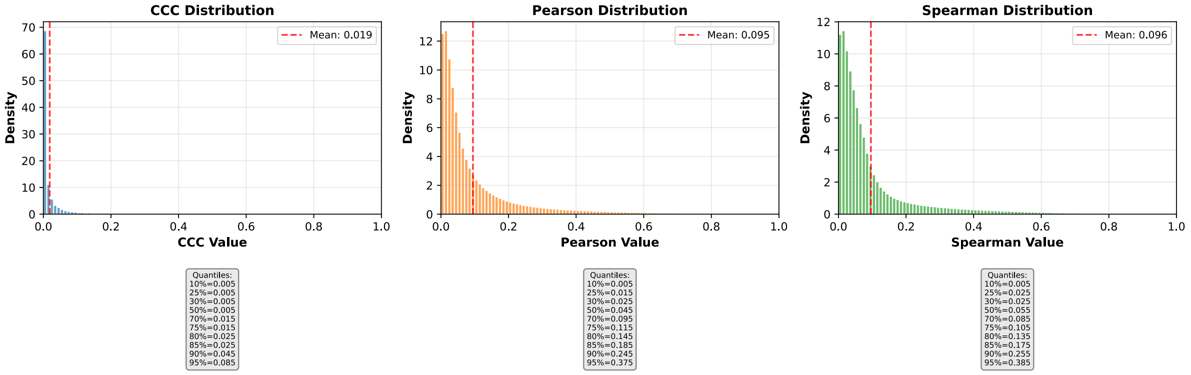

b) Corresponding cumulative histogram

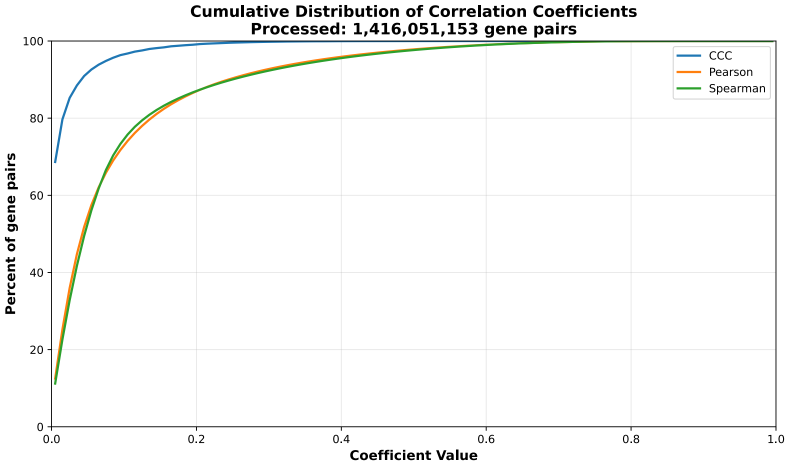

c) UpSet plot using top and bottom 30% correlations

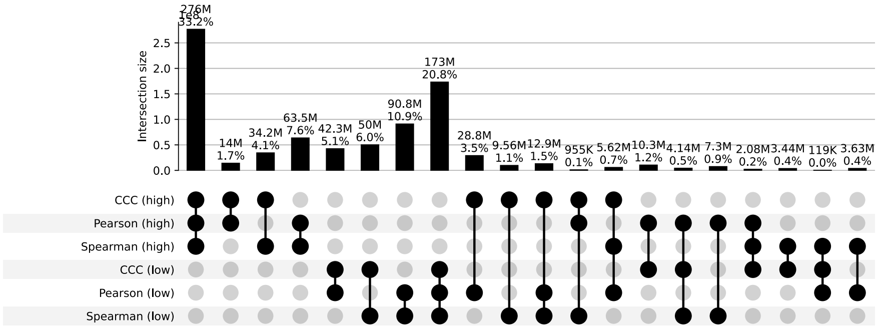

d) UpSet plot using permutation-based statistical thresholds

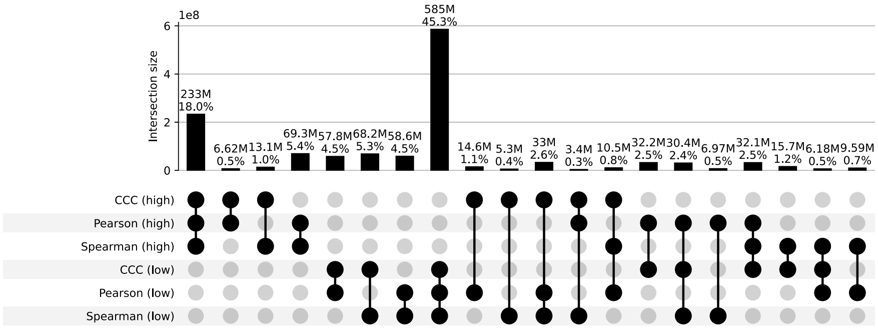

Figure S37: Distribution and UpSet plots for GTEx v8 heart atrial appendage.

Heart Left Ventricle

a) Correlation coefficient distributions between gene pairs within GTEx v8 Heart Left Ventricle

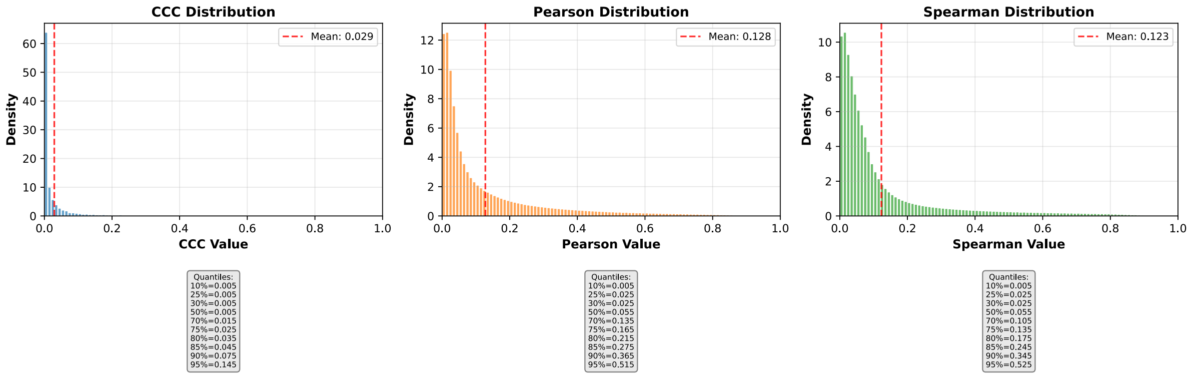

b) Corresponding cumulative histogram

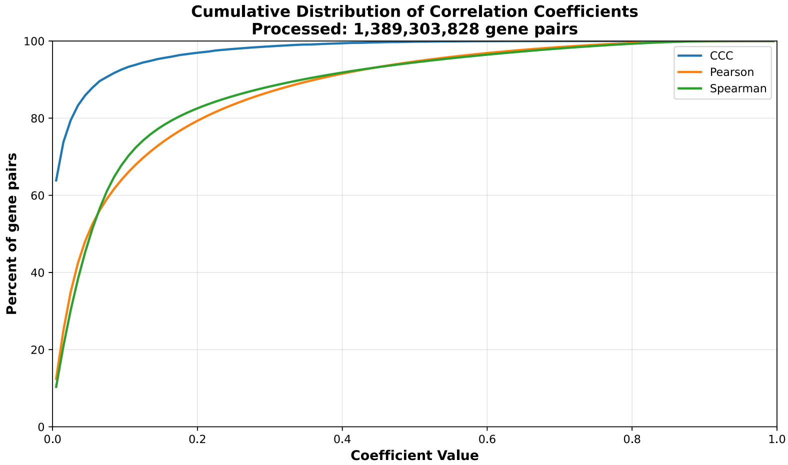

c) UpSet plot using top and bottom 30% correlations

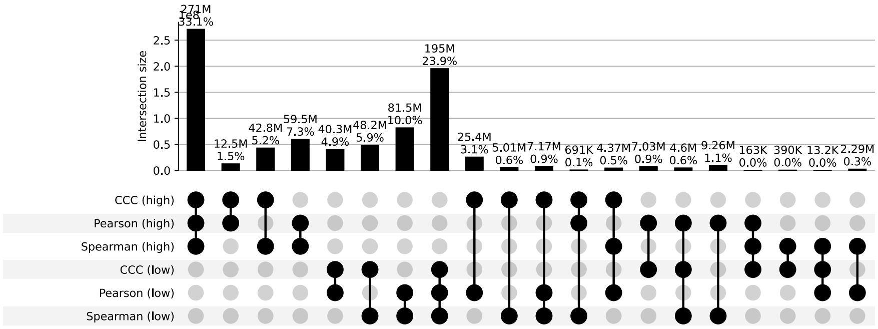

d) UpSet plot using permutation-based statistical thresholds

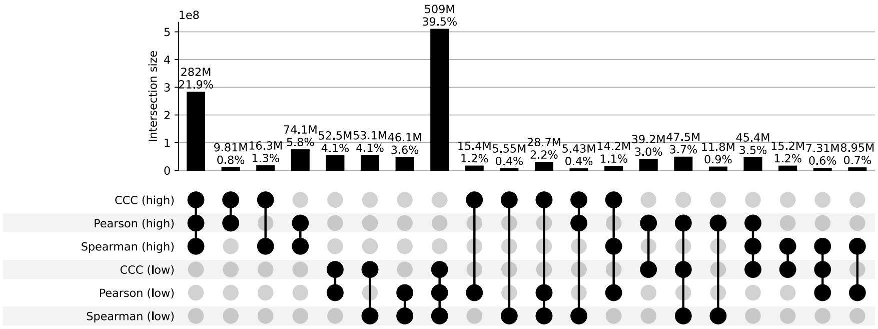

Figure S38: Distribution and UpSet plots for GTEx v8 heart left ventricle.

Kidney Cortex

a) Correlation coefficient distributions between gene pairs within GTEx v8 Kidney Cortex

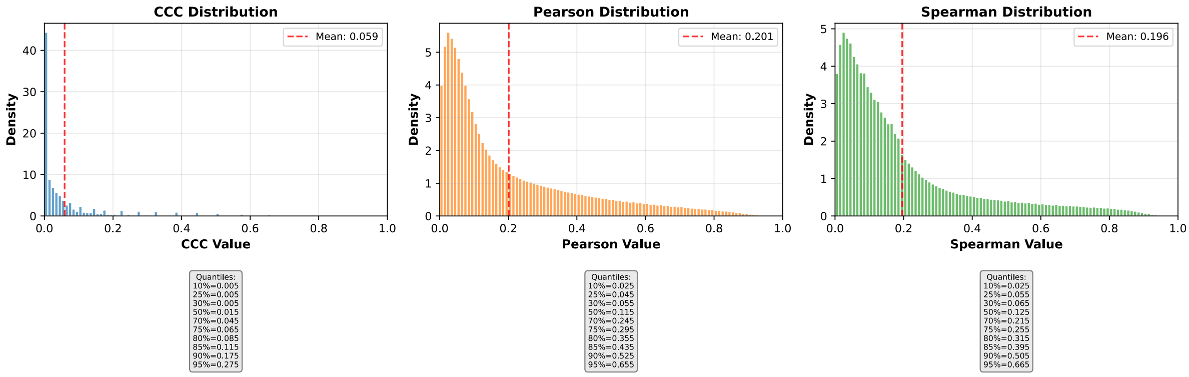

b) Corresponding cumulative histogram

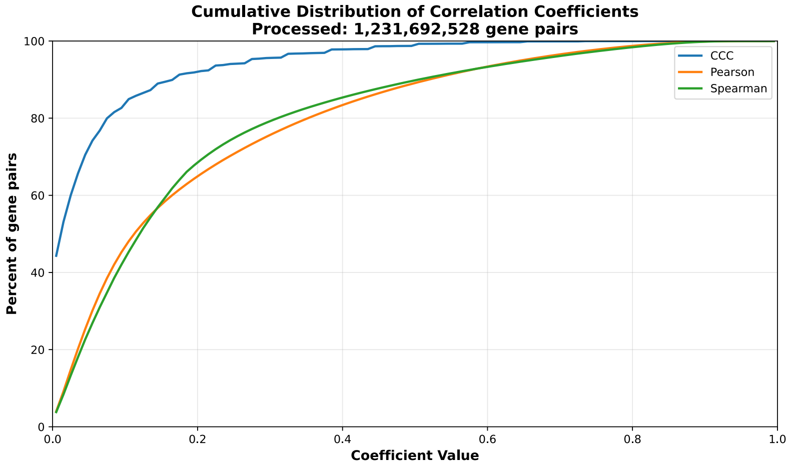

c) UpSet plot using top and bottom 30% correlations

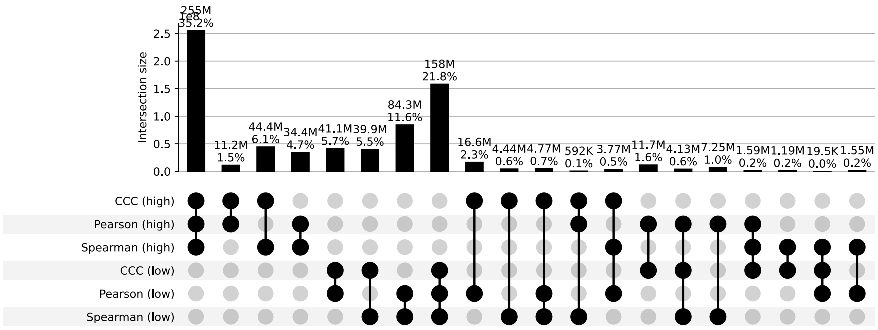

d) UpSet plot using permutation-based statistical thresholds

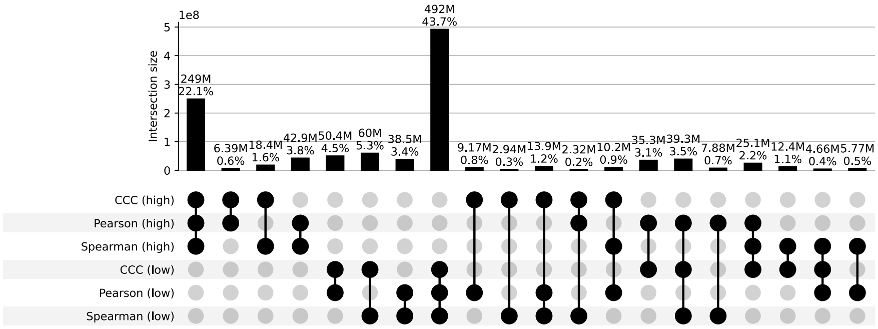

Figure S39: Distribution and UpSet plots for GTEx v8 kidney cortex.

Kidney Medulla

a) Correlation coefficient distributions between gene pairs within GTEx v8 Kidney Medulla

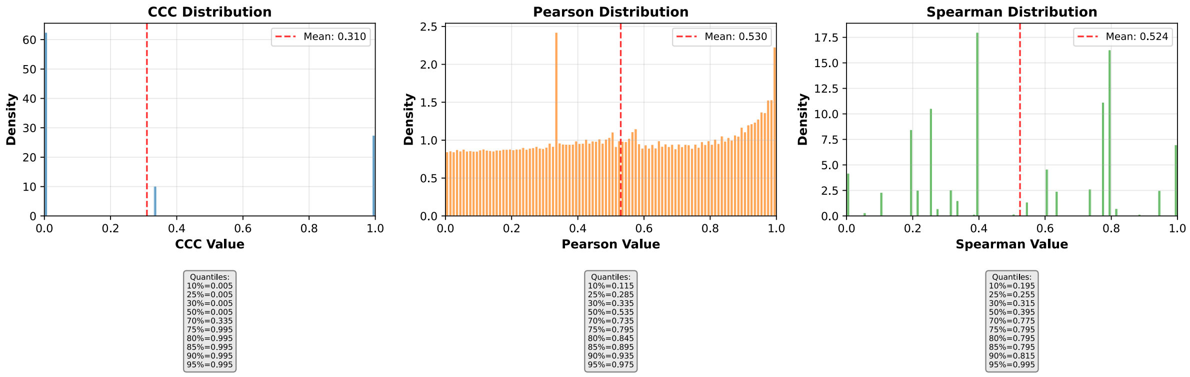

b) Corresponding cumulative histogram

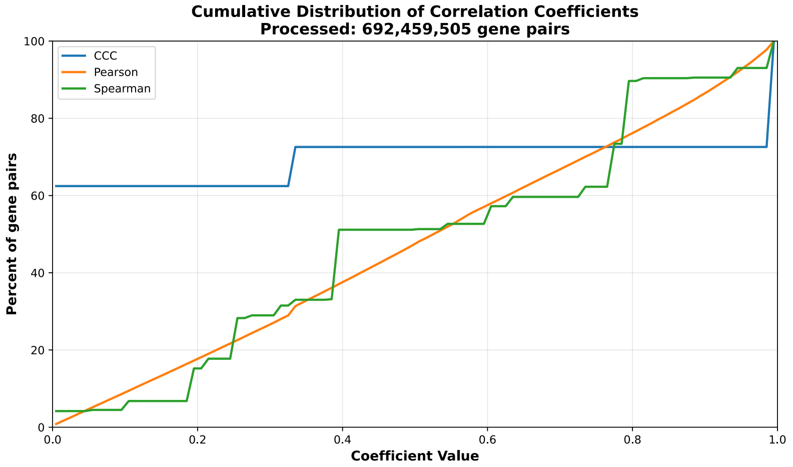

c) UpSet plot using top and bottom 30% correlations

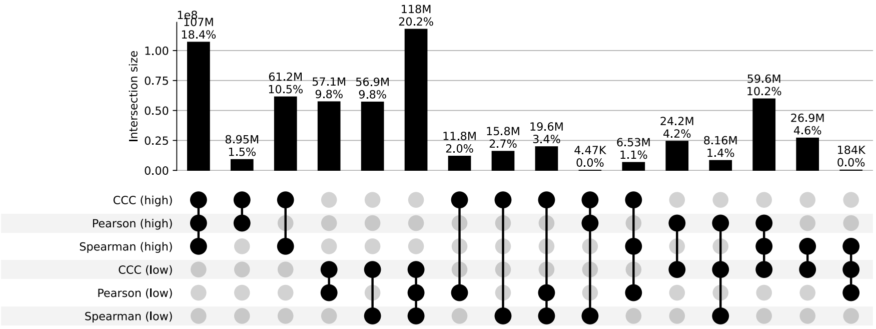

d) UpSet plot using permutation-based statistical thresholds

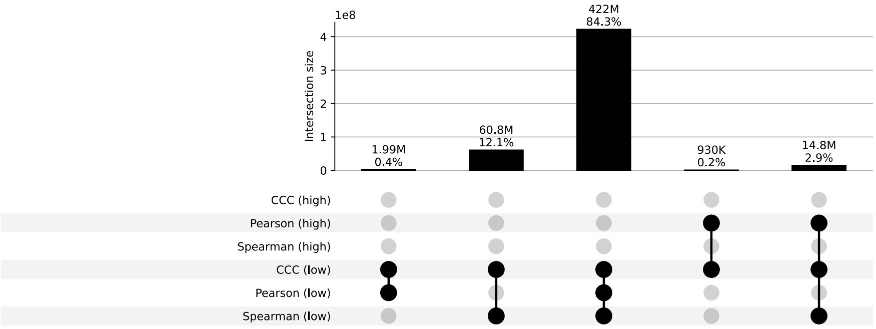

Figure S40: Distribution and UpSet plots for GTEx v8 kidney medulla.

Liver

a) Correlation coefficient distributions between gene pairs within GTEx v8 Liver

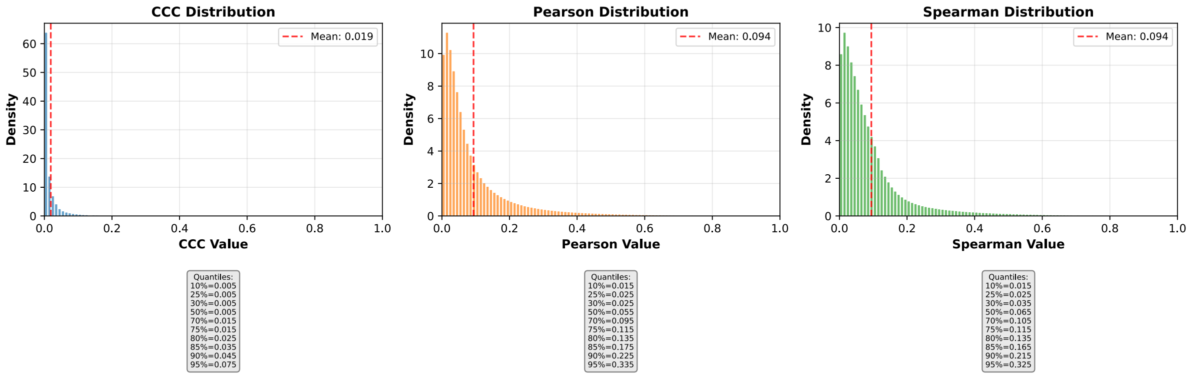

b) Corresponding cumulative histogram

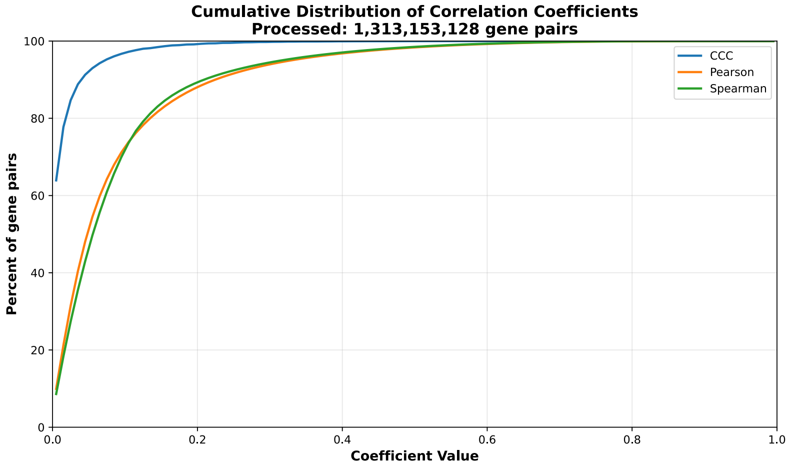

c) UpSet plot using top and bottom 30% correlations

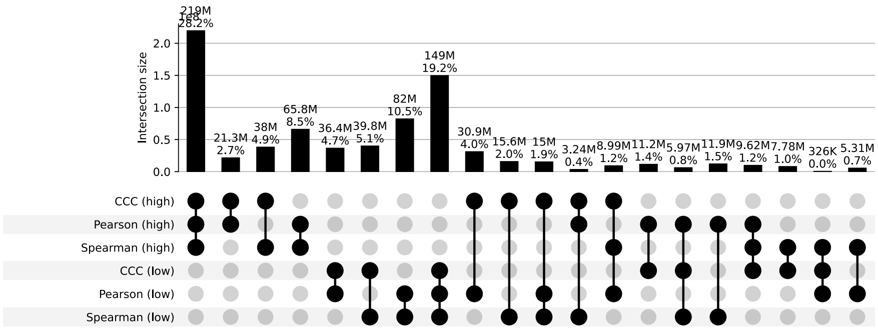

d) UpSet plot using permutation-based statistical thresholds

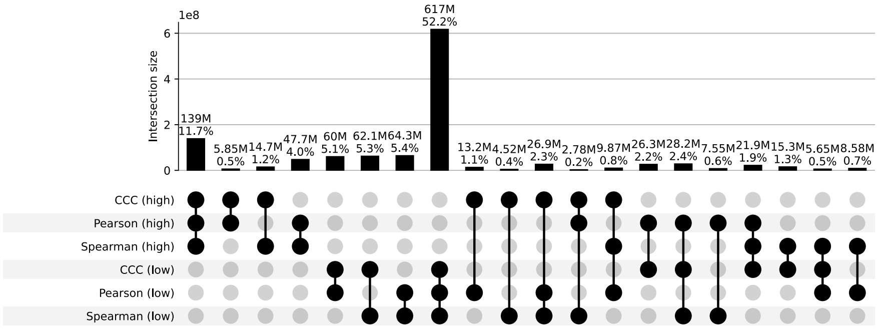

Figure S41: Distribution and UpSet plots for GTEx v8 liver.

Lung

a) Correlation coefficient distributions between gene pairs within GTEx v8 Lung

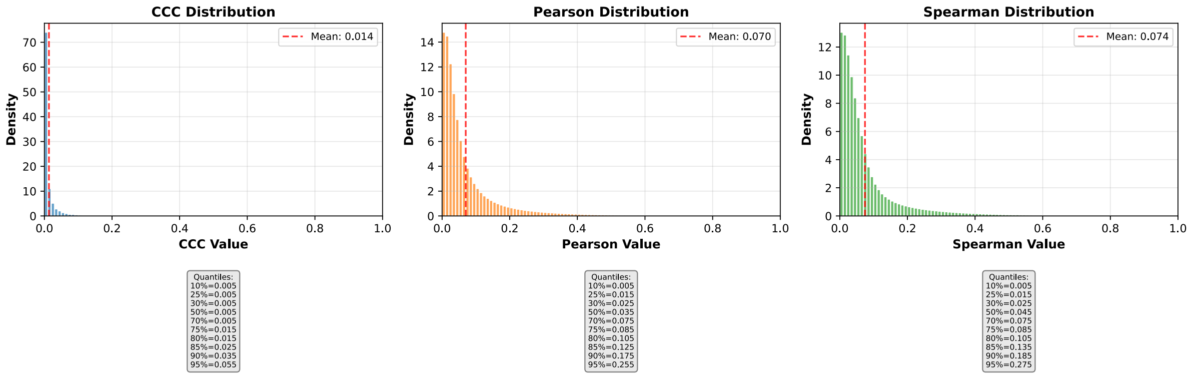

b) Corresponding cumulative histogram

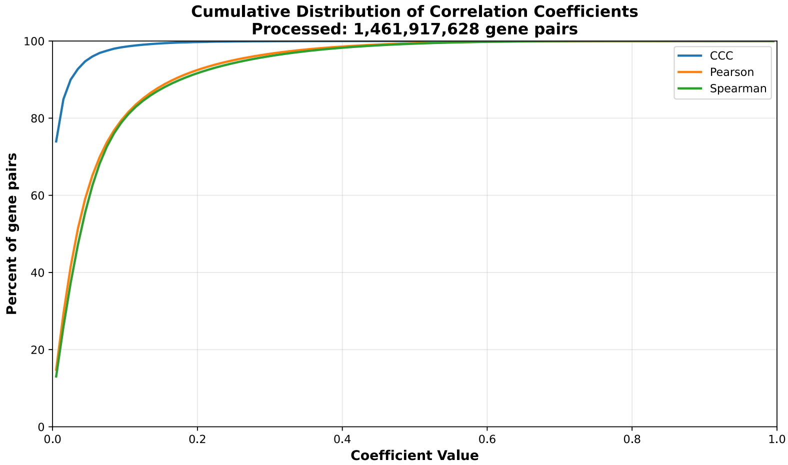

c) UpSet plot using top and bottom 30% correlations

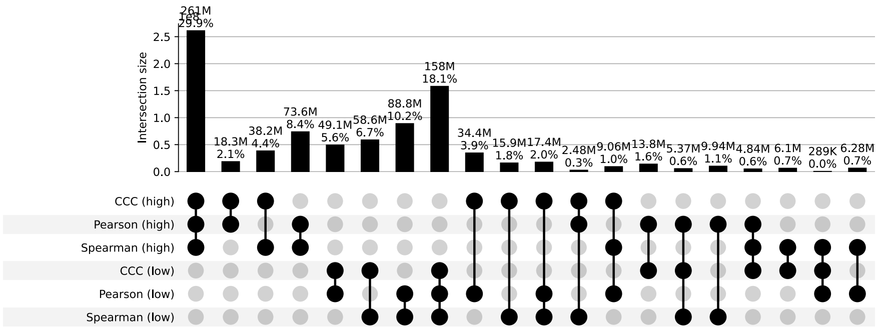

d) UpSet plot using permutation-based statistical thresholds

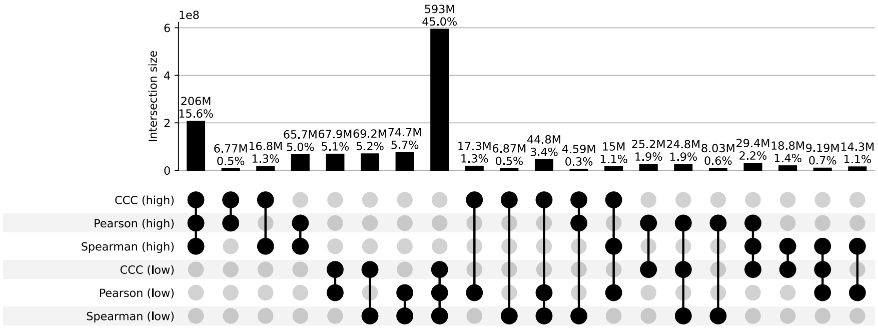

Figure S42: Distribution and UpSet plots for GTEx v8 lung.

Minor Salivary Gland

a) Correlation coefficient distributions between gene pairs within GTEx v8 Minor Salivary Gland

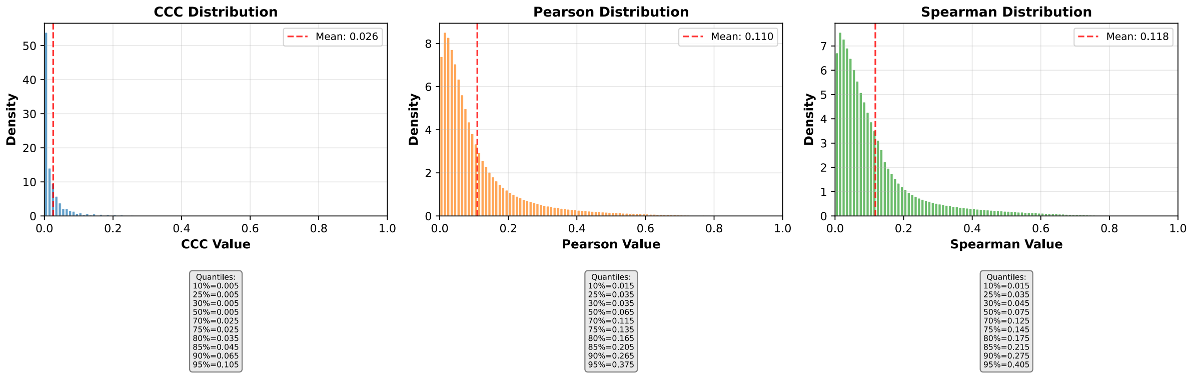

b) Corresponding cumulative histogram

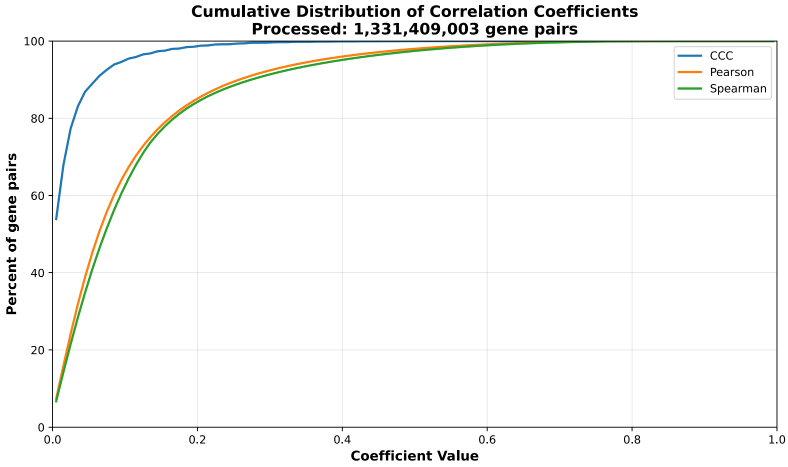

c) UpSet plot using top and bottom 30% correlations

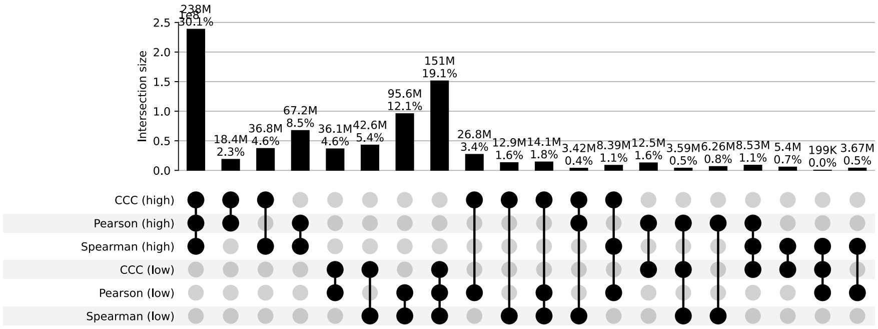

d) UpSet plot using permutation-based statistical thresholds

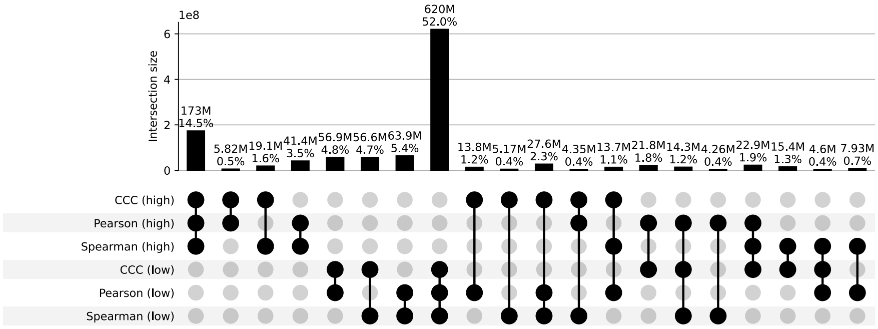

Figure S43: Distribution and UpSet plots for GTEx v8 minor salivary gland.

Muscle Skeletal

a) Correlation coefficient distributions between gene pairs within GTEx v8 Muscle Skeletal

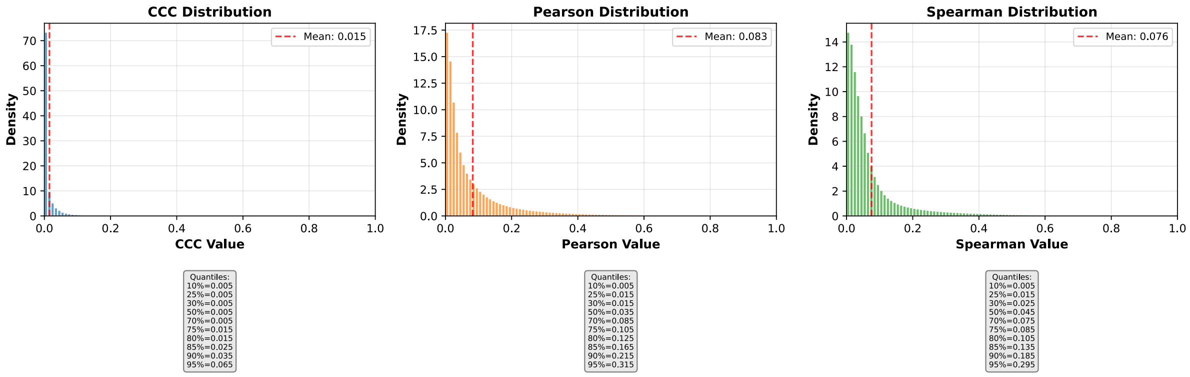

b) Corresponding cumulative histogram

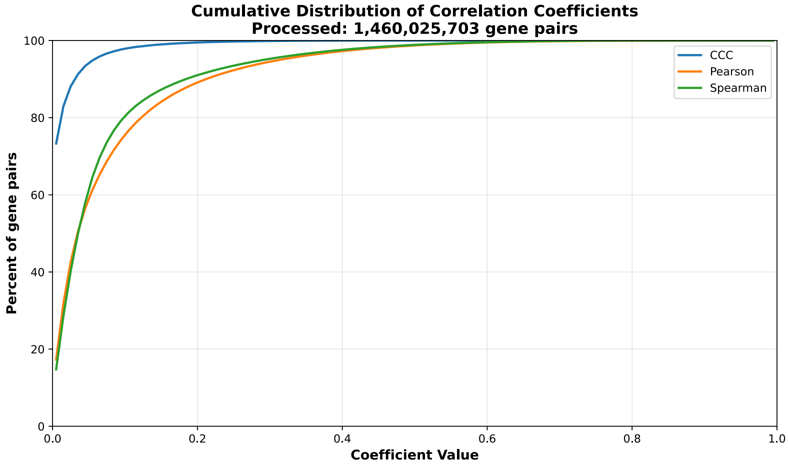

c) UpSet plot using top and bottom 30% correlations

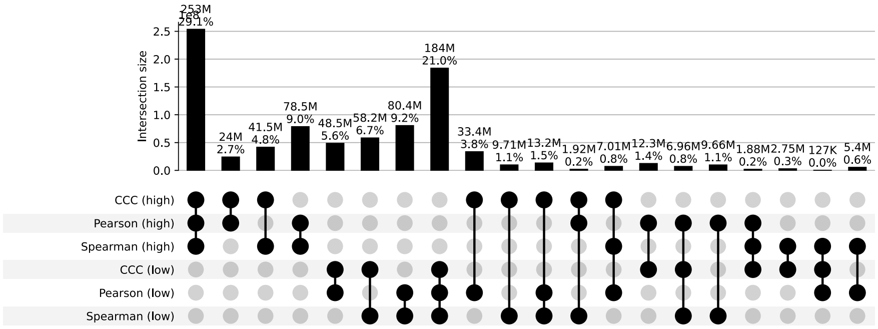

d) UpSet plot using permutation-based statistical thresholds

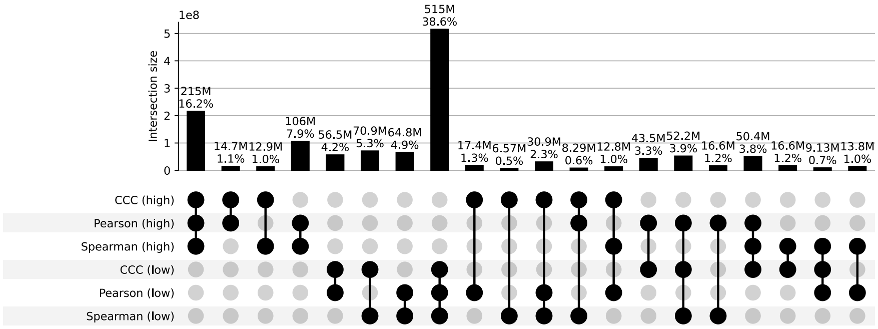

Figure S44: Distribution and UpSet plots for GTEx v8 muscle skeletal.

Nerve Tibial

a) Correlation coefficient distributions between gene pairs within GTEx v8 Nerve Tibial

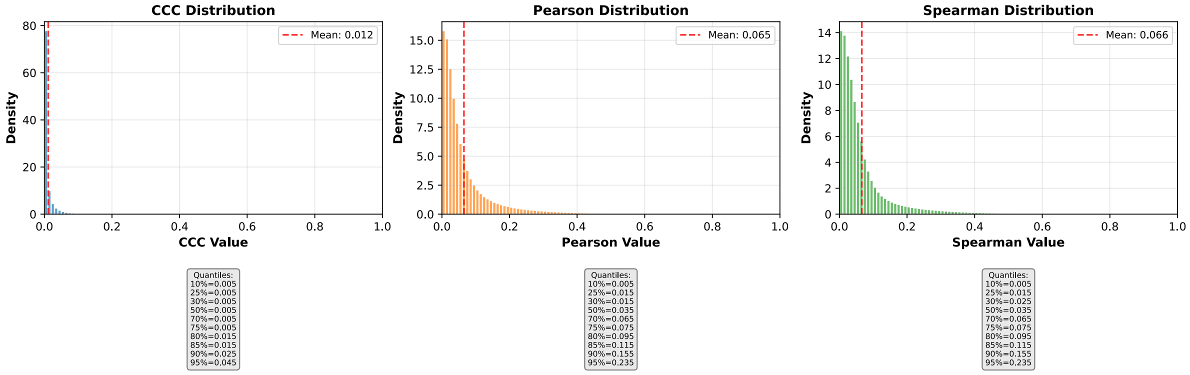

b) Corresponding cumulative histogram

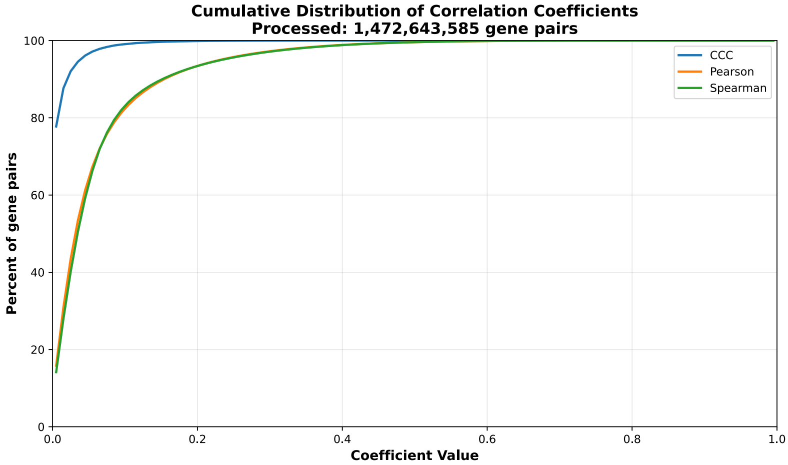

c) UpSet plot using top and bottom 30% correlations

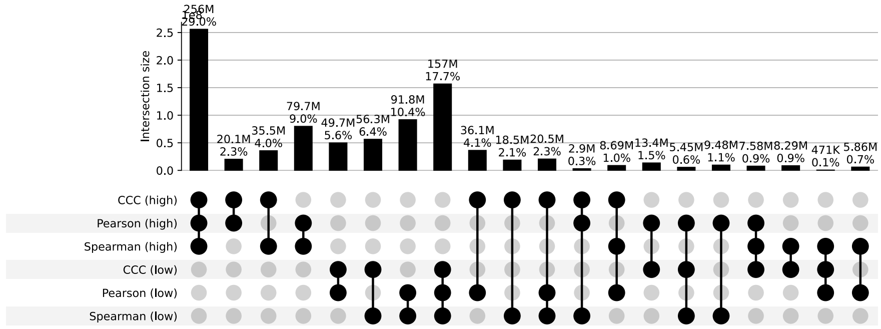

d) UpSet plot using permutation-based statistical thresholds

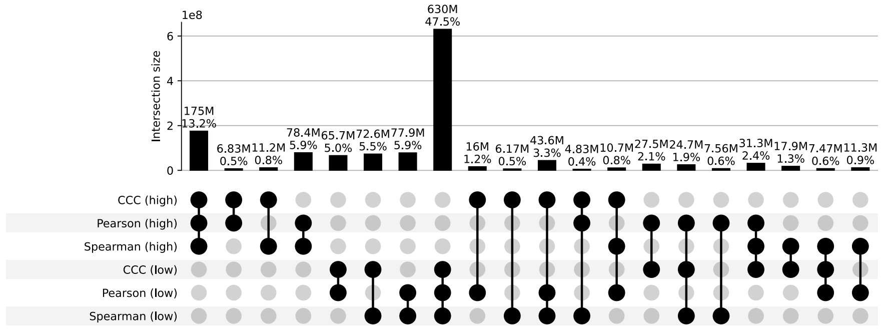

Figure S45: Distribution and UpSet plots for GTEx v8 nerve tibial.

Ovary

a) Correlation coefficient distributions between gene pairs within GTEx v8 Ovary

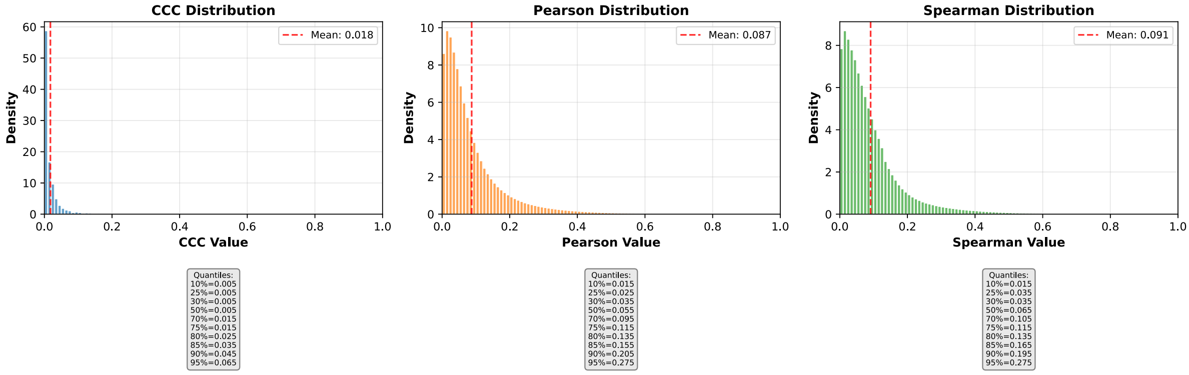

b) Corresponding cumulative histogram

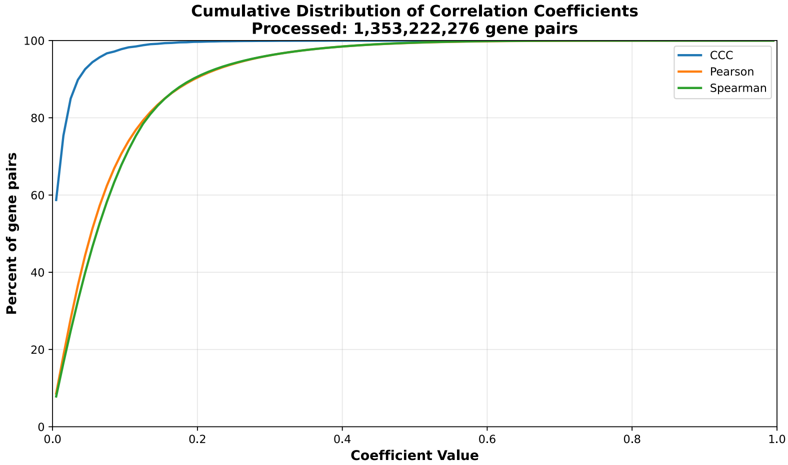

c) UpSet plot using top and bottom 30% correlations

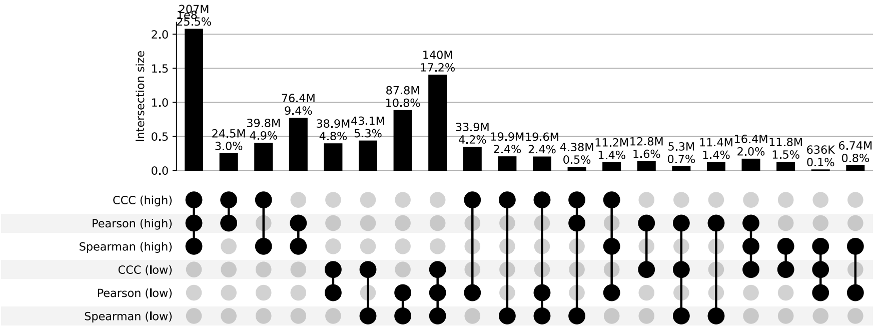

d) UpSet plot using permutation-based statistical thresholds

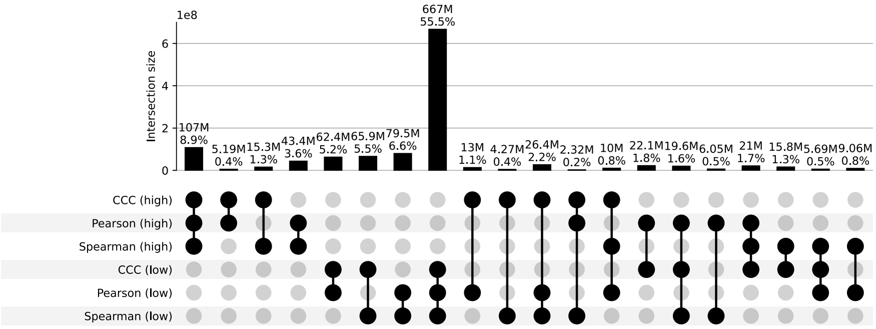

Figure S46: Distribution and UpSet plots for GTEx v8 ovary.

Pancreas

a) Correlation coefficient distributions between gene pairs within GTEx v8 Pancreas

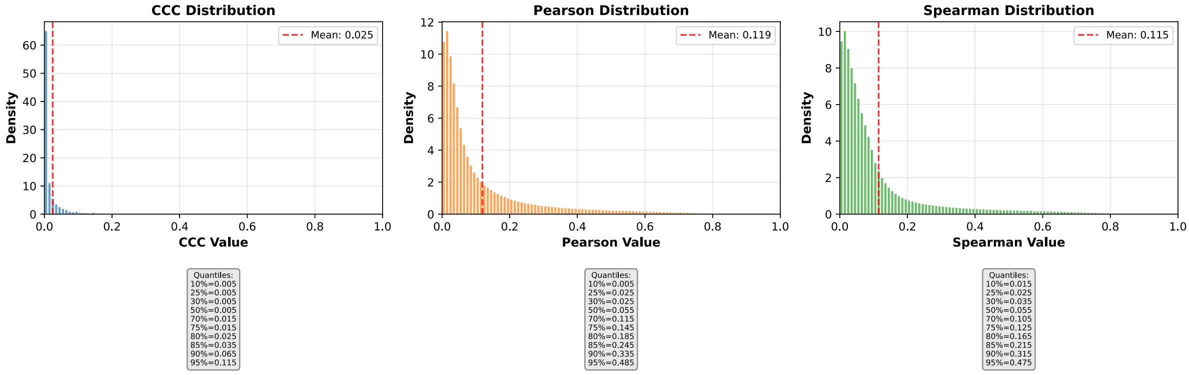

b) Corresponding cumulative histogram

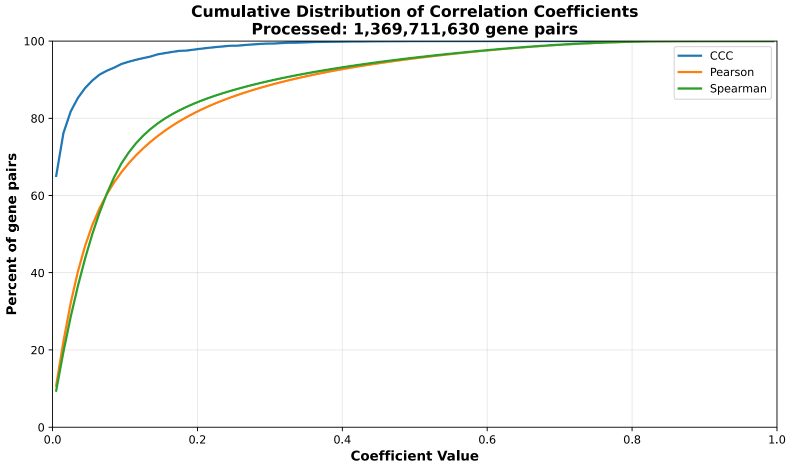

c) UpSet plot using top and bottom 30% correlations

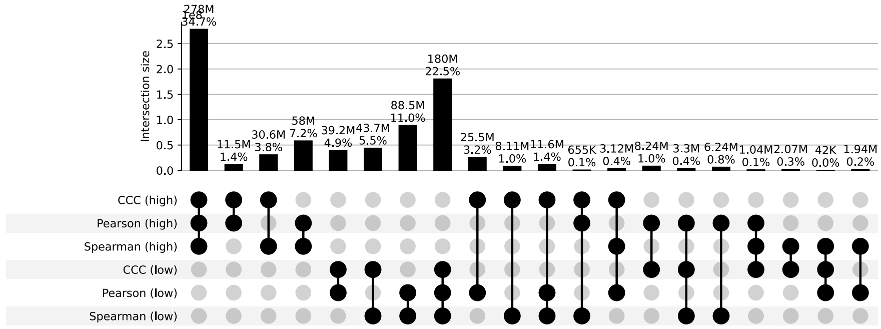

d) UpSet plot using permutation-based statistical thresholds

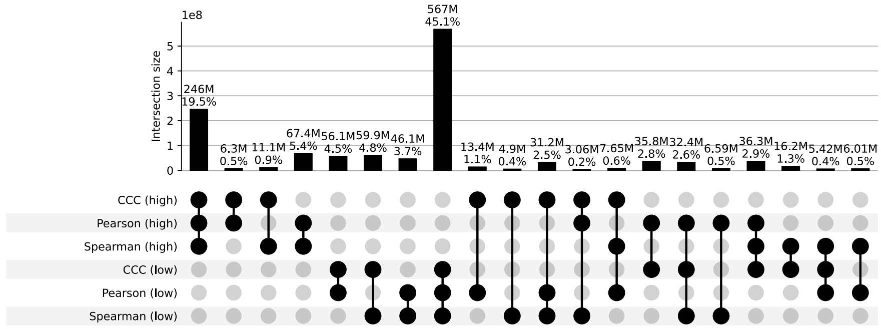

Figure S47: Distribution and UpSet plots for GTEx v8 pancreas.

Pituitary

a) Correlation coefficient distributions between gene pairs within GTEx v8 Pituitary

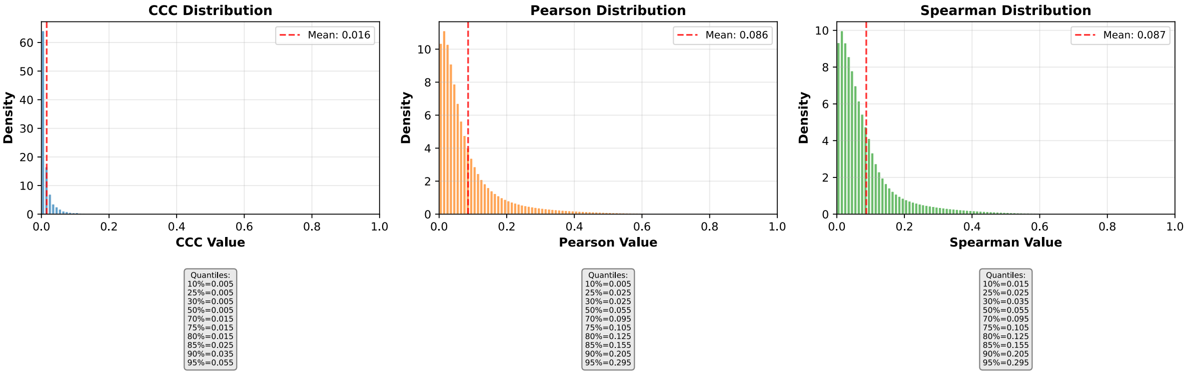

b) Corresponding cumulative histogram

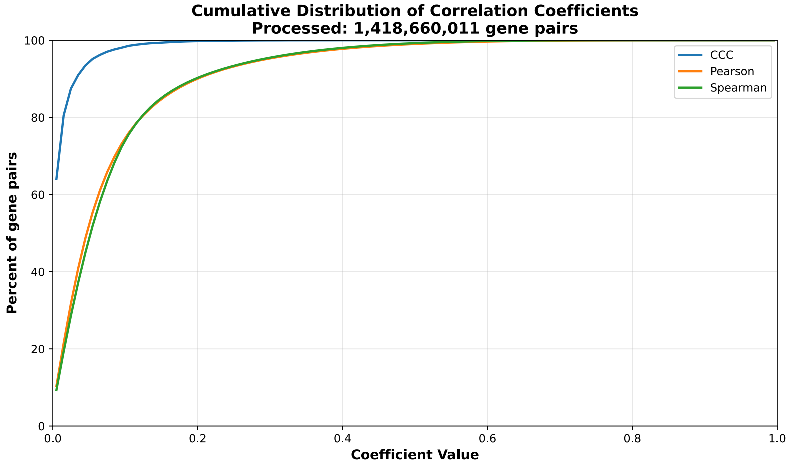

c) UpSet plot using top and bottom 30% correlations

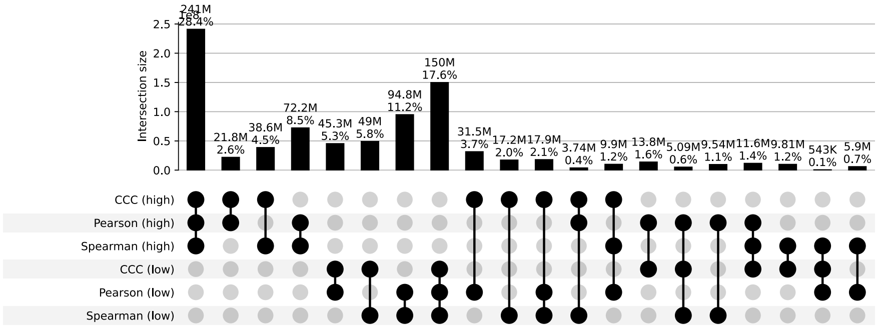

d) UpSet plot using permutation-based statistical thresholds

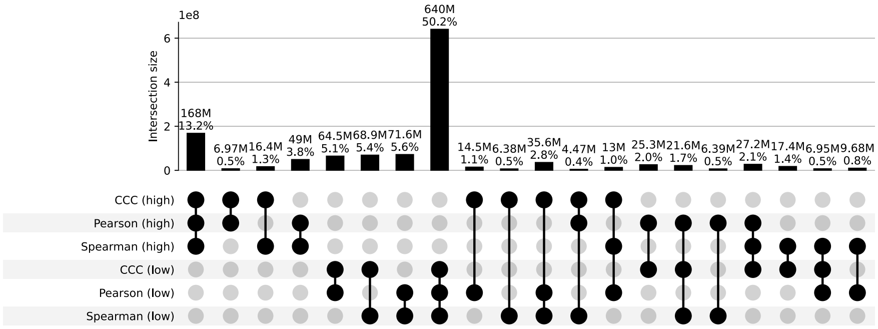

Figure S48: Distribution and UpSet plots for GTEx v8 pituitary.

Prostate

a) Correlation coefficient distributions between gene pairs within GTEx v8 Prostate

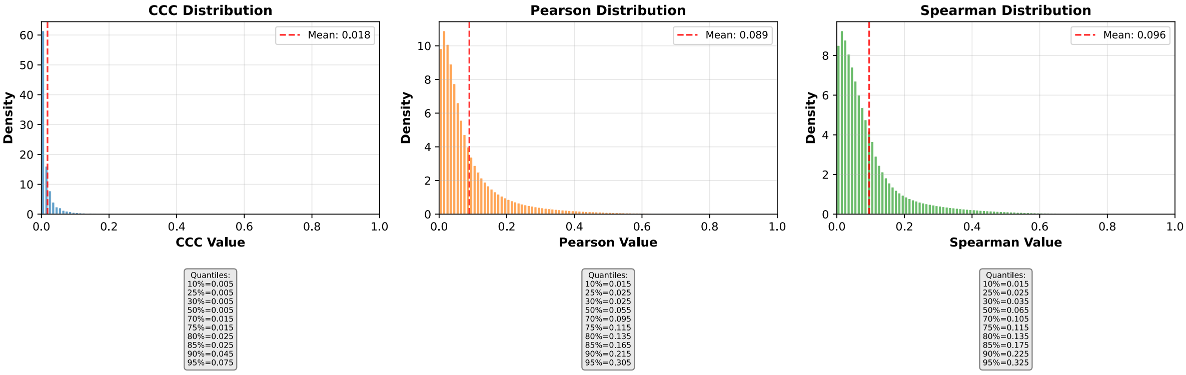

b) Corresponding cumulative histogram

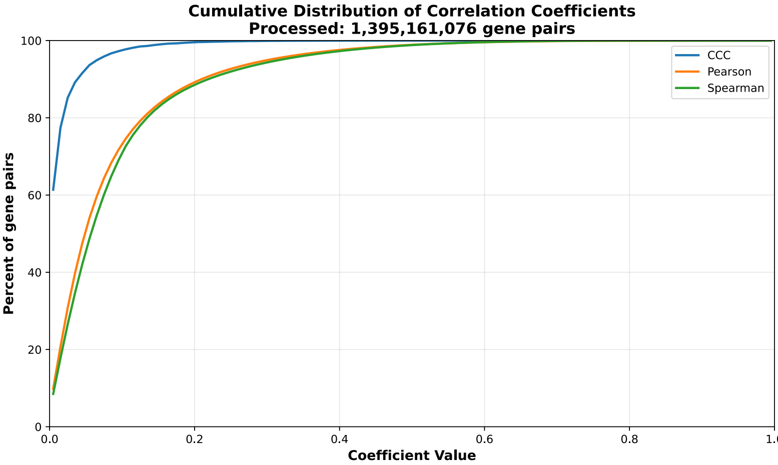

c) UpSet plot using top and bottom 30% correlations

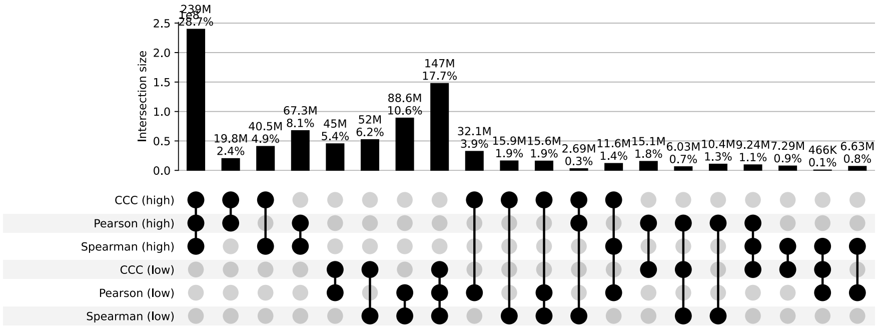

d) UpSet plot using permutation-based statistical thresholds

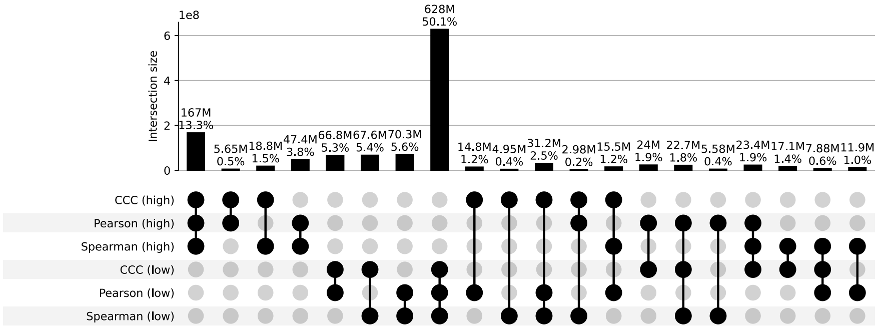

Figure S49: Distribution and UpSet plots for GTEx v8 prostate.

Skin Not Sun Exposed Suprapubic

a) Correlation coefficient distributions between gene pairs within GTEx v8 Skin Not Sun Exposed Suprapubic

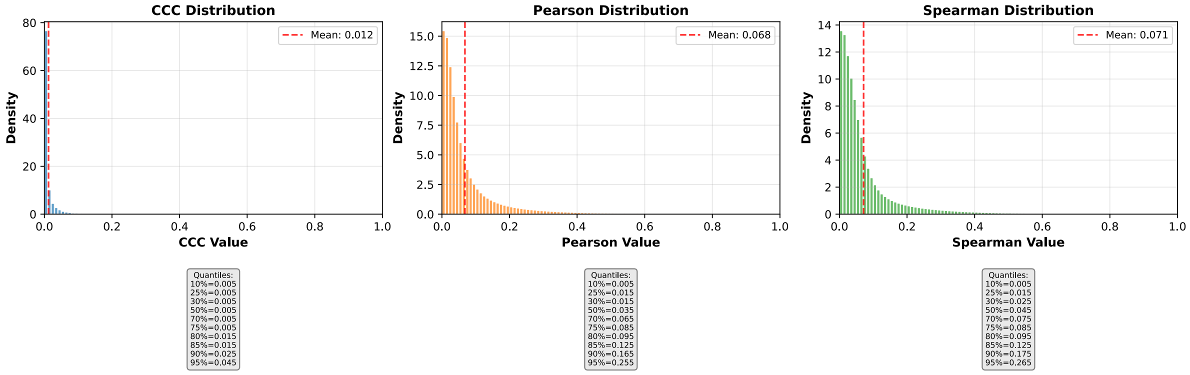

b) Corresponding cumulative histogram

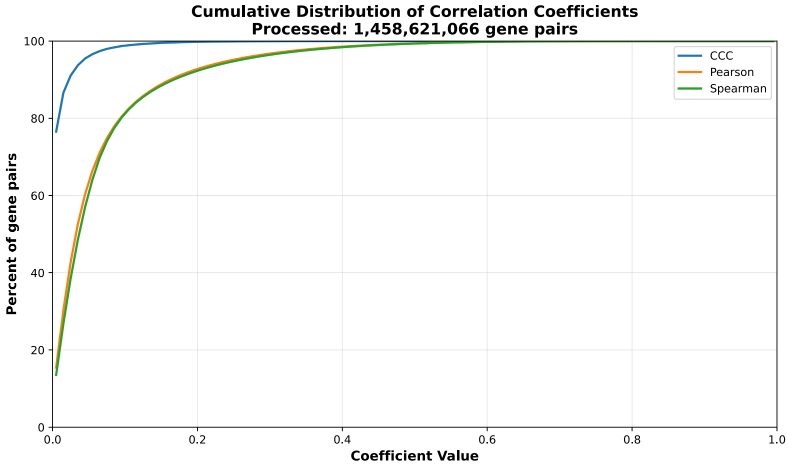

c) UpSet plot using top and bottom 30% correlations

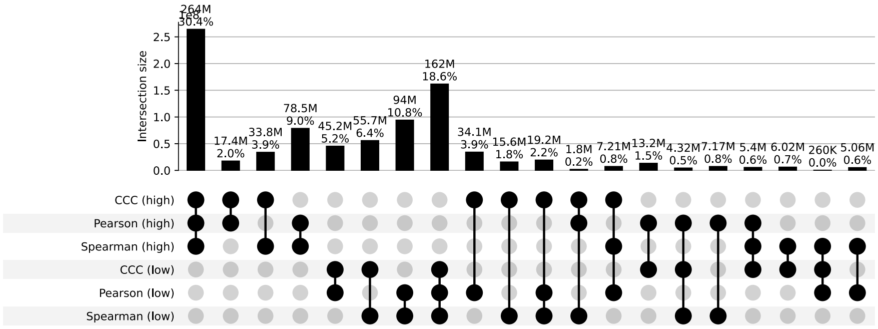

d) UpSet plot using permutation-based statistical thresholds

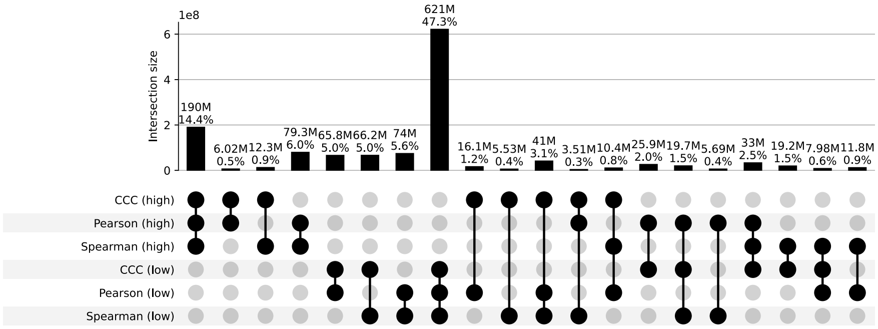

Figure S50: Distribution and UpSet plots for GTEx v8 skin not sun exposed suprapubic.

Skin Sun Exposed Lower Leg

a) Correlation coefficient distributions between gene pairs within GTEx v8 Skin Sun Exposed Lower Leg

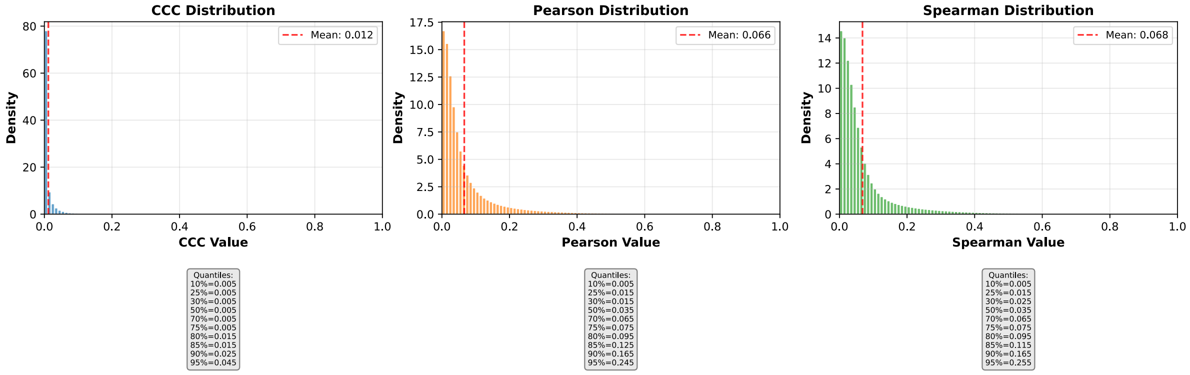

b) Corresponding cumulative histogram

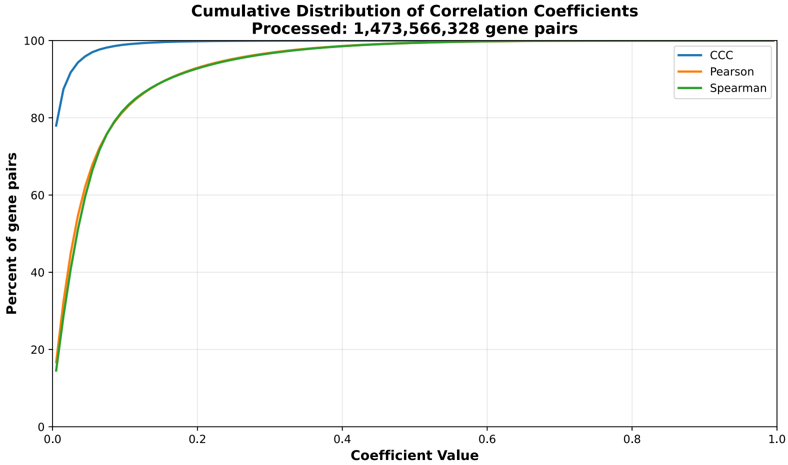

c) UpSet plot using top and bottom 30% correlations

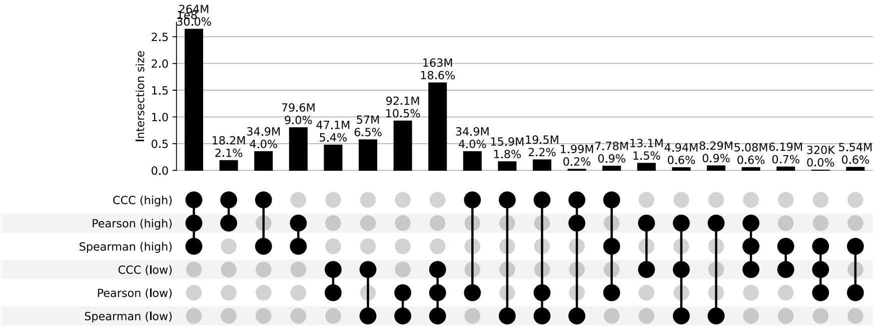

d) UpSet plot using permutation-based statistical thresholds

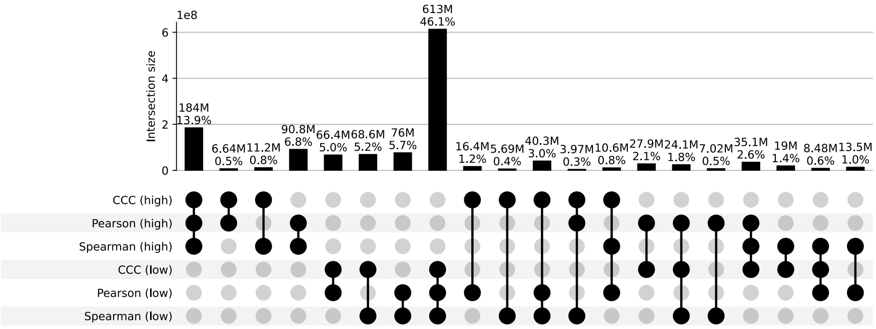

Figure S51: Distribution and UpSet plots for GTEx v8 skin sun exposed lower leg.

Small Intestine Terminal Ileum

a) Correlation coefficient distributions between gene pairs within GTEx v8 Small Intestine Terminal Ileum

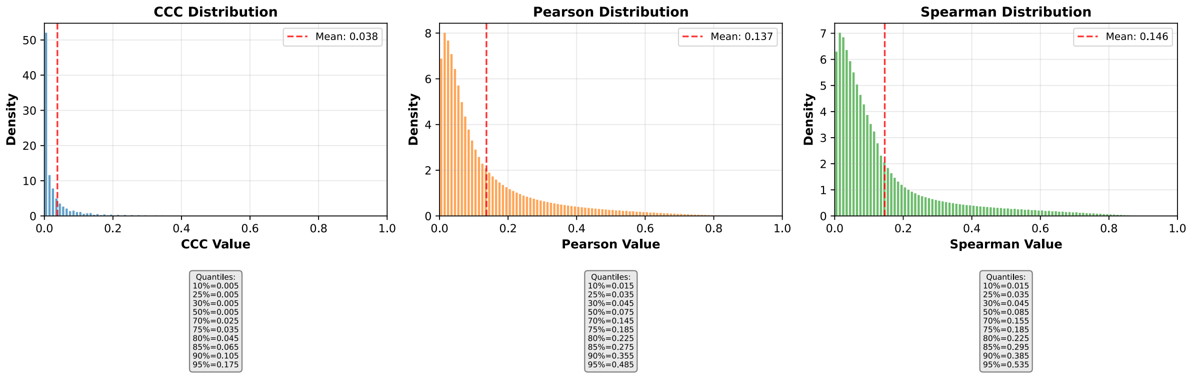

b) Corresponding cumulative histogram

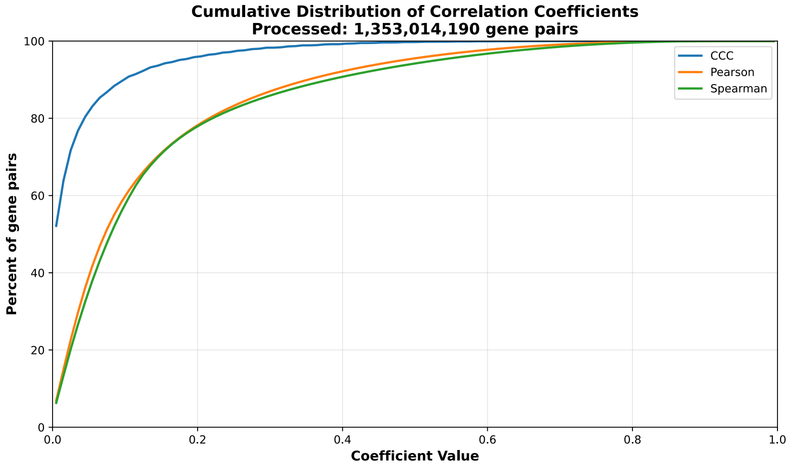

c) UpSet plot using top and bottom 30% correlations

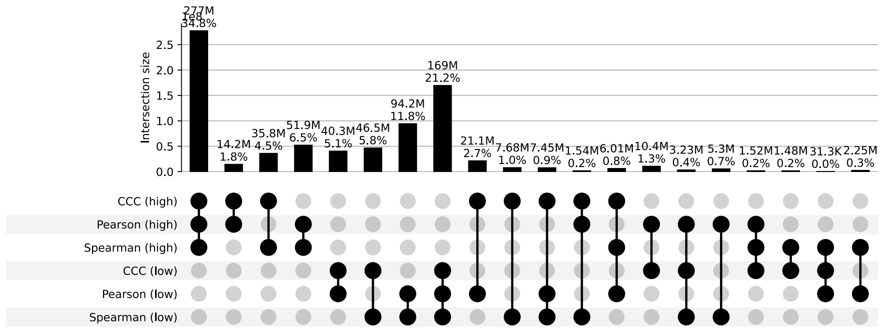

d) UpSet plot using permutation-based statistical thresholds

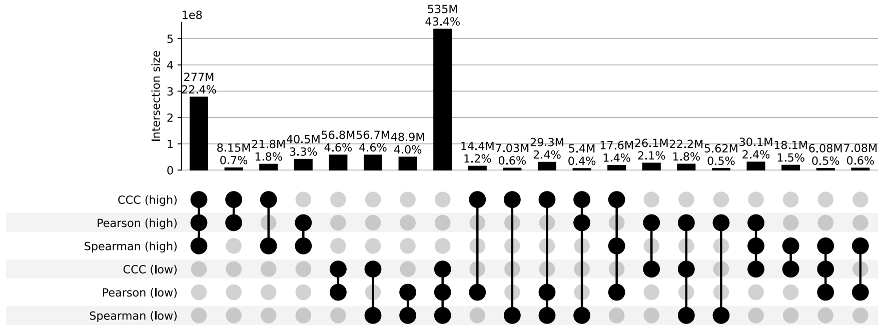

Figure S52: Distribution and UpSet plots for GTEx v8 small intestine terminal ileum.

Spleen

a) Correlation coefficient distributions between gene pairs within GTEx v8 Spleen

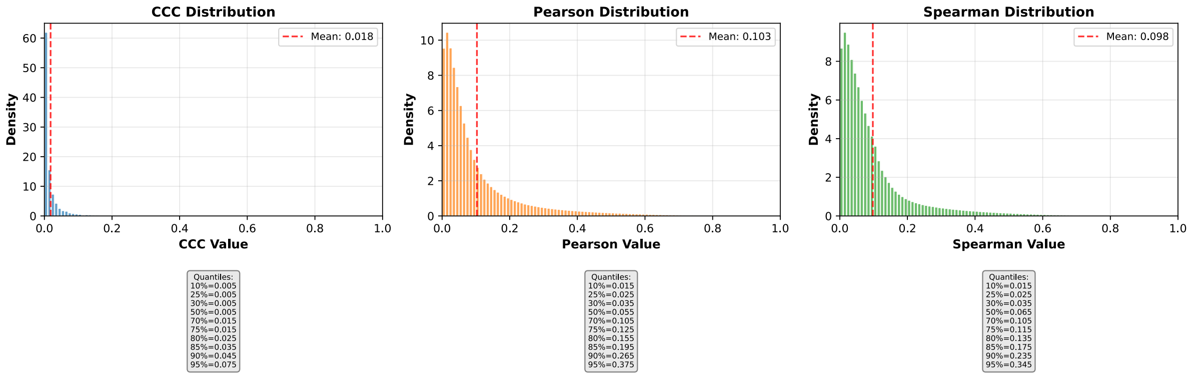

b) Corresponding cumulative histogram

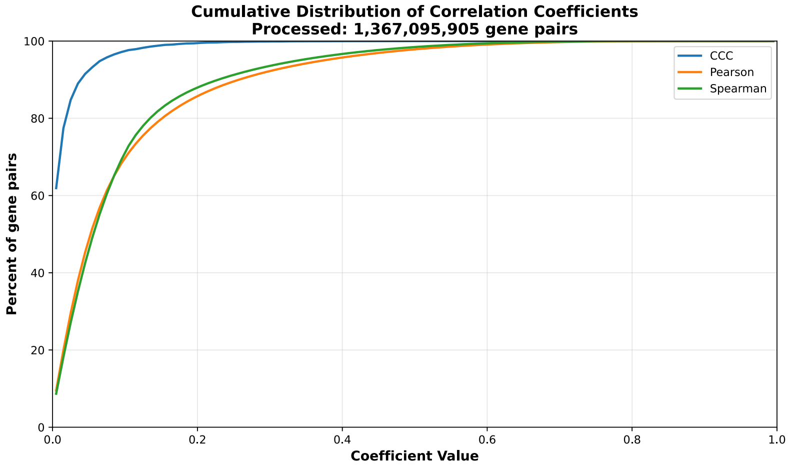

c) UpSet plot using top and bottom 30% correlations

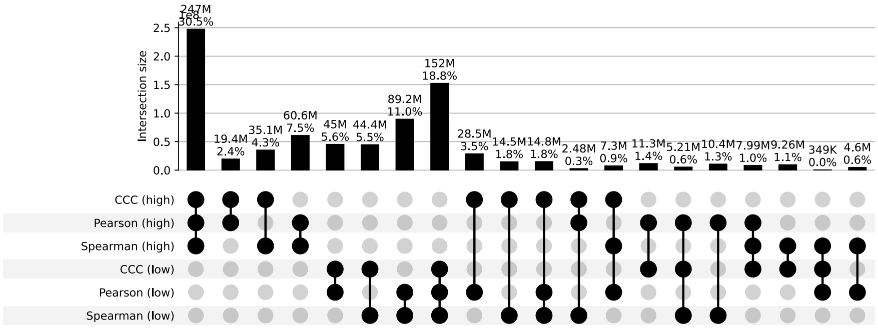

d) UpSet plot using permutation-based statistical thresholds

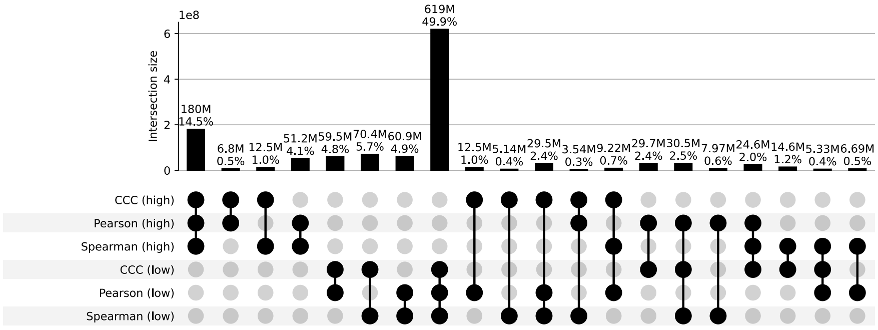

Figure S53: Distribution and UpSet plots for GTEx v8 spleen.

Stomach

a) Correlation coefficient distributions between gene pairs within GTEx v8 Stomach

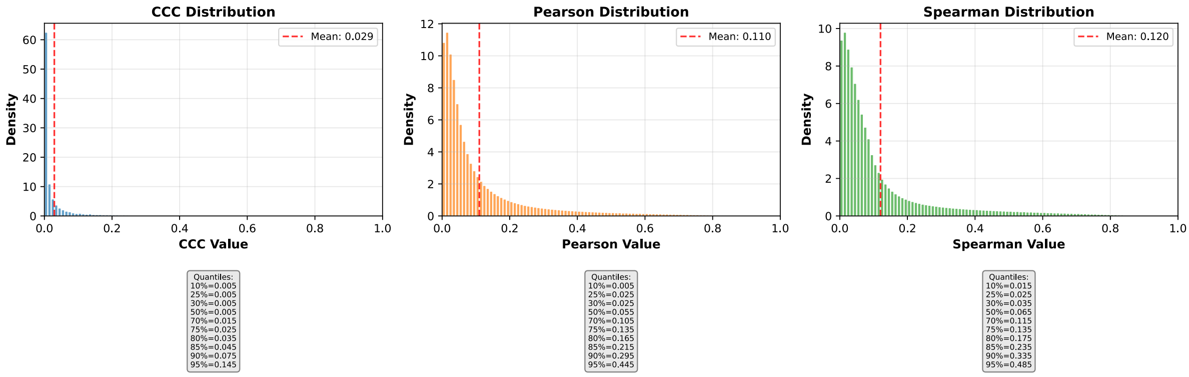

b) Corresponding cumulative histogram

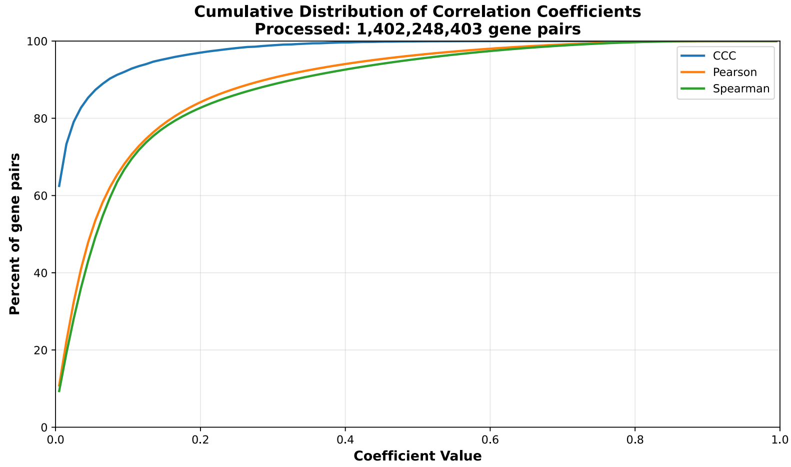

c) UpSet plot using top and bottom 30% correlations

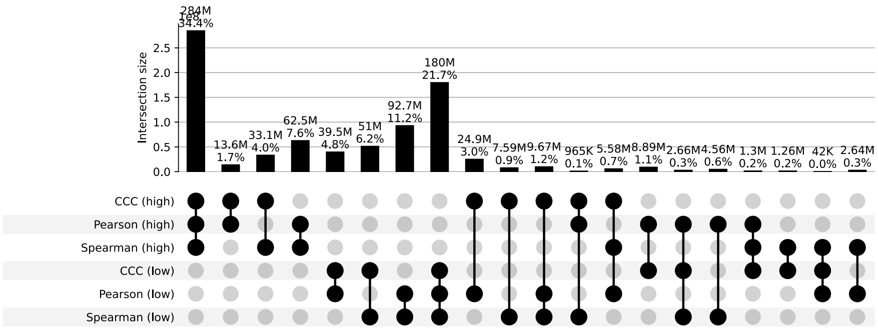

d) UpSet plot using permutation-based statistical thresholds

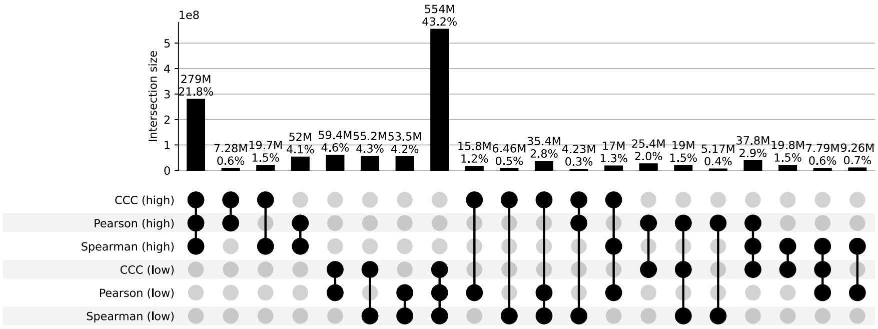

Figure S54: Distribution and UpSet plots for GTEx v8 stomach.

Testis

a) Correlation coefficient distributions between gene pairs within GTEx v8 Testis

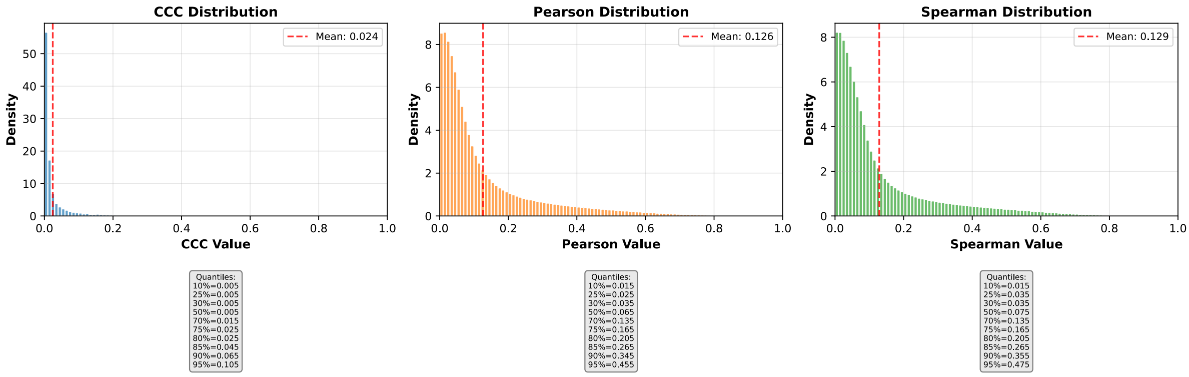

b) Corresponding cumulative histogram

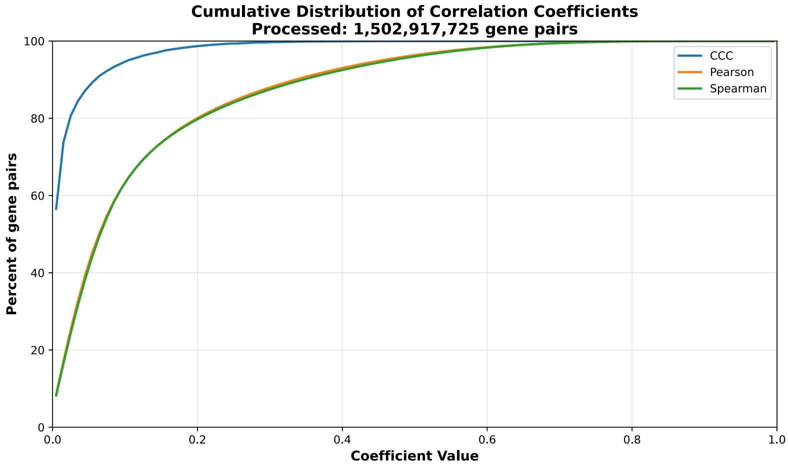

c) UpSet plot using top and bottom 30% correlations

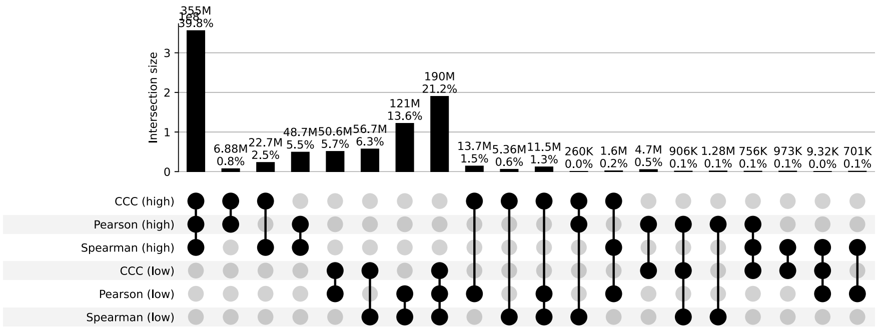

d) UpSet plot using permutation-based statistical thresholds

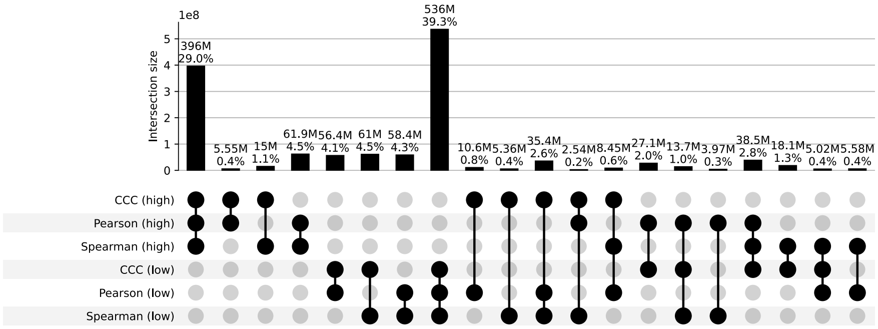

Figure S55: Distribution and UpSet plots for GTEx v8 testis.

Thyroid

a) Correlation coefficient distributions between gene pairs within GTEx v8 Thyroid

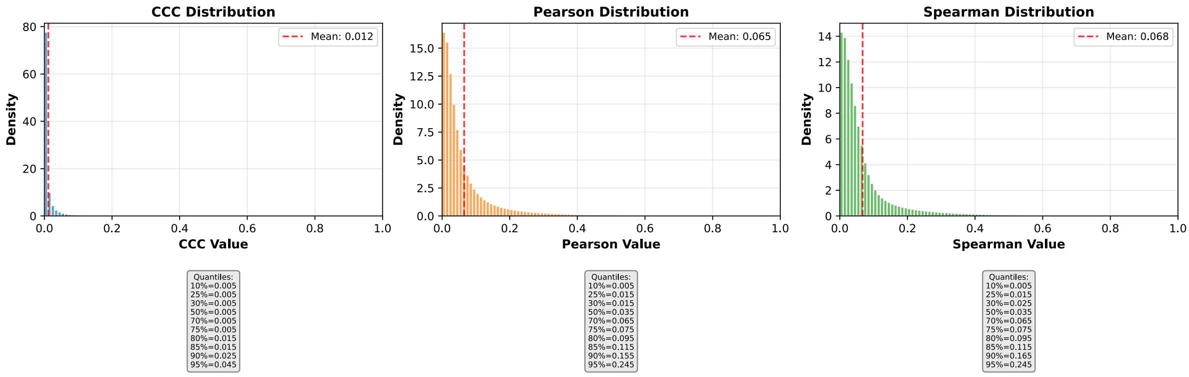

b) Corresponding cumulative histogram

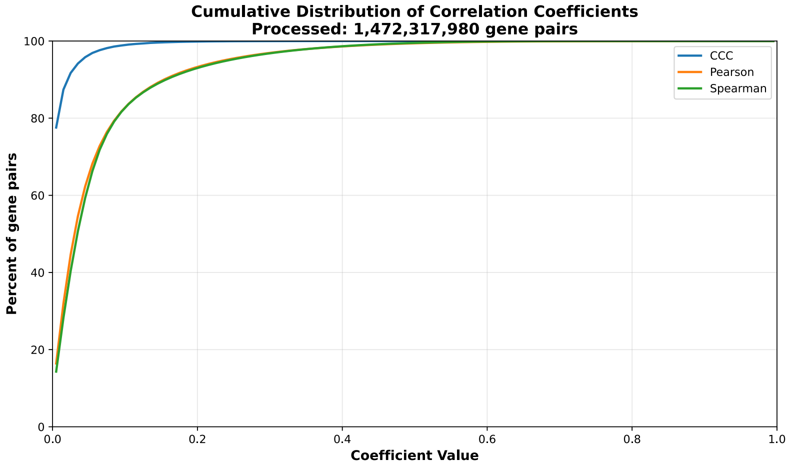

c) UpSet plot using top and bottom 30% correlations

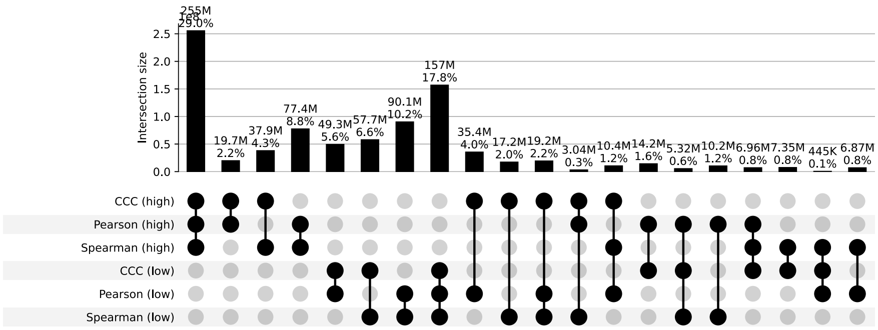

d) UpSet plot using permutation-based statistical thresholds

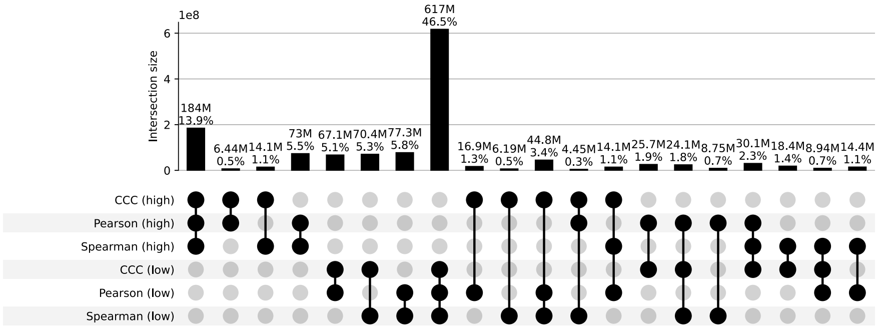

Figure S56: Distribution and UpSet plots for GTEx v8 thyroid.

Uterus

a) Correlation coefficient distributions between gene pairs within GTEx v8 Uterus

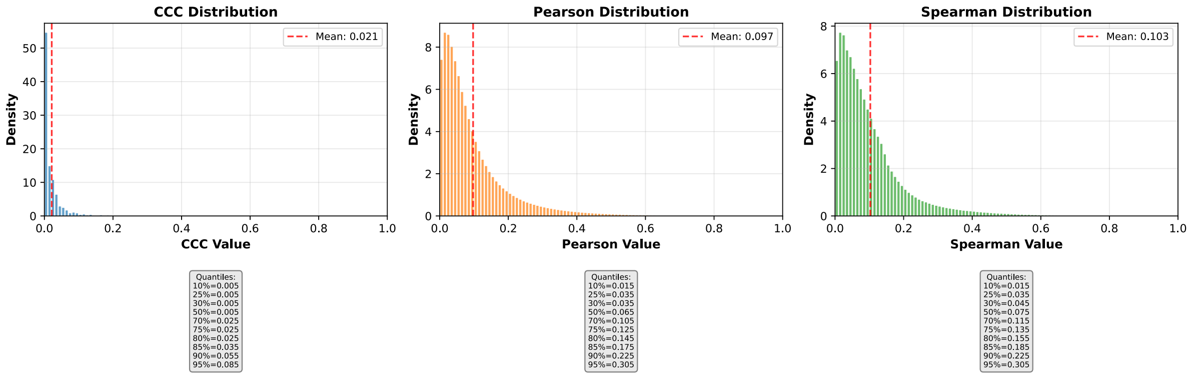

b) Corresponding cumulative histogram

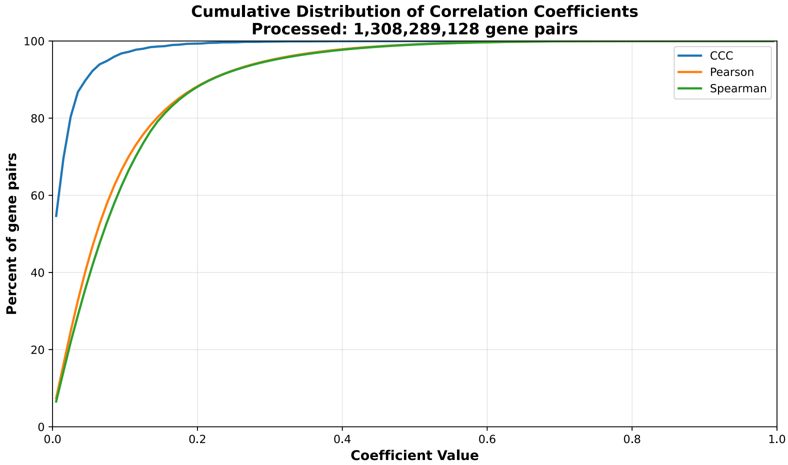

c) UpSet plot using top and bottom 30% correlations

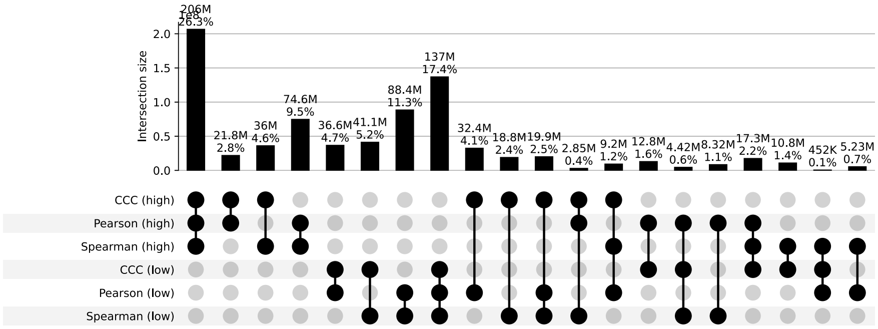

d) UpSet plot using permutation-based statistical thresholds

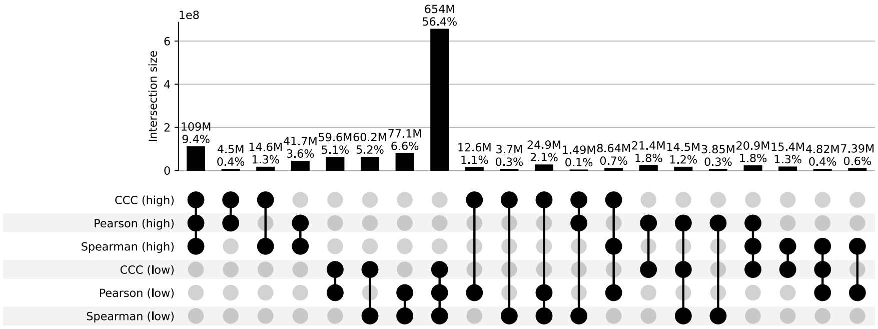

Figure S57: Distribution and UpSet plots for GTEx v8 uterus.

Vagina

a) Correlation coefficient distributions between gene pairs within GTEx v8 Vagina

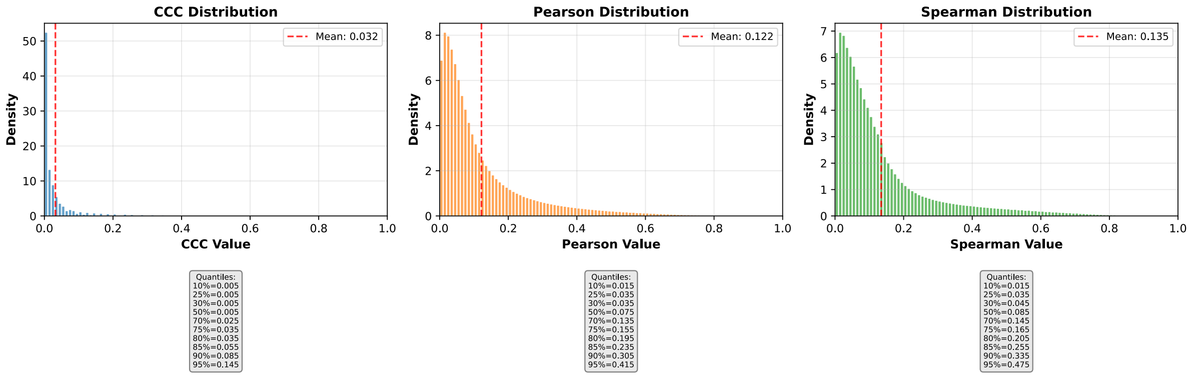

b) Corresponding cumulative histogram

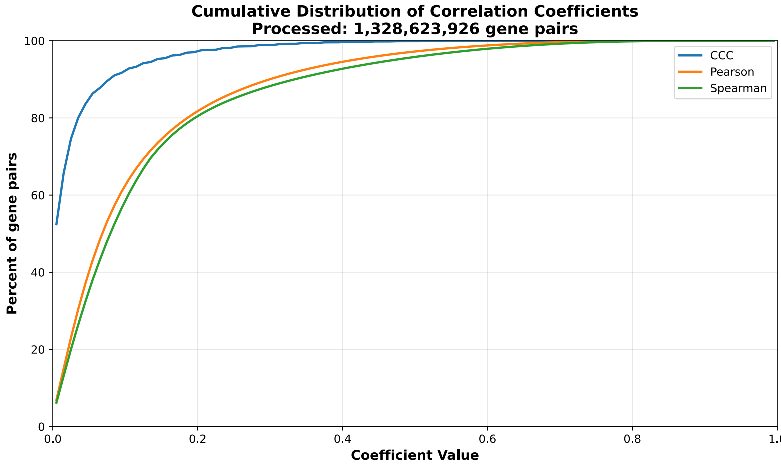

c) UpSet plot using top and bottom 30% correlations

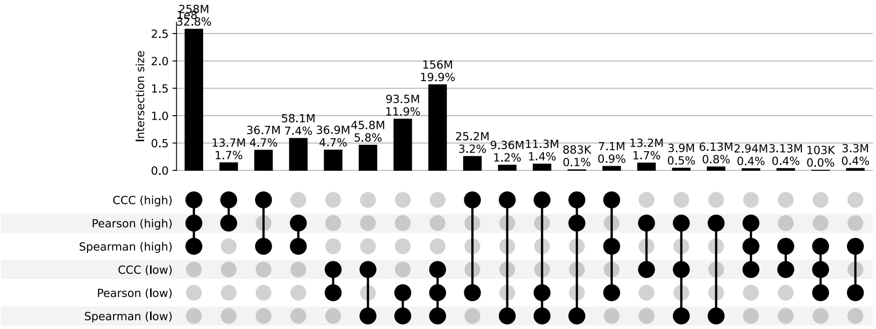

d) UpSet plot using permutation-based statistical thresholds

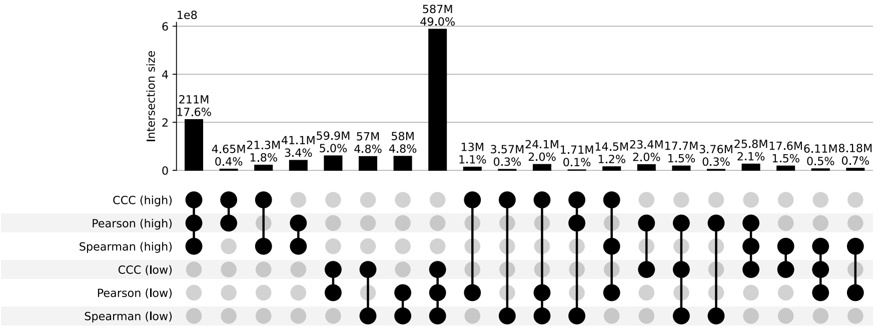

Figure S58: Distribution and UpSet plots for GTEx v8 vagina.

Whole Blood

a) Correlation coefficient distributions between gene pairs within GTEx v8 Whole Blood

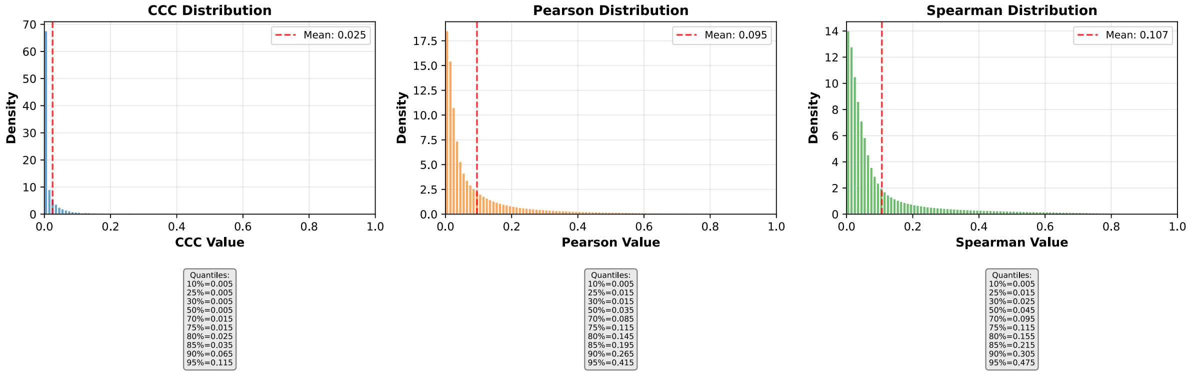

b) Corresponding cumulative histogram

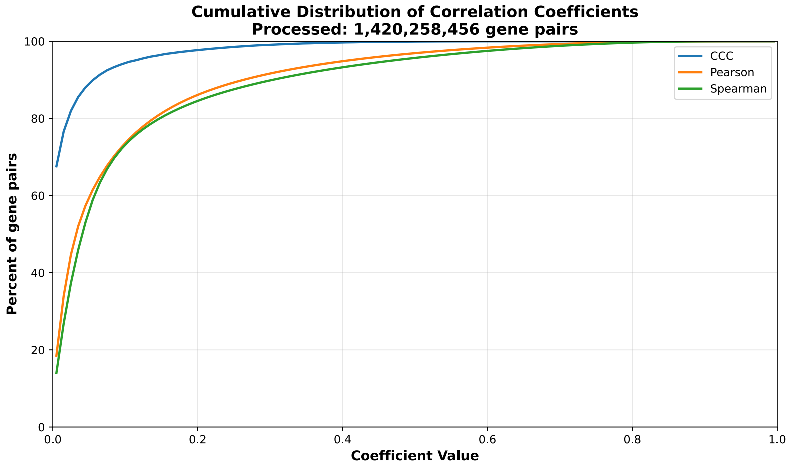

c) UpSet plot using top and bottom 30% correlations

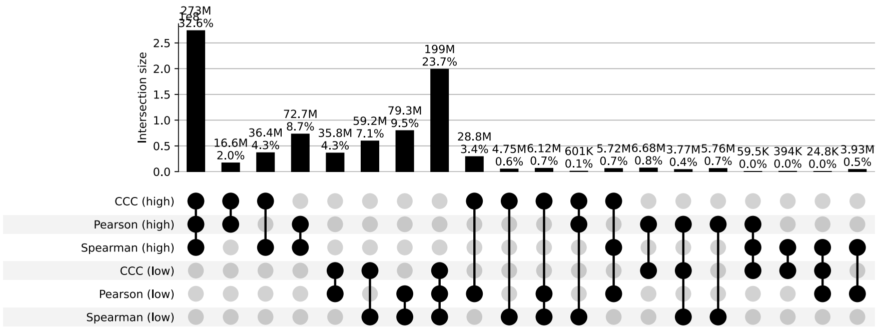

d) UpSet plot using permutation-based statistical thresholds

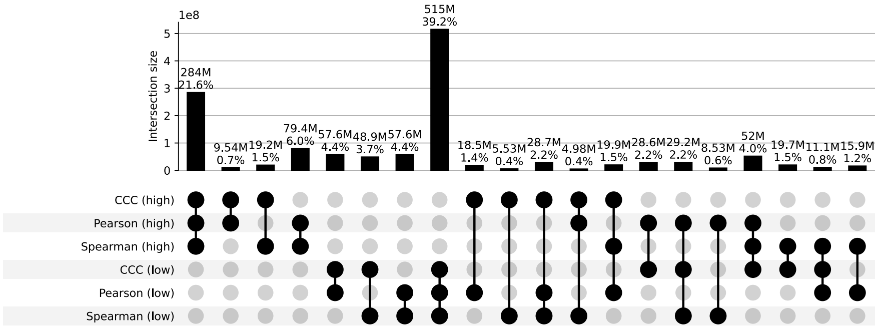

Figure S59: Distribution and UpSet plots for GTEx v8 whole blood.
